# Biotic interchange between the Indian subcontinent and mainland Asia through time

**DOI:** 10.1038/ncomms12132

**Published:** 2016-07-04

**Authors:** Sebastian Klaus, Robert J. Morley, Martin Plath, Ya-Ping Zhang, Jia-Tang Li

**Affiliations:** 1 Chengdu Institute of Biology, Chinese Academy of Sciences, Chengdu 610041, China; 2 Department of Ecology and Evolution, J. W. Goethe University, Max-von-Laue-Strasse 13, 60438 Frankfurt am Main, Germany; 3 Palynova Ltd, 1 Mow Fen Road, Littleport, Cambs CB6 1PY, UK; 4 Earth Sciences Department, Royal Holloway, University of London, Egham, Surrey TW20 0EX, UK; 5 College of Animal Science and Technology, Northwest A&F University, Yangling 712100, China; 6 State Key Laboratory of Genetic Resources and Evolution, Kunming Institute of Zoology, Chinese Academy of Sciences, Kunming 650223, China

## Abstract

Biotic interchange after the connection of previously independently evolving floras and faunas is thought to be one of the key factors that shaped global biodiversity as we see it today. However, it was not known how biotic interchange develops over longer time periods of several million years following the secondary contact of different biotas. Here we present a novel method to investigate the temporal dynamics of biotic interchange based on a phylogeographical meta-analysis by calculating the maximal number of observed dispersal events per million years given the temporal uncertainty of the underlying time-calibrated phylogenies. We show that biotic influx from mainland Asia onto the Indian subcontinent after Eocene continental collision was not a uniform process, but was subject to periods of acceleration, stagnancy and decrease. We discuss potential palaeoenvironmental causes for this fluctuation.

## Abstract (web summary)

The Paleogene continental collision between the Indian subcontinent and Asia initiated biotic interchange over a timescale of millions of years. Klaus *et al*. develop a phylogeographic method to estimate the dynamics of biotic interchange over these timescales, revealing periods of acceleration, stagnation and decline.

## Graphical abstract

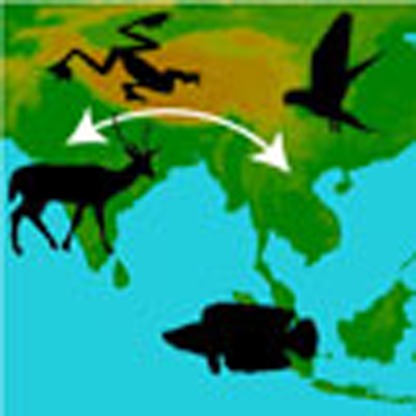

On a geological timescale, biotic interchange is considered to be a key factor that shaped the current composition of global biodiversity^1^. The opening of dispersal corridors not only allows for range expansions, but most probably also increases species diversification^2^,^3^. Several major events that allowed the sudden expansion of terrestrial floras and faunas are known from the Cenozoic, such as the Great American Biotic Interchange after the Neogene closure of the Isthmus of Panama^4^, the Pleistocene closure of the Bering Strait^5^ and the Eocene Indian–Eurasian collision^6^. It is recognized, however, that during these events not only the physical connection of continental plates influenced biotic exchange but also ecological factors that either promote or hamper dispersal^7^. Under the premise of phylogenetic niche conservatism, dispersal should be favoured along corridors that represent the same environment inhabited by the source biota. Such a pattern was shown for North American mammals that entered South America along a Savannah corridor^4^ and for South American woody plants that retained their ancestral North American microhabitat^8^.

As most available data stem from the rather recent (Late Miocene or Pliocene) Great American Biotic Interchange, the development and magnitude of biotic interchange over larger geological time spans have not been addressed so far. In addition, adequate tools for investigating the temporal dynamics of biotic interchange that do not rely on the fossil record have hardly been explored. In this context, the biotic interchange following the continental collision between the Indian subcontinent and mainland Asia can serve as a model system that can provide important new insights. The Indian subcontinent, once part of Gondwana, approached and finally collided with Eurasia during the Eocene^9^,^10^, although some authors favour an earlier^11^ or later^12^ final collision. The resulting rise of the Himalaya and Tibetan plateau triggered environmental changes of global impact following the development of the extant Asian monsoon system. Consequently, the Indian subcontinent and adjacent Asia underwent several environmental shifts between perhumid, seasonal tropical and arid climates^13^,^14^.

Our aim was to investigate (1) the onset of biotic interchange between mainland Asia and the Indian subcontinent, (2) whether dispersal between the Indian subcontinent and mainland Asia was a uniform process over time and (3) whether potential fluctuation in dispersal patterns would correlate with palaeoenvironmental changes. Decelerating diversification in the source biota is likely to result in fewer species that might colonize the new range (and thus, fewer colonization events overall). Given that climate can influence the degree of species diversification^15^, it is likely to have a strong impact on biotic interchange. In addition, it was shown that species expand their ranges along dispersal corridors with similar environmental conditions as encountered by their source biota^4^,^8^. As perhumid warm climates globally are associated with highest species numbers^16^,^17^ and hence highest rates of diversification^18^,^19^, we should expect that highest dispersal rates will occur when originating and recipient areas are characterized by perhumid climates, with reductions in dispersals occurring when climates in the donor biota are seasonally dry.

To reconstruct the magnitude of dispersal between both areas, we compiled age estimates of 127 range shifts based on 37 dated phylogenies. The majority of Asian taxa involved in these dispersal events were Southeast (SE) Asian (76%; compared with 15% East Asian taxa and 9% from the Middle East). We extracted credibility intervals of dispersal times and cumulated these over time slices of one million year, resulting in the maximal number of dispersal events (MDEs) per each one million year bin for biotic exchange between mainland Asia and the Indian subcontinent. Owing to extinction and sampling bias, the MDE does not necessarily represent the number of actual dispersal events per million years (Myr). Instead, it should be interpreted as the maximal number of observed dispersal events per Myr at a given time point based on the temporal uncertainty of the underlying time-calibrated phylogenies. We estimated change points in the development of MDE from 70 Ma to the present, to track dispersal dynamics over time. We show that biotic interchange between the Indian subcontinent and mainland Asia was a dynamic process. It accelerated at 44 Ma, pointing to a continuous dispersal corridor since that time, peaking during the Middle Miocene, coinciding with the Mid Miocene Climatic Optimum, and decreasing after 14 Ma, when drier environmental conditions developed in northern India.

## Results

### Accelerated biotic interchange since the Eocene

**Figure 1:**
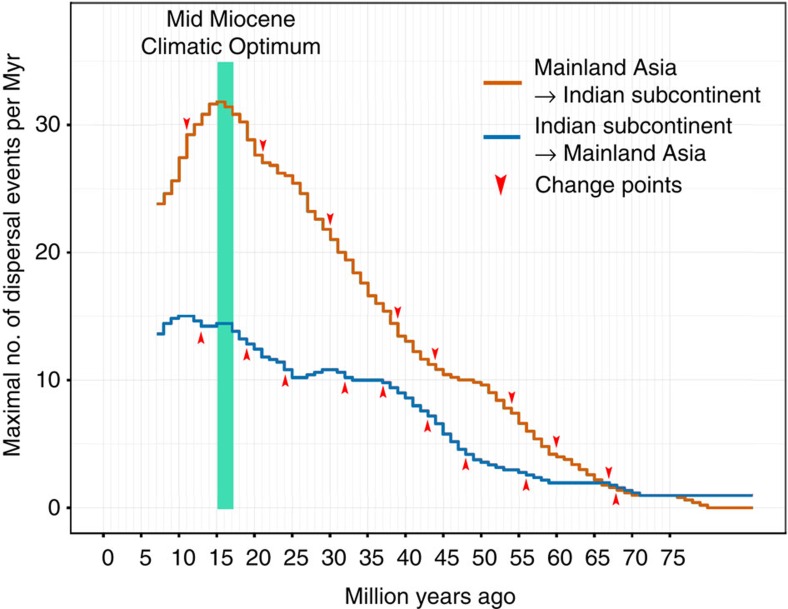
Development of the MDEs for biotic interchange between mainland Asia and the Indian subcontinent. Arrowheads indicate estimated change points. Increase of MDE between 45 and 40 Ma points to a complete terrestrial connection between colliding continents. Periods of stagnant MDE coincide with intensification of the monsoon system and increased seasonality, whereas the strong decrease in MDE after the Mid Miocene Climatic Optimum might be elicited by increasing aridity in northern India.

**Figure 2:**
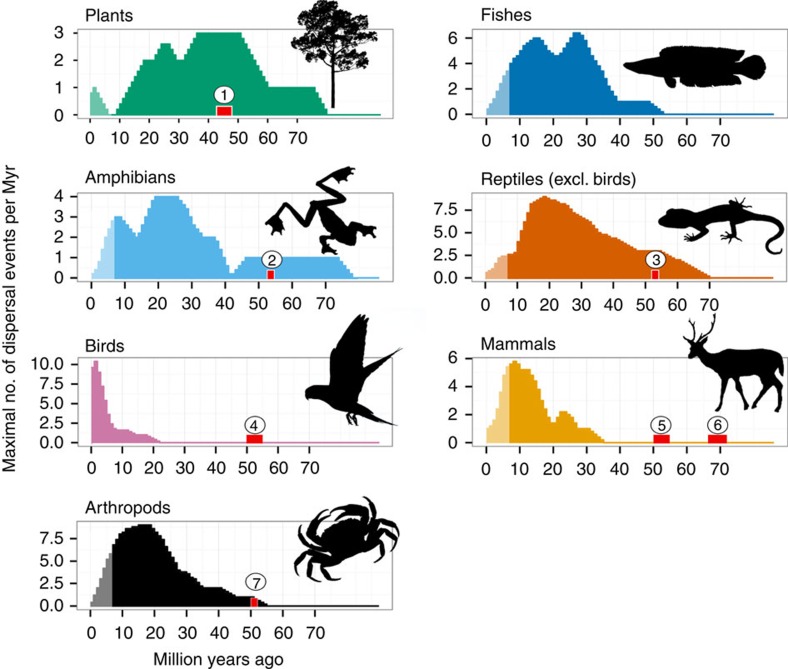
MDEs for different groups of organisms and early fossil evidence for biotic interchange between the Indian subcontinent and mainland Asia. Taxa involved in early biotic interchange (>40 Ma; plants and reptiles) are also more likely to be capable of surmounting marine passages than the other taxa. The late increase of MDE in birds is most likely a bias in the data that is focused on avian dispersal within genera and subspecies. Red bars indicate (1) dispersal of Indian floral elements into SE Asia based on fossil evidence, (2) first occurrence of anurans^60^, (3) agamids^61^, (4) psittaciform birds^25^, (5) primates, lagomorphs, artio- and perissodactyls^26^, (6) euarchontan-like and ungulate mammals^26^ and (7) various arthropod groups^62^ on the Indian subcontinent.

The MDE from the Indian subcontinent to mainland Asia started to increase at 48 Ma (Fig. 1). This would be in line with most geological studies dating the Indian–Eurasian collision at around 50 Ma, immediately followed by cessation of marine sedimentation and the closure of the Neotethys Ocean^9^,^20^. In contrast, the MDE from mainland Asia to the Indian subcontinent increased slowly until 54 Ma, followed by a stagnant MDE until about 44 Ma (Fig. 1). The first appearance of angiosperms of Indian origin in Sundaland is reported at ∼49 Ma, with increased dispersal after 45 Ma^13^. The increased MDE from India towards mainland Asia, coupled with stagnant MDE values for the opposite dispersal direction, would be consistent with the suggestion of an aggressive Indian biota, which had become well-adapted to warm and wet early Eocene equatorial climates, extensively replacing the SE Asian biota^14^,^21^. However, as there were few dispersal events of taxa specifically tied to freshwater habitats during this period, such as amphibians and freshwater fishes, dispersal was most likely to be *trans*-oceanic (Fig. 2).

### Evidence for a continuous dispersal corridor after 44 Ma

MDE rose for dispersal from mainland Asia to India after 44 Ma. Coupled with the observed increase in dispersal events of amphibians at 41 Ma and primary freshwater fishes at 38 Ma, we suggest that this sudden change points to a continuous terrestrial dispersal corridor not earlier than 44 Ma. A terrestrial connection around this time would be in good agreement with a continental collision during the Middle to Late Eocene, as favoured by some authors^10^,^22^,^23^. Alternatively—as most dispersal events recorded here are between the Indian subcontinent and SE Asia—it may also relate to asynchronous continental collision and Neotethys closure, starting first at the western edge of the Indian subcontinent^24^, such that the terrestrial connection to tropical SE Asia would have been established last. A Late Cretaceous/Palaeocene^11^ or Early Miocene^12^ continental collision, however, cannot explain the dispersal pattern we describe here. Notably, the taxa that showed increased MDE before 44 Ma are non-avian reptiles and plants that are more likely to surmount marine passages, in contrast to amphibians and primary freshwater fishes. Occasional *trans*-oceanic dispersal is also more likely to explain the occurrence of Early Eocene fossil birds^25^ and mammals^26^ on the Indian subcontinent (Fig. 2).

From 39 to 30 Ma, MDE from Asia to India shows a virtually linear increase over time, whereas dispersal events from India to Asia increase until 37 Ma and stagnate until about 24 Ma. From 24 Ma until 21 Ma, MDE shows a reversed pattern; dispersal from India increases, whereas dispersal from Asia decelerates. We can thus clearly reject the idea that biotic interchange increases over time at a uniform rate after the establishment of a dispersal corridor.

Detailed palaeoclimatological data for the Indian subcontinent from the Eocene to Early Miocene are as yet lacking. It can be inferred, however, that between 44 and 39 Ma, when central India was situated just north of the equator, both India and SE Asia were most probably characterized by similar perhumid climates^27^ and strong dispersal in both directions would be anticipated. After the Indian subcontinent moved further north^22^, distinctly seasonal climates are a probable scenario for wide areas of northern India^28^. Thus, reduced dispersal from India to Asia could be explained by decelerated phylogenetic diversification during the establishment of more seasonal conditions in the source biota. The same applies for the interval from 24 to 21 Ma, when MDE to the Indian subcontinent from Asia was stagnant, as there is good evidence that the climate in northern mainland SE Asia was warm temperate during the Oligocene and Early Miocene^21^,^29^,^30^, changing again to more perhumid climates only during the Middle Miocene^30^.

### A Middle Miocene peak of biotic interchange

We found a sharp increase of MDE from mainland Asia to the Indian subcontinent between 21 and 11 Ma, peaking at 15 Ma. This coincides with the global Mid Miocene Climatic Optimum^31^,^32^ (17–14 Ma), as well as with the establishment of a rain forest belt along the lower Himalayan foothills from Burma to Bhutan in the course of the Himalayan uplift^21^,^31^,^32^ (represented today by a narrow belt of seasonal evergreen dipterocarp forests^33^,^34^). During the Middle Miocene the monsoon system was probably weaker^35^ and with globally warmer climates this allowed the establishment of widespread evergreen rain forests across northern India, reflected by abundant macrofossils, many of which are of SE Asian origin^14^,^36^,^37^,^38^. We propose that during the Middle Miocene, with both northern India and SE Asia being again characterized by more perhumid climates^13^,^14^, a second continuous dispersal corridor formed from mainland SE Asia to northern India along the Himalayan foothills.

### Decrease of biotic interchange since 14 Ma

From 14 Ma onwards, MDE decreased, coinciding with a shift to strongly seasonal climates in northern India, an expansion of grasslands after 8 Ma^39^, and finally the establishment of arid climates in northwest India following global cooling and drying^40^,^41^. In addition, in SE Asia an increase in climatic seasonality associated with the development of the Indian monsoon can be inferred during the Late Miocene^13^,^42^, potentially negatively affecting the leading edge dispersal within the dispersal corridor.

This study provides novel insights into the dynamics of biotic interchange between the Indian subcontinent and mainland Asia over a period of more than 50 million years using a new method that may be applicable to other areas where biotic interchange over time is expected. We emphasise that especially (i) the similarity of climate between originating and recipient areas and (ii) the potential influence of climate on phylogenetic diversification in the donor biota may affect the pattern of biotic interchange along a dispersal corridor.

## Methods

### Phylogenetic methods and divergence time estimations

We re-calculated divergence times of 37 phylogenetic data sets (Supplementary Fig. 1; for details of the analyses, see Supplementary Note 1) using sequences obtained either from GenBank, as alignments from TreeBASE or from the authors directly. Divergence time analyses were conducted with the software BEAST MC3 v.1.7.5 (ref. 43) running three chains (*δ*=1.0), applying a Yule tree prior and an uncorrelated relaxed molecular clock model. Data sets were partitioned and the best-fitting models of sequence evolution applied as suggested in PartitionFinder v.1.1.1 (ref. 44) based on the Akaike Information Criterion. The log-files of the Bayesian analyses were checked in Tracer v.1.5 (ref. 45) for autocorrelation and stationarity of the sampled parameters. We ran the analyses for 50,000,000 iterations and sampled every 10,000th iterations; 10% of the sampled iterations were discarded as ‘burn-in' unless stated otherwise (see Supplementary Note 1). We investigated the influence of prior assumptions on the results by sampling from the prior only.

### Ancestral area estimation

To estimate the temporal patterns of dispersal maxima and minima, we compiled 128 credibility intervals of divergence times (Supplementary Table 1) at nodes for which we inferred a dispersal/range shift between the Indian subcontinent and mainland Asia. Biogeographical inference was conducted in R v.3.1.1 (ref. 46) using the package BioGeoBEARS v.0.2.1 (ref. 47). We compared a dispersal–extinction–cladogenesis model (DEC)^48^ against a DEC model that allows for founder-event speciation (DEC+J)^49^ and chose the better model based on Akaike Information Criterion values (Supplementary Fig. 1). Outgroups were removed for the biogeographical analyses. In few cases, prior knowledge was implemented by either weighting dispersal between areas via dispersal multipliers or constraining the root node to a certain area; the area coding generally follows the original studies with few exceptions (see Supplementary Note 1). The area of the Indian subcontinent was defined as the combined areas of Sri Lanka, India, Nepal, Buthan, Pakistan and Bangladesh, whereas Myanmar was coded as ‘Asian'. Taxa from the New World were collectively coded as ‘America', that is, we did not discriminate between North, Central or South America.

### Biogeographical meta-analysis

Although an increasing number of studies focussing on historical biogeography use time-calibrated phylogenies, only few attempts were made to integrate the information from different taxa to infer general patterns of biotic interchange^50^,^51^,^52^,^53^. Early, ground-breaking approaches to biogeographical meta-analysis could only approximate the temporal dimension of phylogenetic divergence. These approaches used parsimony-based methods that do not directly incorporate branch length, that is, temporal information^50^,^51^. Two more recent studies, however, explicitly incorporate time-calibrated molecular phylogenies to evaluate general patterns of past biotic interchange: Stelbrink *et al.*^52^ visualized the age of initial divergence and crown-group diversification on a timescale for 27 taxa that dispersed to and subsequently radiated on Sulawesi. De Bruyn *et al.*^53^ developed a more sophisticated approach for the island of Borneo: they inferred dispersal events using a DEC method and plotted occurrence and strength of dispersal routes for pre-selected, discrete time periods. They also extracted immigration and emigration events, and compared these statistically. Although much more advanced than previous methods, we concluded that the necessity for *a priori* defining time periods renders this method unsuited for investigating the intensity of biotic interchange between the Indian subcontinent and mainland Asia through time without making prior assumptions. As the dating of molecular phylogenies is subject to a considerable degree of uncertainty concerning the application of external rates (that have been calibrated in other studies using different taxa), or the calibration with fossils or biogeographical events, we regard it as essential to consider the temporal uncertainty attached to every chronogram (for example, in the form of credibility intervals in the case of Bayesian phylogenetic dating methods). To overcome this shortage, we developed an alternative approach. We consider that dispersal events are more likely to have happened when the corresponding divergence time intervals overlap, assuming that these dispersal events have the same (abiotic) cause(s), in line with the concept of geo-dispersal^54^. This is reflected by the maximal number of observed dispersal events per Myr (MDE).

The MDE was calculated by summing up potential dispersal events over all data sets through time using time slices of one million years based on the credibility intervals (95% highest posterior densities, rounded to the closest full million) of corresponding divergence times (as illustrated in Supplementary Fig. 2). All dispersal events were treated as independent also when they originated from the same data set/phylogeny. We discriminated between different dispersal directions, that is, dispersal from the Indian subcontinent to mainland Asia (35 events) or vice versa (92 events). In addition, we extracted dispersal times for amphibians (18 events), non-avian reptiles (22 events), birds (23 events), arthropods (21 events), plants (16 events) and teleost fishes (15 events) (Fig. 2). The divergence time credibility intervals are given in Supplementary Table 1 and the raw MDEs are shown in Supplementary Fig. 3.

To avoid over-interpretation of slight (possibly stochastic) changes in MDE, we smoothed the data by calculating mean values in a sliding window approach with a time frame of 5 Myr. One potential source of methodological artefact is a bias against younger dispersal events, as younger phylogenetic splits are more likely to escape sampling. To account for this effect we calculated the MDE from seven bird phylogenies including subspecies with a more complete taxon sampling than in most other studies. We compared the sharp increase of MDE in birds towards the present against the decreasing MDE in other taxa (plants, arthropods, teleost fishes, amphibians, non-avian reptiles and mammals) and removed the data from the time point where avian and non-avian MDE intersect (<7 Ma) (Supplementary Fig. 4). This is more conservative than the previous estimate of 5 Ma for the maximum age for the occurrence of cryptic speciation^55^.

The so-called ‘pull to the present' effect in phylogenies predicts a steady increase in lineages^56^ and thus an increased chance of dispersal events, and so we did not focus on absolute values of MDE but investigated shifts in MDE using change point analysis as implemented in the R-package ‘ecp' v. 1.6.2 (ref. 57). We used the divisive hierarchical estimation algorithm that sequentially identifies distributional changes within time-ordered observations via a bisection algorithm that is based on the divergence measure of Székely and Rizzo^58^,^59^. The ‘e.divisive' function was run with the following parameters: maximum number of random permutations=500 to estimate statistical significance of change points; significance level=0.05; minimum number of observations (that is, MDE data points) between change points=5 (to prevent excessive identification of smale-scale changes), moment index *α* used to determine the distance between and within segments=1.0).

### Data availability

All relevant data are available from the authors.

## Additional information

**How to cite this article:** Klaus, S. *et al.* Biotic interchange between the Indian subcontinent and mainland Asia through time. *Nat. Commun.* 7:12132 doi: 10.1038/ncomms12132 (2016).

## Supplementary Material

Supplementary Information Supplementary Figures 1-4, Supplementary Table 1, Supplementary Notes 1 and Supplementary References
