# Supplementary material for: Biotic interchange between the Indian subcontinent and mainland Asia through time
Source: Nat Commun. 2016 Jul 4;7:12132. doi: 10.1038/ncomms12132 (PMC4932189; doi:10.1038/ncomms12132)
Supplement: Supplementary Information — Supplementary Figures 1-4, Supplementary Table 1, Supplementary Notes 1 and Supplementary References [file ncomms12132-s1.pdf]

Supplementary Figure 1

Suppl. Fig. 1 | Time calibrated phylogenetic trees with annotated biogeographical range estimates used to infer dispersal events between the Indian subcontinent and mainland Asia. The 37 phylogenies are shown once with the best range estimate and once with the relative probability for the respective areas/ranges; annotated with the model used (DEC or DEC+*j*), the maximum number of areas allowed within a range, the resulting extinction rate (*e*), dispersal rate (*d*), founder-event weight (*j*), and the log likelihood (*lnL*).

Crypteroniaceae BioGeoBEARS DEC+J
ancstates: global optim, 3 areas max. d=0; e=0; j=0.0635; LnL=−21.29

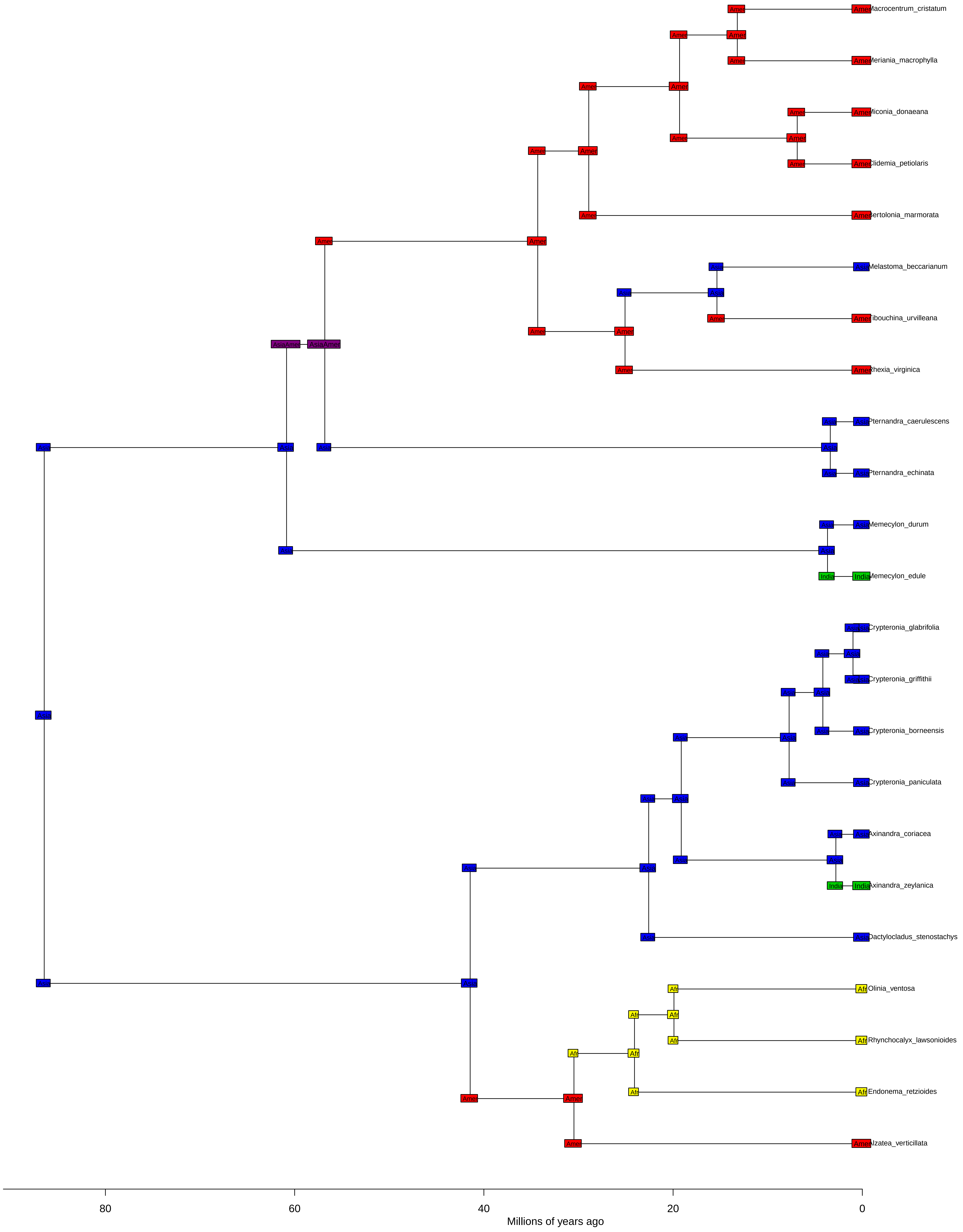

Crypteroniaceae BioGeoBEARS DEC+J
ancstates: global optim, 3 areas max. d=0; e=0; j=0.0635; LnL=−21.29

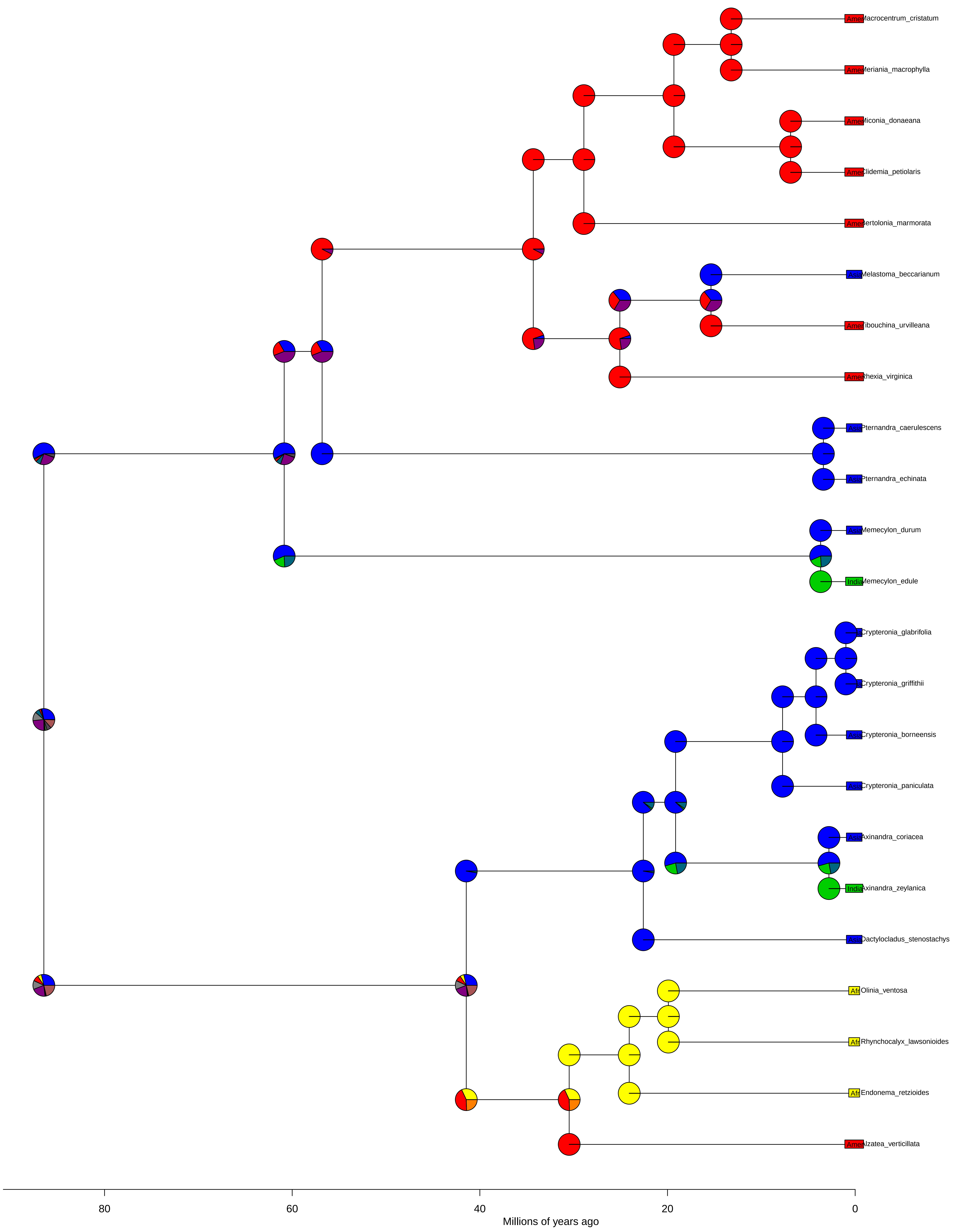

Dipterocarpaceae BioGeoBEARS DEC+J
ancstates: global optim, 2 areas max. d=0; e=0; j=0.0823; LnL=−53.72

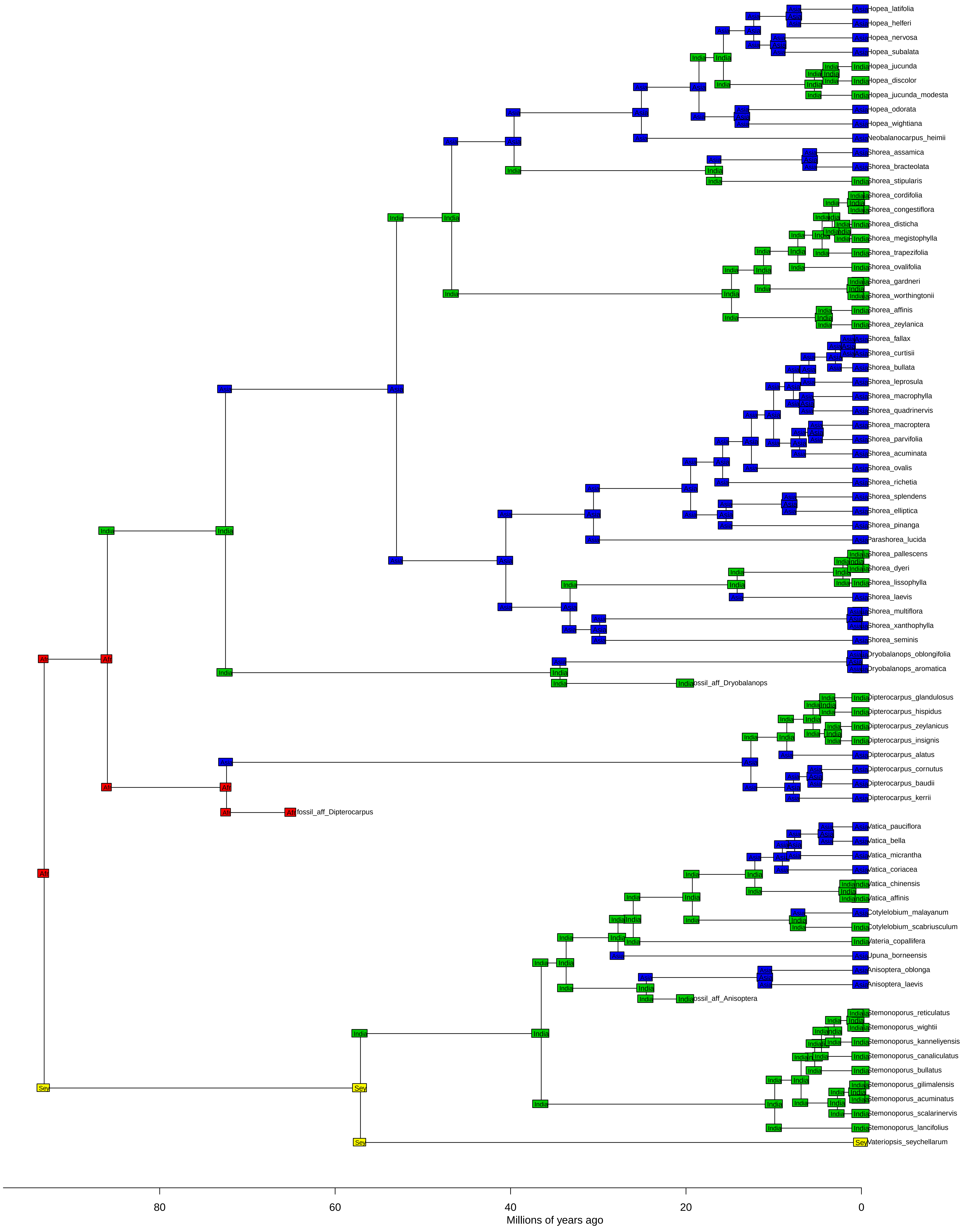

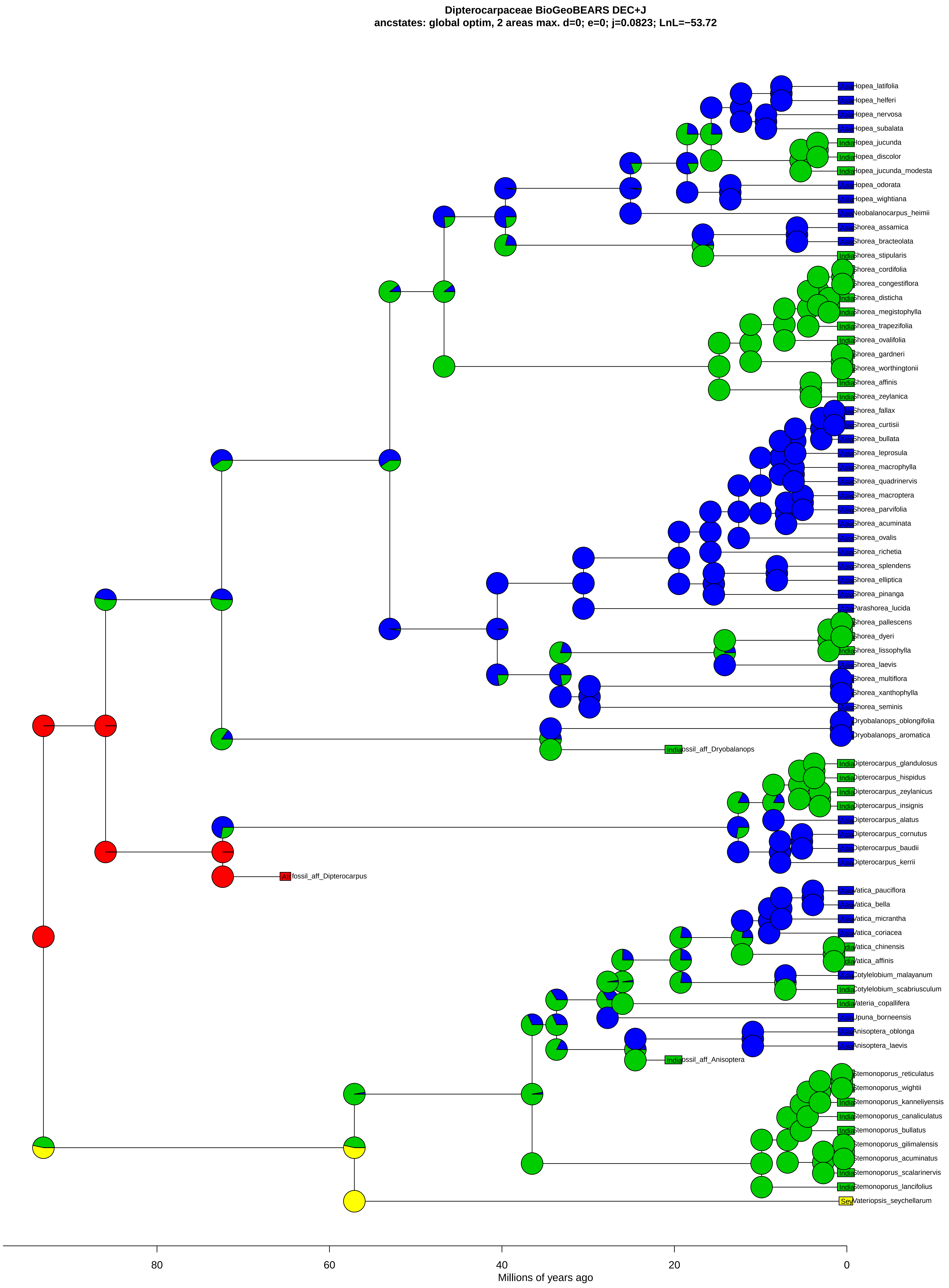

Dipterocarpaceae BioGeoBEARS DEC+J
ancstates: global optim, 2 areas max. d=0; e=0; j=0.0823; LnL=−53.72
Hopea_latifolia
Hopea_helferi
Hopea_nervosa
Hopea_subalata
Hopea_jucunda
Hopea_discolor
Hopea_jucunda_modesta
Hopea_odorata
Hopea_wightiana
Neobalanocarpus_heimii
Shorea_assamica
Shorea_bracteolata
Shorea_stipularis
Shorea_cordifolia
Shorea_congestiflora
Shorea_disticha
Shorea_megistophylla
Shorea_trapezifolia
Shorea_ovalifolia
Shorea_gardneri
Shorea_worthingtonii
Shorea_affinis
Shorea_zeylanica
Shorea_fallax
Shorea_curtisii
Shorea_bullata
Shorea_leprosula
Shorea_macrophylla
Shorea_quadrinervis
Shorea_macroptera
Shorea_parvifolia
Shorea_acuminata
Shorea_ovalis
Shorea_richetia
Shorea_splendens
Shorea_elliptica
Shorea_pinanga
Parashorea_lucida
Shorea_pallescens
Shorea_dyeri
Shorea_lissophylla
Shorea_laevis
Shorea_multiflora
Shorea_xanthophylla
Shorea_seminis
Dryobalanops_oblongifolia
Dryobalanops_aromatica
fossil_aff_Dryobalanops
Dipterocarpus_glandulosus
Dipterocarpus_hispidus
Dipterocarpus_zeylanicus
Dipterocarpus_insignis
Dipterocarpus_alatus
Dipterocarpus_cornutus
Dipterocarpus_baudii
Dipterocarpus_kerrii
fossil_aff_Dipterocarpus
Vatica_pauciflora
Vatica_bella
Vatica_micrantha
Vatica_coriacea
Vatica_chinensis
Vatica_affinis
Cotylelobium_malayanum
Cotylelobium_scabriusculum
Vateria_copallifera
Upuna_borneensis
Anisoptera_oblonga
Anisoptera_laevis
fossil_aff_Anisoptera
Stemonoporus_reticulatus
Stemonoporus_wightii
Stemonoporus_kanneliyensis
Stemonoporus_canaliculatus
Stemonoporus_bullatus
Stemonoporus_gilimalensis
Stemonoporus_acuminatus
Stemonoporus_scalarinervis
Stemonoporus_lancifolius
Vateriopsis_seychellarum
80
60
40
20
0
Millions of years ago

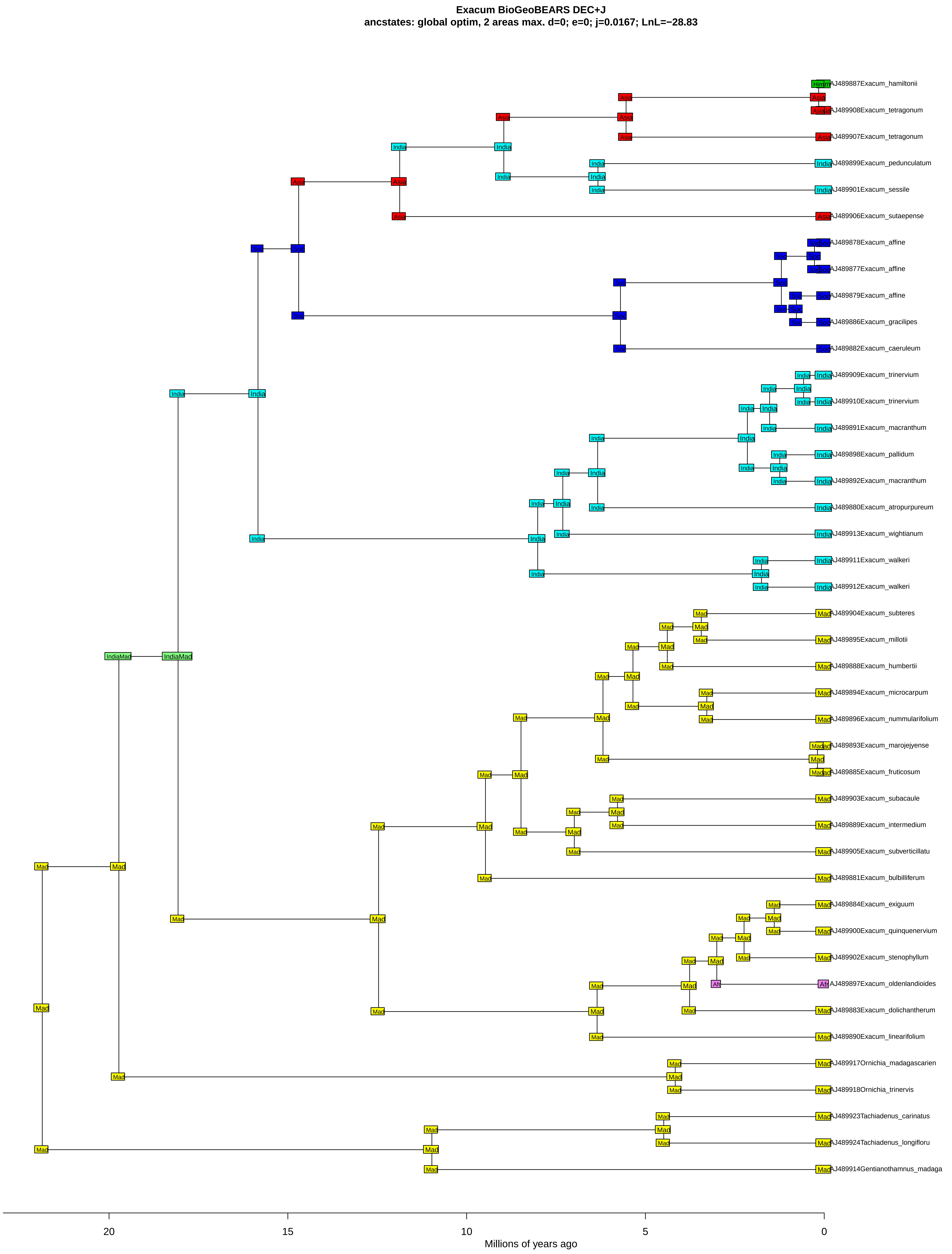
Exacum BioGeoBEARS DEC+J
ancstates: global optim, 2 areas max. d=0; e=0; j=0.0167; LnL=−28.83
AJ489887Exacum_hamiltonii
AJ489908Exacum_tetragonum
AJ489907Exacum_tetragonum
AJ489899Exacum_pedunculatum
AJ489901Exacum_sessile
AJ489906Exacum_sutaepense
AJ489878Exacum_affine
AJ489877Exacum_affine
AJ489879Exacum_affine
AJ489886Exacum_gracilipes
AJ489882Exacum_caeruleum
AJ489909Exacum_trinervium
AJ489910Exacum_trinervium
AJ489891Exacum_macranthum
AJ489898Exacum_pallidum
AJ489892Exacum_macranthum
AJ489880Exacum_atropurpureum
AJ489913Exacum_wightianum
AJ489911Exacum_walkeri
AJ489912Exacum_walkeri
AJ489904Exacum_subteres
AJ489895Exacum_millotii
AJ489888Exacum_humbertii
AJ489894Exacum_microcarpum
AJ489896Exacum_nummularifolium
AJ489893Exacum_marojejyense
AJ489885Exacum_fruticosum
AJ489903Exacum_subacaule
AJ489889Exacum_intermedium
AJ489905Exacum_subverticillatu
AJ489881Exacum_bulbilliferum
AJ489884Exacum_exiguum
AJ489900Exacum_quinquenervium
AJ489902Exacum_stenophyllum
AJ489897Exacum_oldenlandioides
AJ489883Exacum_dolichantherum
AJ489890Exacum_linearifolium
AJ489917Ornichia_madagascarien
AJ489918Ornichia_trinervis
AJ489923Tachiadenus_carinatus
AJ489924Tachiadenus_longifloru
AJ489914Gentianothamnus_madaga
IndiaMad
20
15
10
5
0
Millions of years ago

Exacum BioGeoBEARS DEC+J
ancstates: global optim, 2 areas max. d=0; e=0; j=0.0167; LnL=−28.83

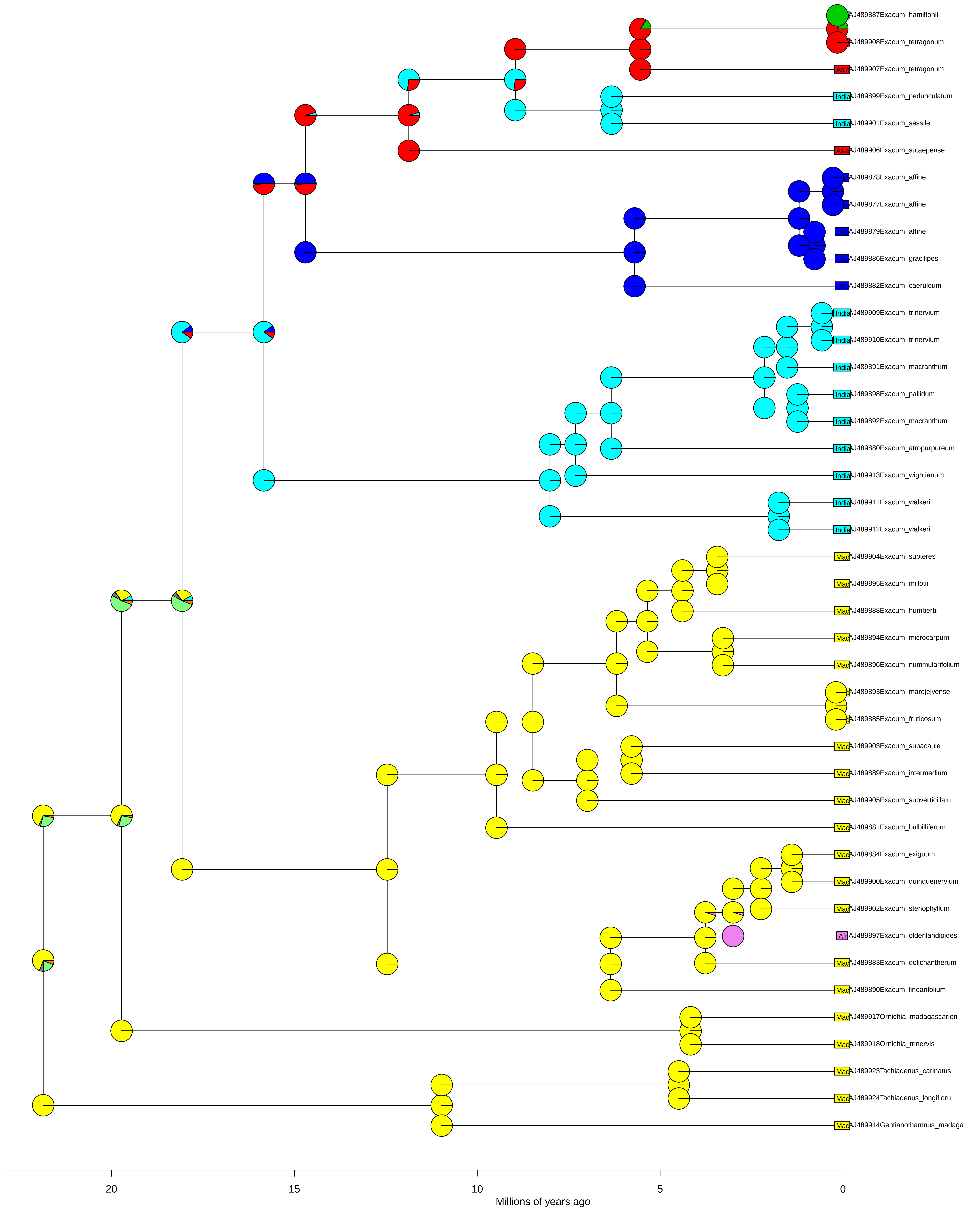

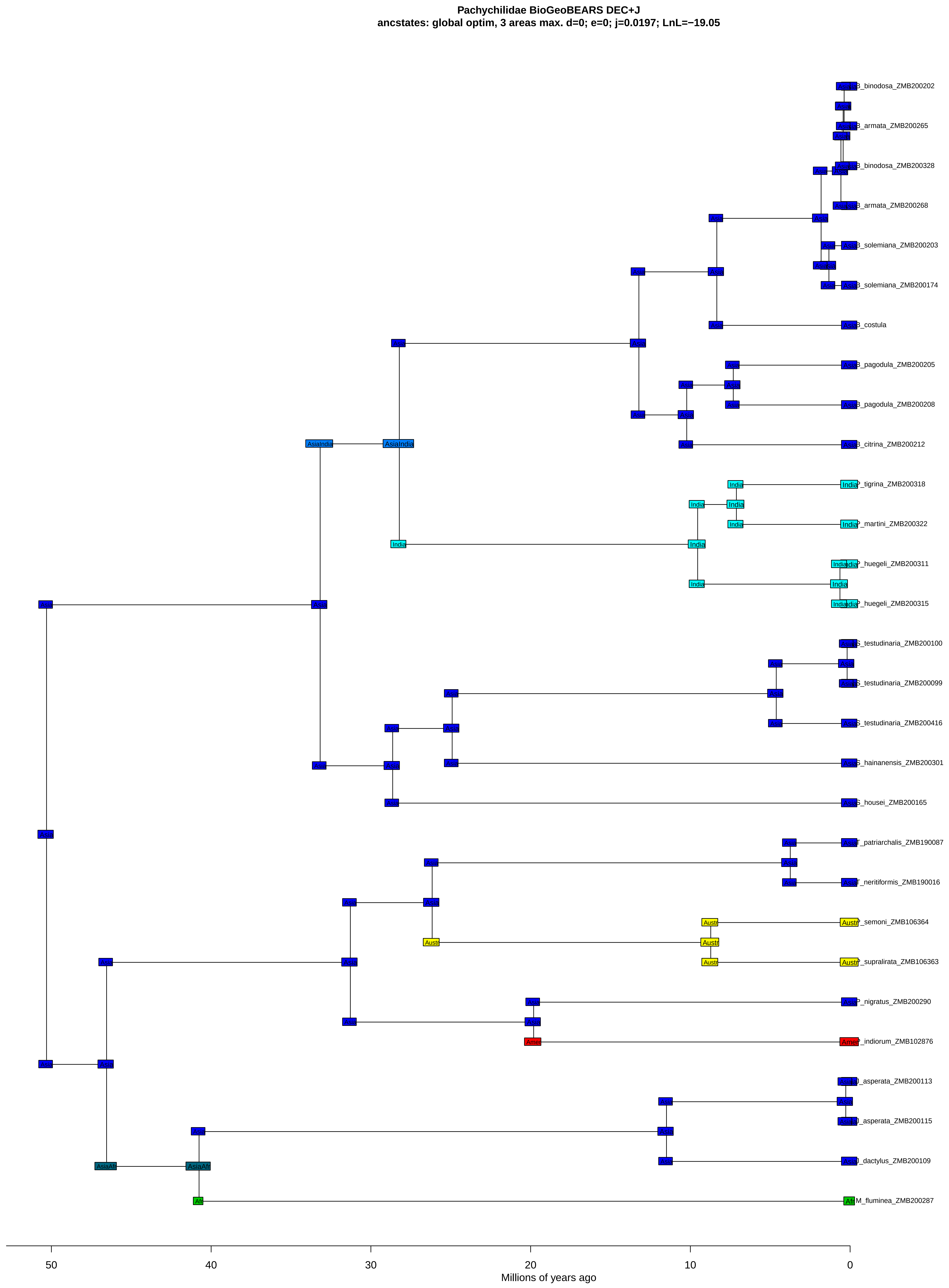
Pachychilidae BioGeoBEARS DEC+J
ancstates: global optim, 3 areas max. d=0; e=0; j=0.0197; LnL=−19.05
B_binodosa_ZMB200202
B_armata_ZMB200265
B_binodosa_ZMB200328
B_armata_ZMB200268
B_solemiana_ZMB200203
B_solemiana_ZMB200174
B_costula
B_pagodula_ZMB200205
B_pagodula_ZMB200208
B_citrina_ZMB200212
P_tigrina_ZMB200318
P_martini_ZMB200322
P_huegeli_ZMB200311
P_huegeli_ZMB200315
S_testudinaria_ZMB200100
S_testudinaria_ZMB200099
S_testudinaria_ZMB200416
S_hainanensis_ZMB200301
S_housei_ZMB200165
T_patriarchalis_ZMB190087
T_neritiformis_ZMB190016
P_semoni_ZMB106364
P_supralirata_ZMB106363
P_nigratus_ZMB200290
P_indiorum_ZMB102876
D_asperata_ZMB200113
D_asperata_ZMB200115
D_dactylus_ZMB200109
M_fluminea_ZMB200287
AsiaIndia
India
Austr
Amer
AsiaAfr
Afr
50
40
30
20
10
0
Millions of years ago

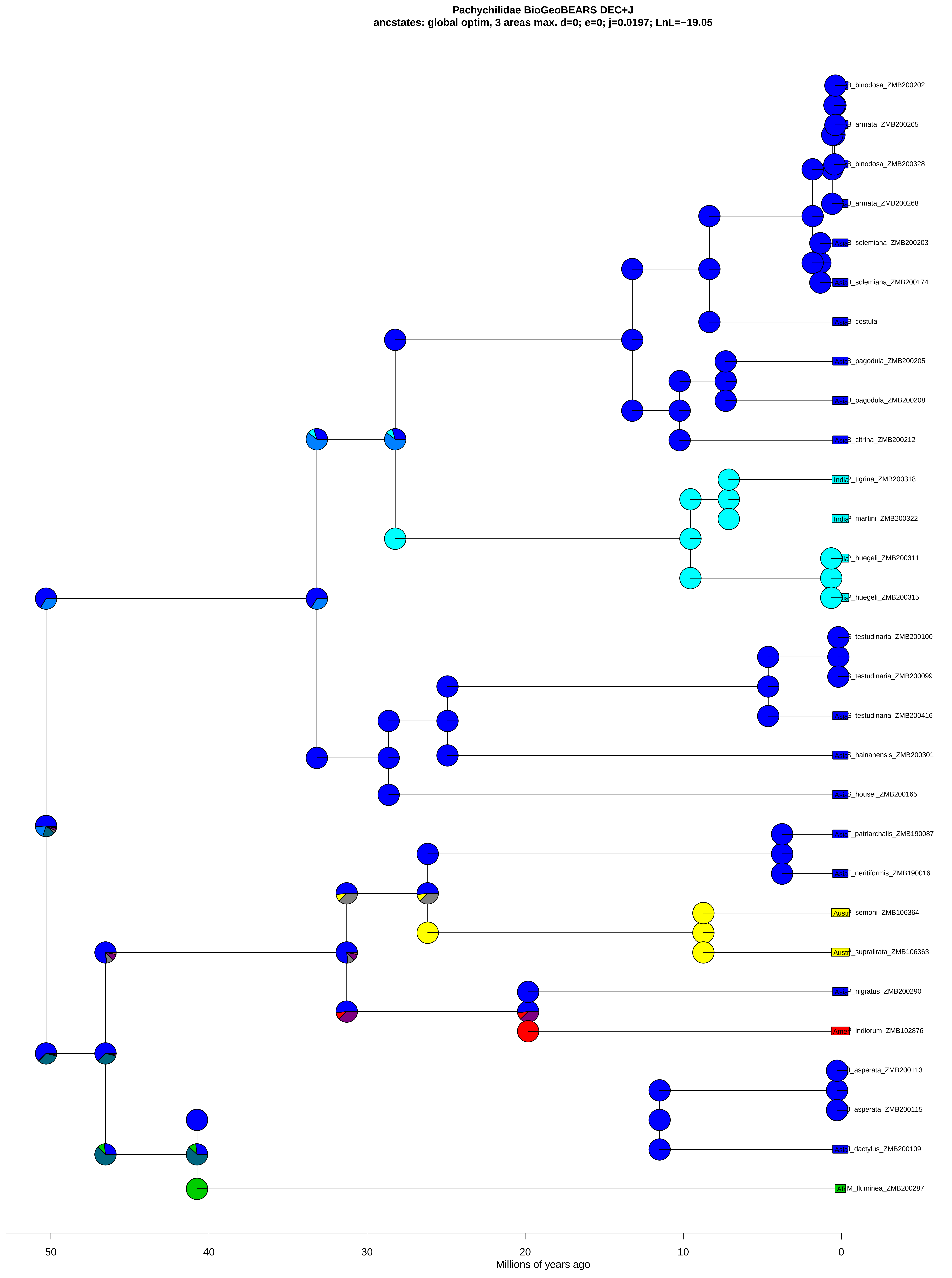

Pachychilidae BioGeoBEARS DEC+J
ancstates: global optim, 3 areas max. d=0; e=0; j=0.0197; LnL=−19.05
B_binodosa_ZMB200202
B_armata_ZMB200265
B_binodosa_ZMB200328
B_armata_ZMB200268
B_solemiana_ZMB200203
B_solemiana_ZMB200174
B_costula
B_pagodula_ZMB200205
B_pagodula_ZMB200208
B_citrina_ZMB200212
P_tigrina_ZMB200318
P_martini_ZMB200322
P_huegeli_ZMB200311
P_huegeli_ZMB200315
S_testudinaria_ZMB200100
S_testudinaria_ZMB200099
S_testudinaria_ZMB200416
S_hainanensis_ZMB200301
S_housei_ZMB200165
T_patriarchalis_ZMB190087
T_neritiformis_ZMB190016
P_semoni_ZMB106364
P_supralirata_ZMB106363
P_nigratus_ZMB200290
P_indiorum_ZMB102876
_asperata_ZMB200113
_asperata_ZMB200115
_dactylus_ZMB200109
M_fluminea_ZMB200287
India
Austr
Amer
Afr
50
40
30
20
10
0
Millions of years ago

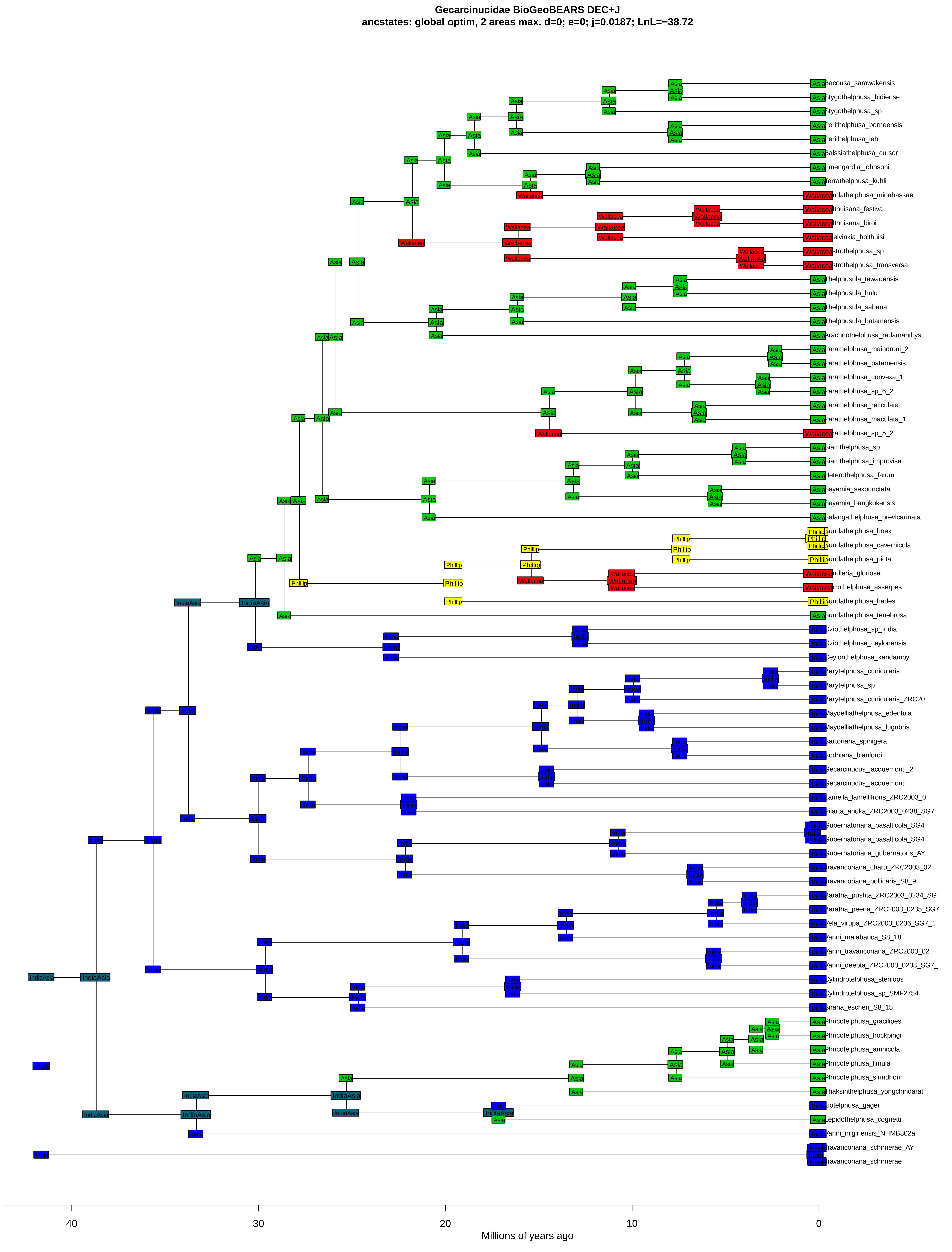
Gecarcinucidae BioGeoBEARS DEC+J
ancstates: global optim, 2 areas max. d=0; e=0; j=0.0187; LnL=−38.72
Bacousa_sarawakensis
Stygothelphusa_bidiense
Stygothelphusa_sp
Perithelphusa_borneensis
Perithelphusa_lehi
Balssiathelphusa_cursor
rmengardia_johnsoni
Terrathelphusa_kuhli
ndathelphusa_minahassae
lthuisana_festiva
lthuisana_biroi
elvinkia_holthuisi
strothelphusa_sp
strothelphusa_transversa
Thelphusula_tawauensis
Thelphusula_hulu
Thelphusula_sabana
Thelphusula_batamensis
Arachnothelphusa_radamanthysi
Parathelphusa_maindroni_2
Parathelphusa_batamensis
Parathelphusa_convexa_1
Parathelphusa_sp_6_2
Parathelphusa_reticulata
Parathelphusa_maculata_1
rathelphusa_sp_5_2
Siamthelphusa_sp
Siamthelphusa_improvisa
Heterothelphusa_fatum
Sayamia_sexpunctata
Sayamia_bangkokensis
Salangathelphusa_brevicarinata
undathelphusa_boex
undathelphusa_cavernicola
undathelphusa_picta
ndleria_gloriosa
rrothelphusa_asserpes
undathelphusa_hades
Sundathelphusa_tenebrosa
Oziothelphusa_sp_India
Oziothelphusa_ceylonensis
Ceylonthelphusa_kandambyi
Barytelphusa_cunicularis
Barytelphusa_sp
Barytelphusa_cunicularis_ZRC20
Maydelliathelphusa_edentula
Maydelliathelphusa_lugubris
Sartoriana_spinigera
Sodhiana_blanfordi
Gecarcinucus_jacquemonti_2
Gecarcinucus_jacquemonti
Lamella_lamellifrons_ZRC2003_0
Pilarta_anuka_ZRC2003_0238_SG7
Gubernatoriana_basalticola_SG4
Gubernatoriana_basalticola_SG4
Gubernatoriana_gubernatoris_AY
Travancoriana_charu_ZRC2003_02
Travancoriana_pollicaris_S8_9
Baratha_pushta_ZRC2003_0234_SG
Baratha_peena_ZRC2003_0235_SG7
Vela_virupa_ZRC2003_0236_SG7_1
Vanni_malabarica_S8_18
Vanni_travancoriana_ZRC2003_02
Vanni_deepta_ZRC2003_0233_SG7_
Cylindrotelphusa_steniops
Cylindrotelphusa_sp_SMF2754
Snaha_escheri_S8_15
Phricotelphusa_gracilipes
Phricotelphusa_hockpingi
Phricotelphusa_amnicola
Phricotelphusa_limula
Phricotelphusa_sirindhorn
Thaksinthelphusa_yongchindarat
Liotelphusa_gagei
Lepidothelphusa_cognetti
Vanni_nilgiriensis_NHMB802a
Travancoriana_schirnerae_AY
Travancoriana_schirnerae
40
30
20
10
0
Millions of years ago

Gecarcinucidae BioGeoBEARS DEC+J
ancstates: global optim, 2 areas max. d=0; e=0; j=0.0187; LnL=−38.72

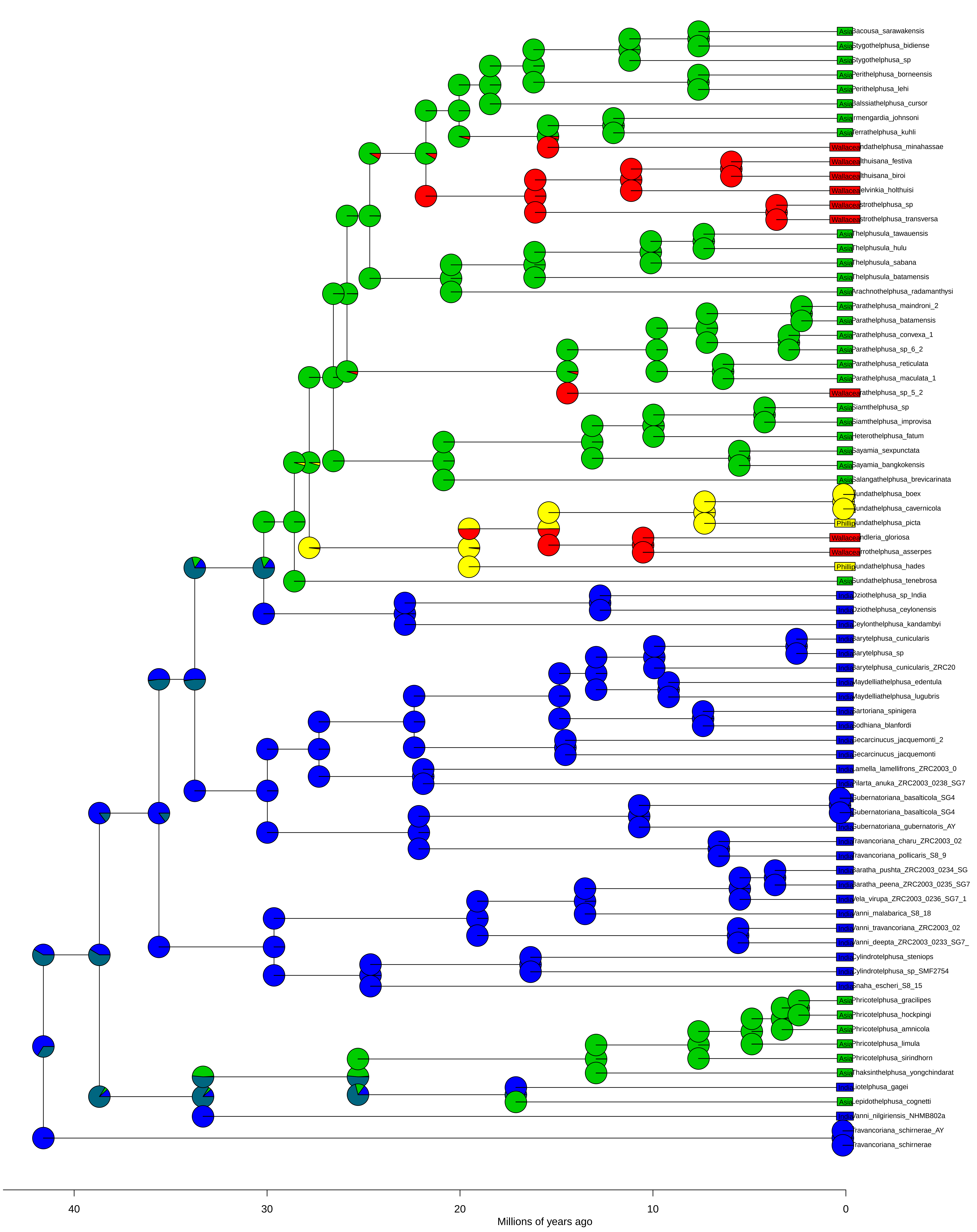

Papilionidae BioGeoBEARS DEC+J

ancstates: global optim, 4 areas max. d=0.0029; e=0; j=0.0105; LnL=−384.97

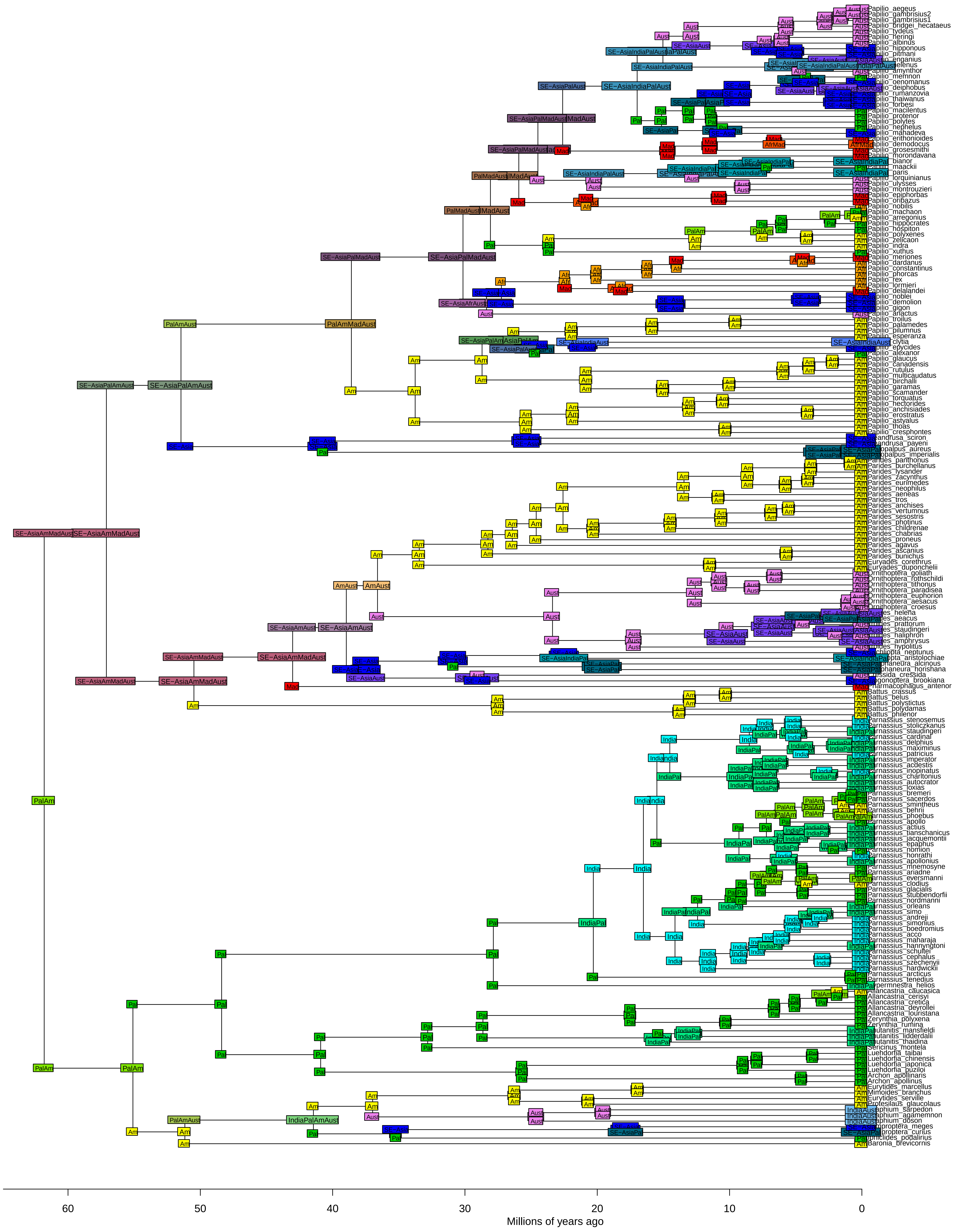

Papilionidae BioGeoBEARS DEC+J
ancstates: global optim, 4 areas max. d=0.0029; e=0; j=0.0105; LnL=−384.97

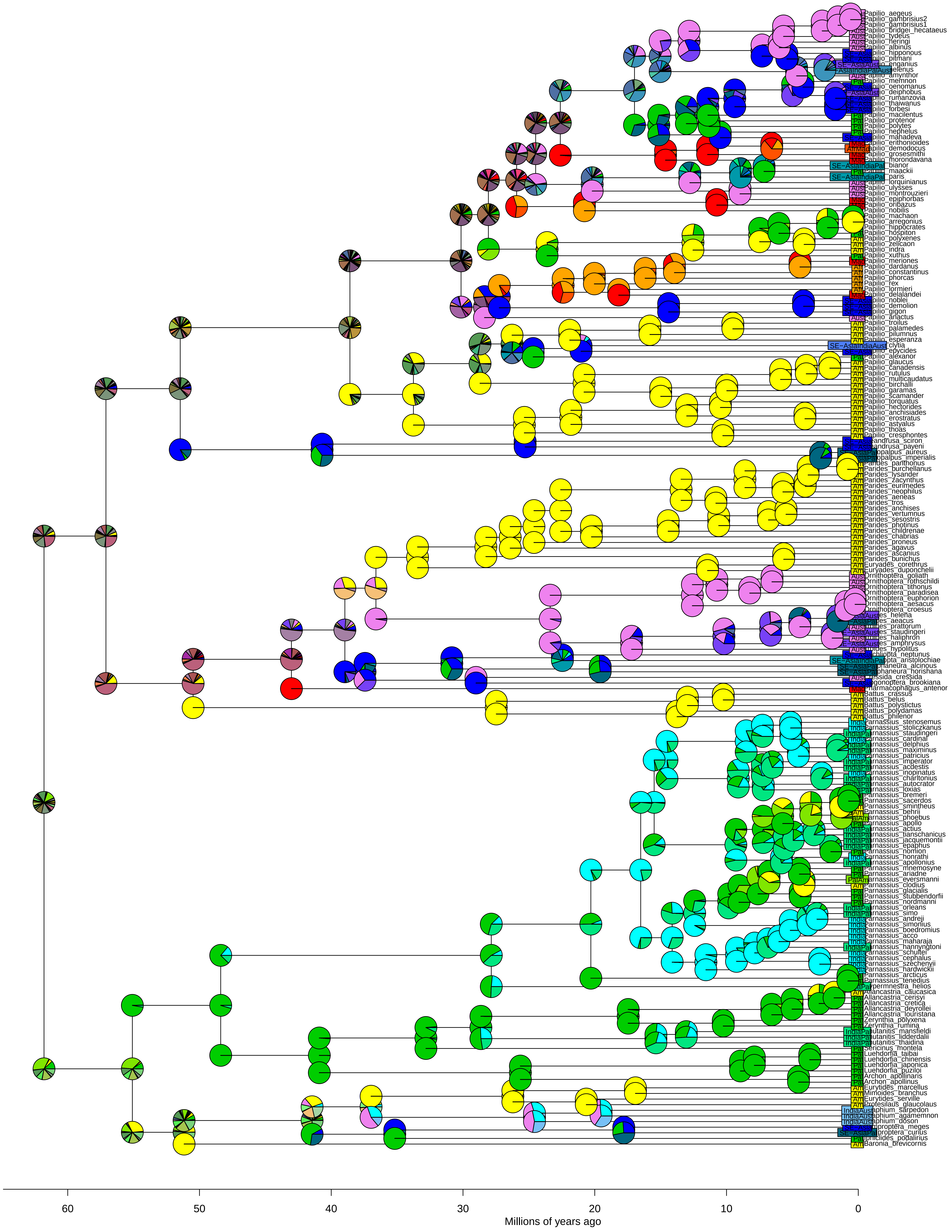

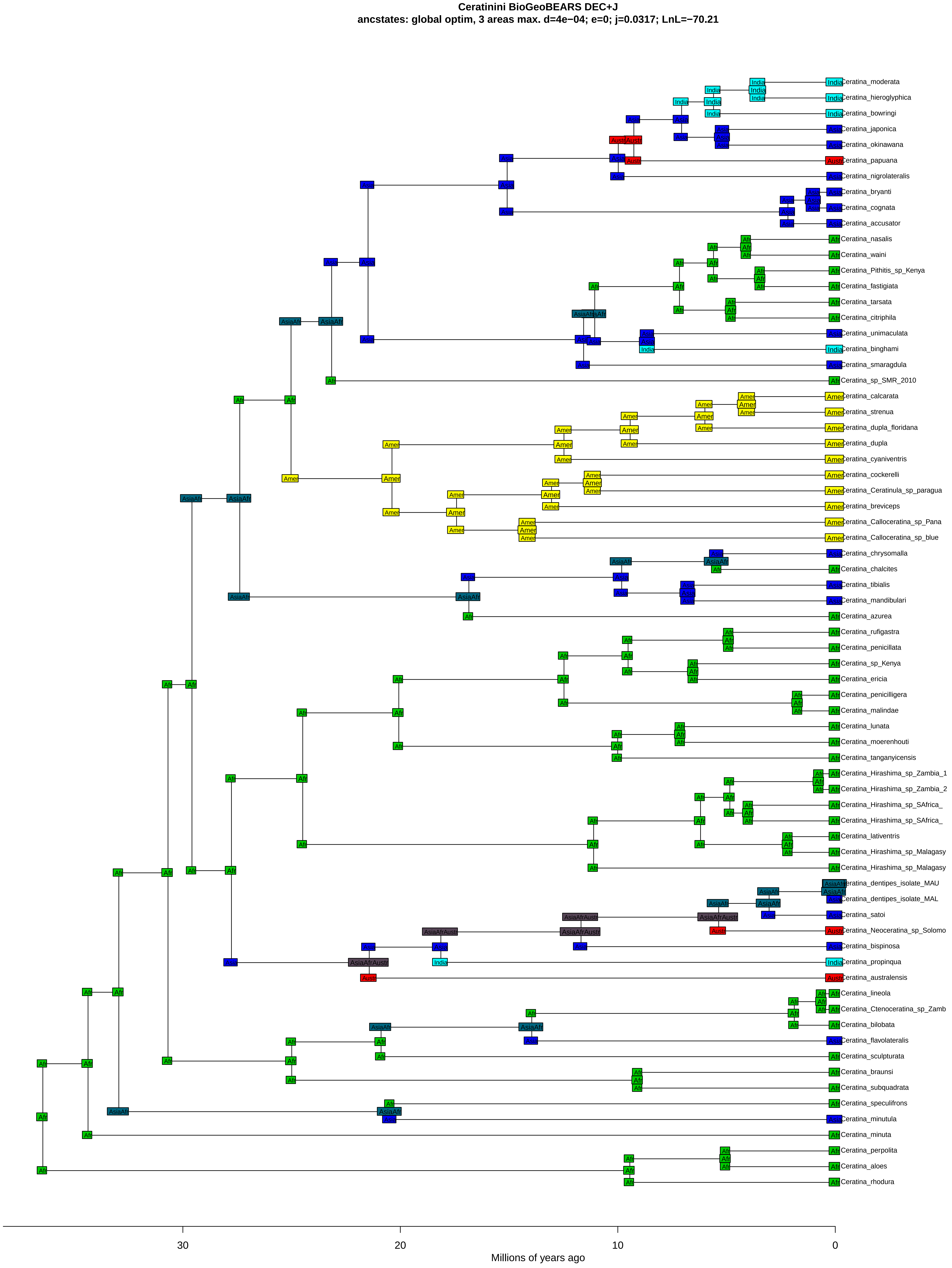
Ceratinini BioGeoBEARS DEC+J
ancstates: global optim, 3 areas max. d=4e−04; e=0; j=0.0317; LnL=−70.21
Ceratina_moderata
Ceratina_hieroglyphica
Ceratina_bowringi
Ceratina_japonica
Ceratina_okinawana
Ceratina_papuana
Ceratina_nigrolateralis
Ceratina_bryanti
Ceratina_cognata
Ceratina_accusator
Ceratina_nasalis
Ceratina_waini
Ceratina_Pithitis_sp_Kenya
Ceratina_fastigiata
Ceratina_tarsata
Ceratina_citriphila
Ceratina_unimaculata
Ceratina_binghami
Ceratina_smaragdula
Ceratina_sp_SMR_2010
Ceratina_calcarata
Ceratina_strenua
Ceratina_dupla_floridana
Ceratina_dupla
Ceratina_cyaniventris
Ceratina_cockerelli
Ceratina_Ceratinula_sp_paragua
Ceratina_breviceps
Ceratina_Calloceratina_sp_Pana
Ceratina_Calloceratina_sp_blue
Ceratina_chrysomalla
Ceratina_chalcites
Ceratina_tibialis
Ceratina_mandibulari
Ceratina_azurea
Ceratina_rufigastra
Ceratina_penicillata
Ceratina_sp_Kenya
Ceratina_ericia
Ceratina_penicilligera
Ceratina_malindae
Ceratina_lunata
Ceratina_moerenhouti
Ceratina_tanganyicensis
Ceratina_Hirashima_sp_Zambia_1
Ceratina_Hirashima_sp_Zambia_2
Ceratina_Hirashima_sp_SAfrica_
Ceratina_Hirashima_sp_SAfrica_
Ceratina_lativentris
Ceratina_Hirashima_sp_Malagasy
Ceratina_Hirashima_sp_Malagasy
Ceratina_dentipes_isolate_MAU
Ceratina_dentipes_isolate_MAL
Ceratina_satoi
Ceratina_Neoceratina_sp_Solomo
Ceratina_bispinosa
Ceratina_propinqua
Ceratina_australensis
Ceratina_lineola
Ceratina_Ctenoceratina_sp_Zamb
Ceratina_bilobata
Ceratina_flavolateralis
Ceratina_sculpturata
Ceratina_braunsi
Ceratina_subquadrata
Ceratina_speculifrons
Ceratina_minutula
Ceratina_minuta
Ceratina_perpolita
Ceratina_aloes
Ceratina_rhodura
30
20
10
0
Millions of years ago

Ceratinini BioGeoBEARS DEC+J
ancstates: global optim, 3 areas max. d=4e−04; e=0; j=0.0317; LnL=−70.21

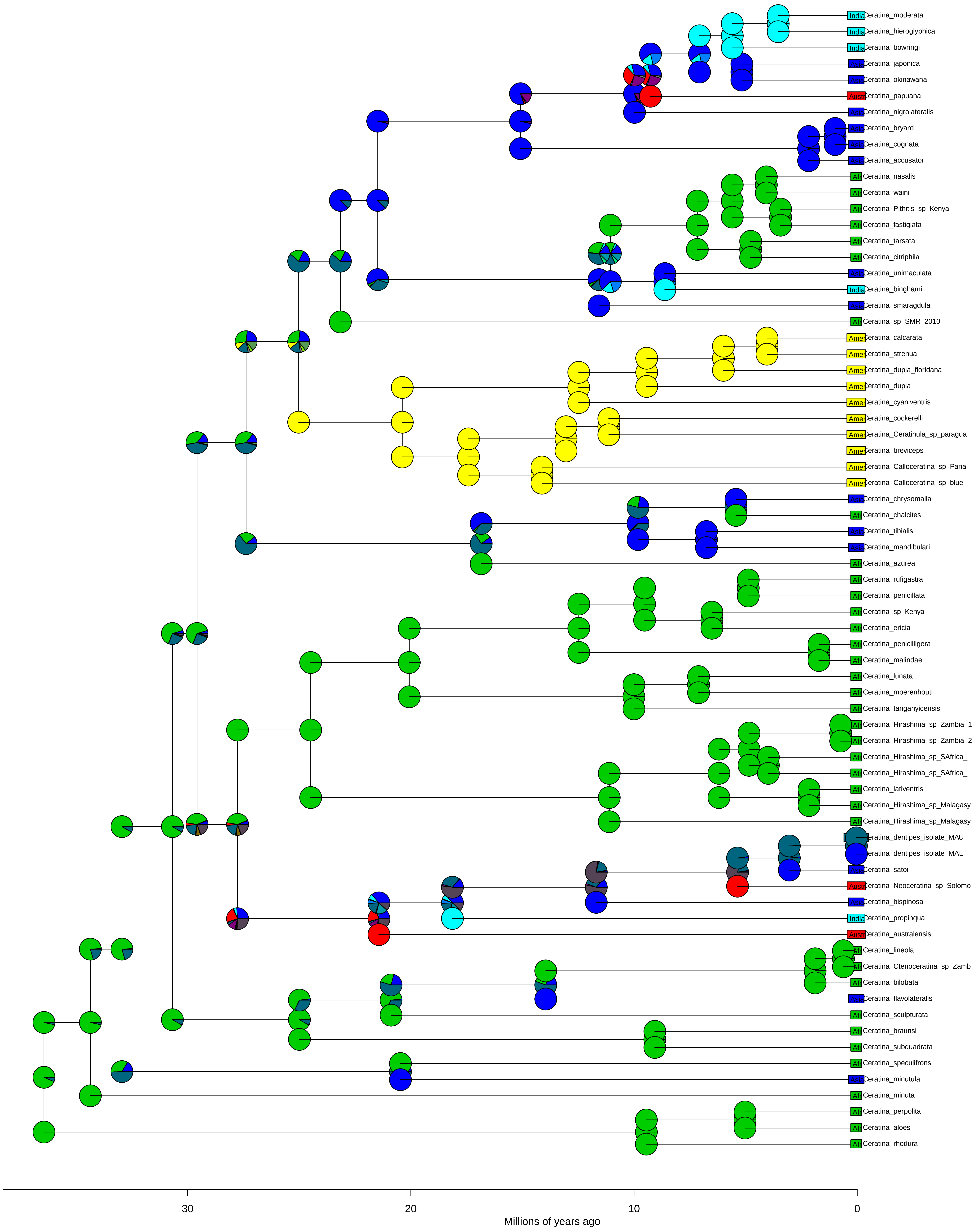

Pseudomyrmecinae BioGeoBEARS DEC+J
ancstates: global optim, 2 areas max. d=0; e=0; j=0.0224; LnL=−26.62

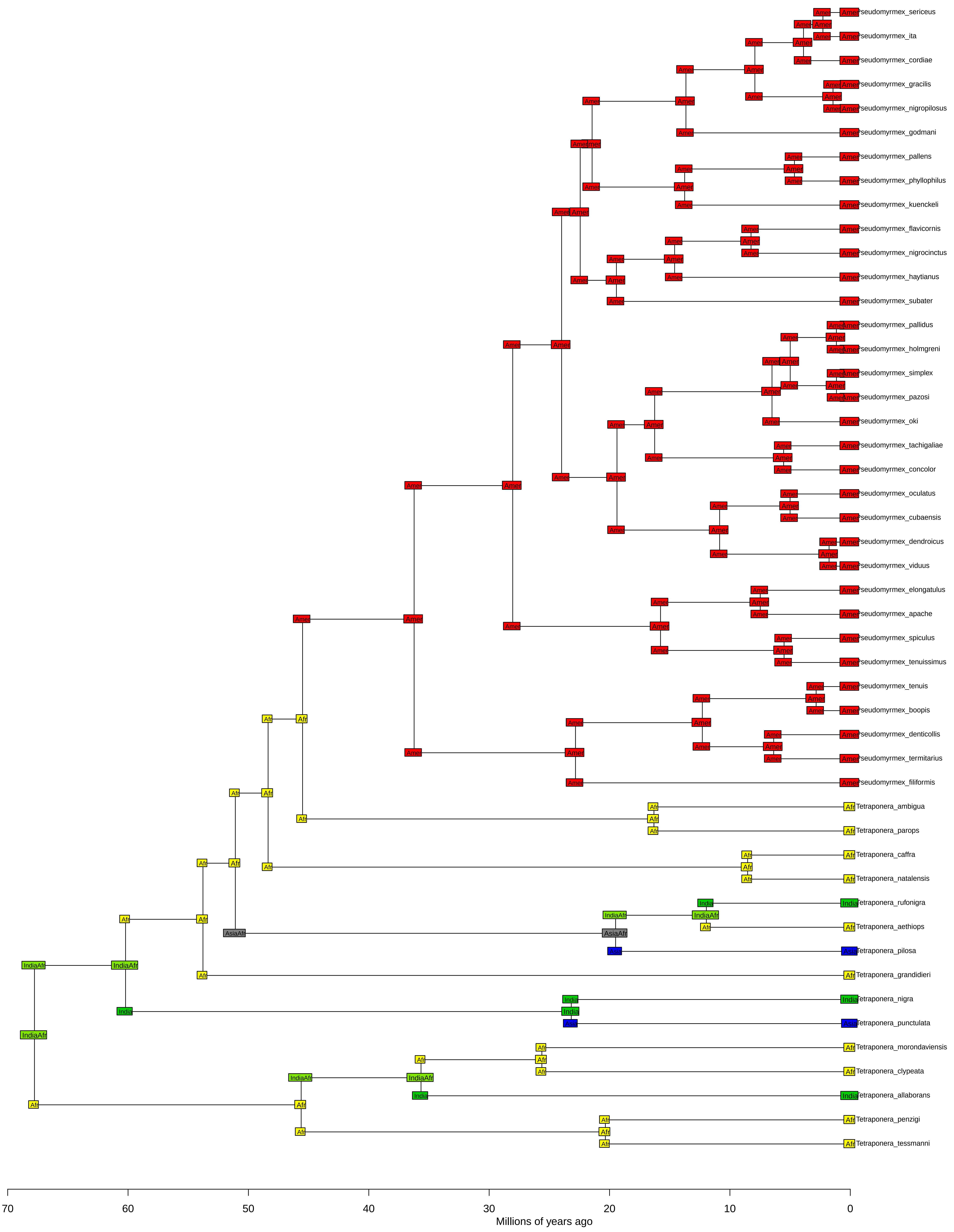

Pseudomyrmecinae BioGeoBEARS DEC+J
ancstates: global optim, 2 areas max. d=0; e=0; j=0.0224; LnL=−26.62

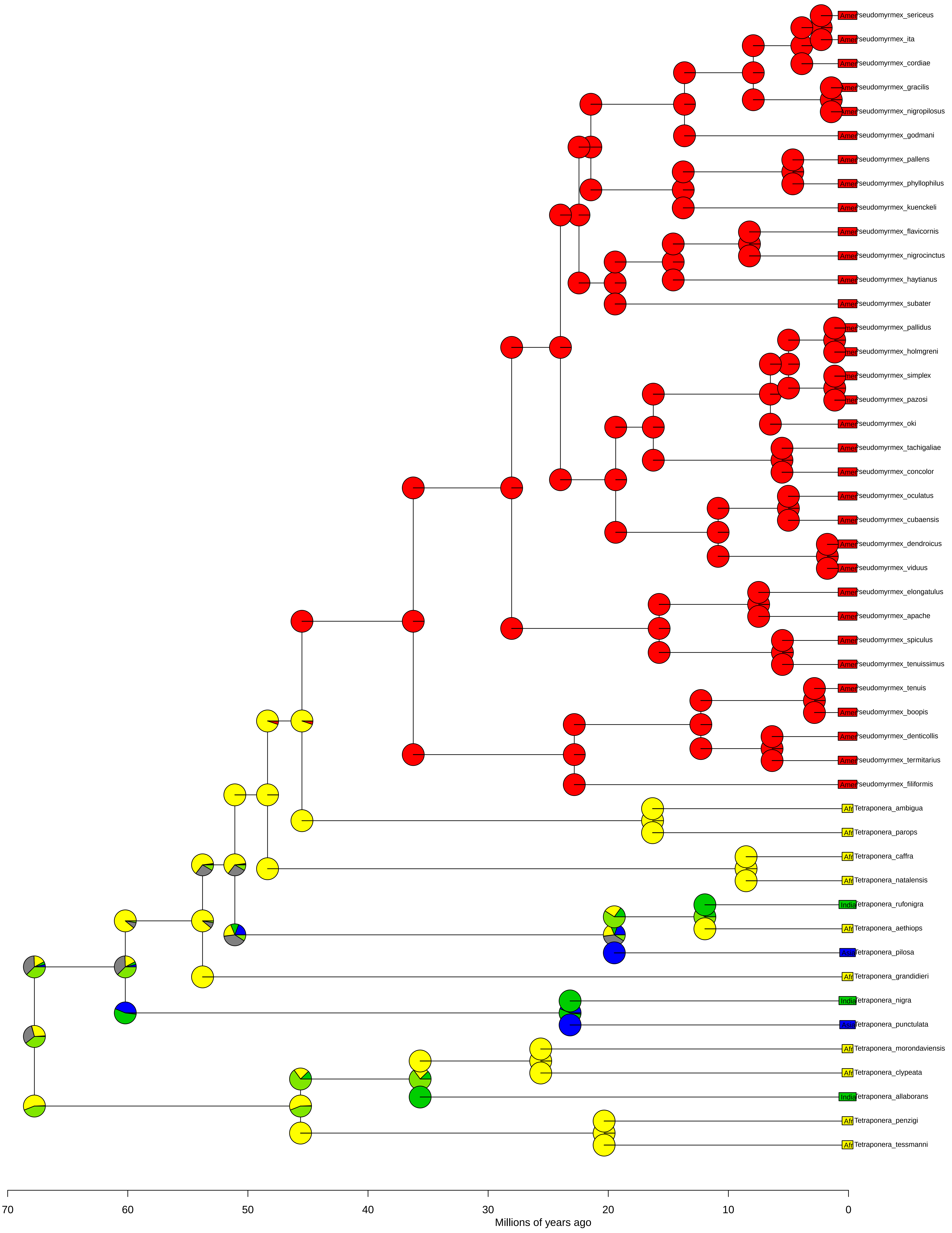

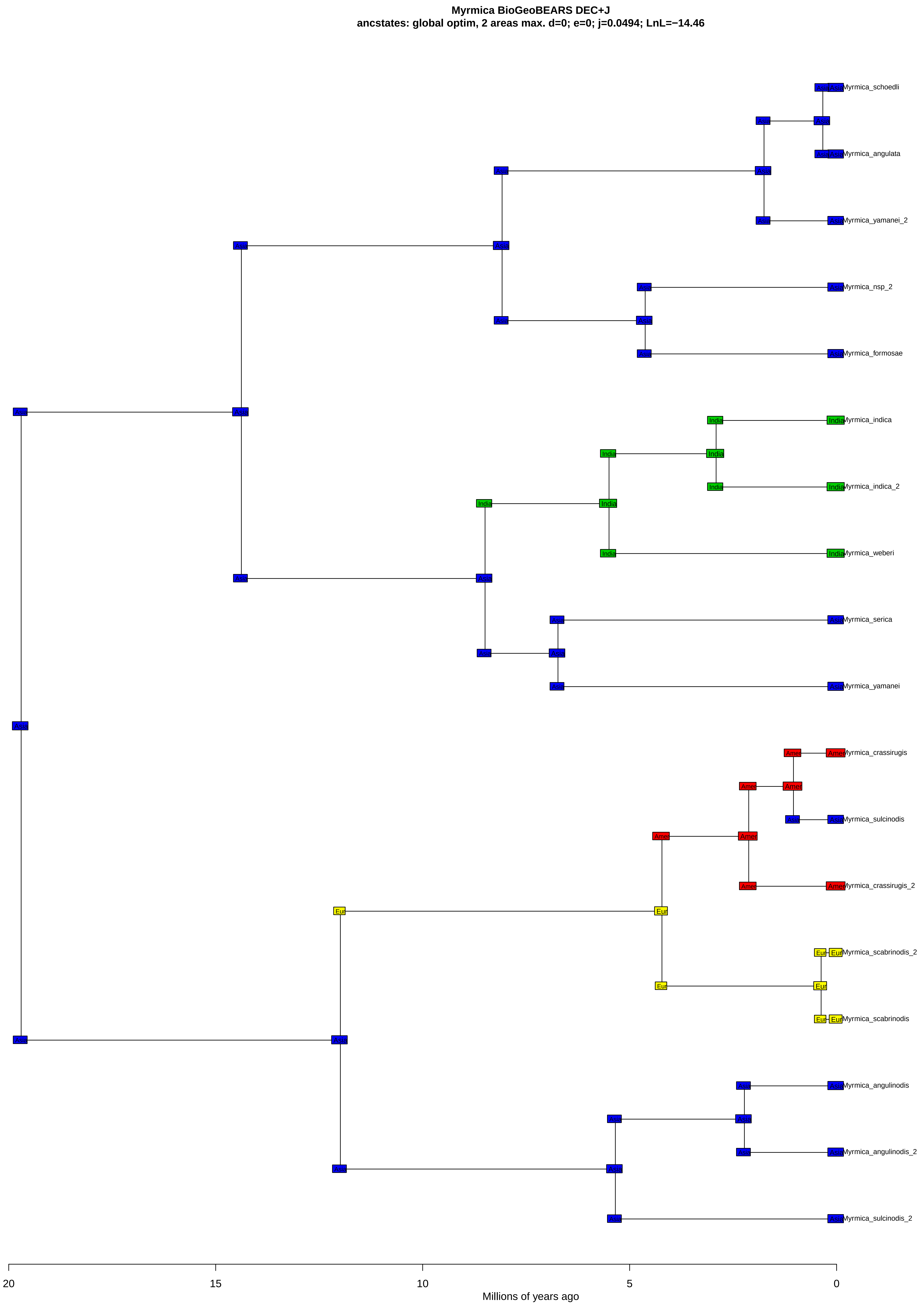
Myrmica BioGeoBEARS DEC+J
ancstates: global optim, 2 areas max. d=0; e=0; j=0.0494; LnL=−14.46
Myrmica_schoedli
Myrmica_angulata
Myrmica_yamanei_2
Myrmica_nsp_2
Myrmica_formosae
Myrmica_indica
Myrmica_indica_2
Myrmica_weberi
Myrmica_serica
Myrmica_yamanei
Myrmica_crassirugis
Myrmica_sulcinodis
Myrmica_crassirugis_2
Myrmica_scabrinodis_2
Myrmica_scabrinodis
Myrmica_angulinodis
Myrmica_angulinodis_2
Myrmica_sulcinodis_2
India
Amer
Eur
20
15
10
5
0
Millions of years ago

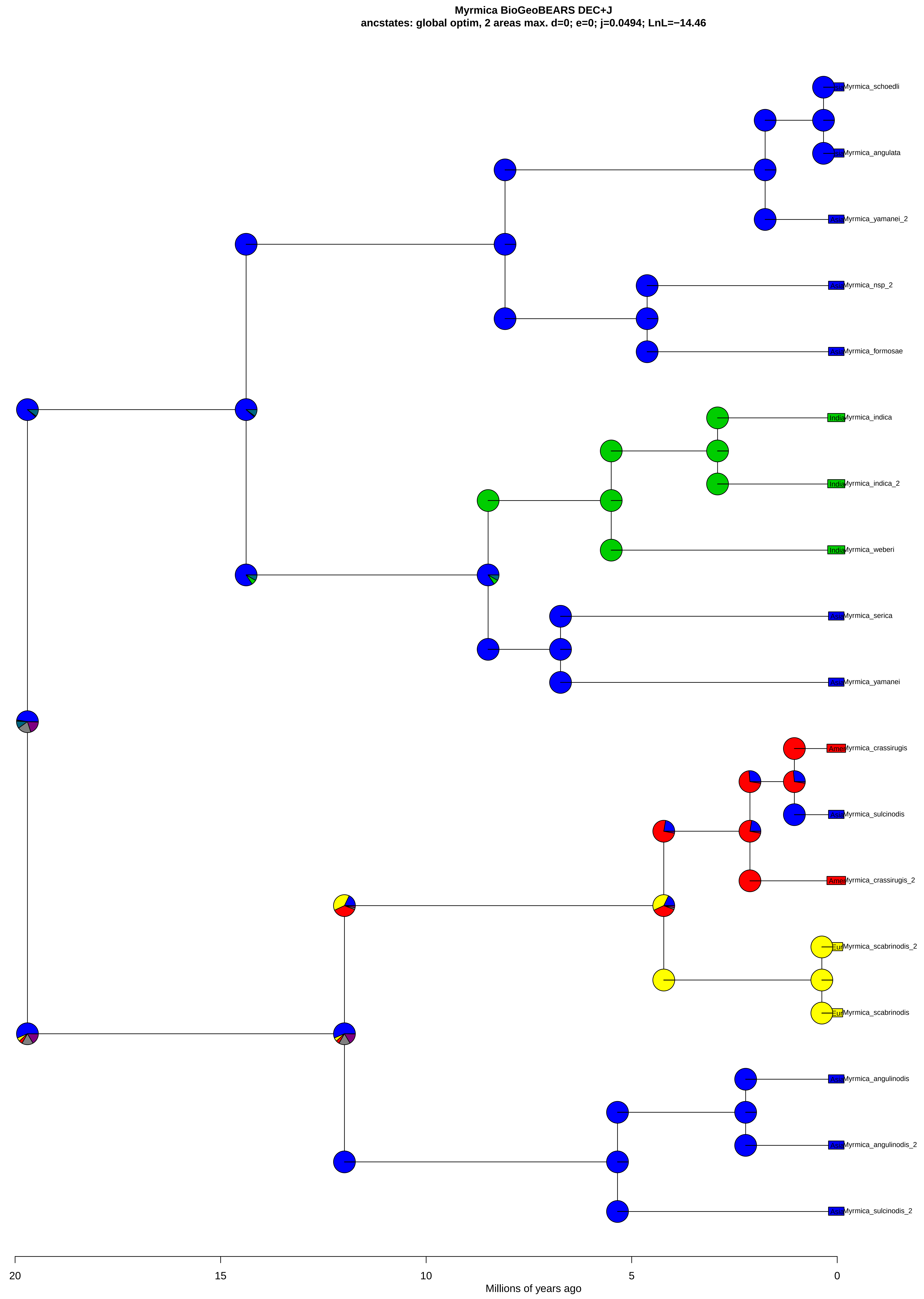
Myrmica BioGeoBEARS DEC+J
ancstates: global optim, 2 areas max. d=0; e=0; j=0.0494; LnL=−14.46
Myrmica_schoedli
Myrmica_angulata
Myrmica_yamanei_2
Myrmica_nsp_2
Myrmica_formosae
India
Myrmica_indica
India
Myrmica_indica_2
India
Myrmica_weberi
Myrmica_serica
Myrmica_yamanei
Amer
Myrmica_crassirugis
Myrmica_sulcinodis
Amer
Myrmica_crassirugis_2
Eur
Myrmica_scabrinodis_2
Eur
Myrmica_scabrinodis
Myrmica_angulinodis
Myrmica_angulinodis_2
Myrmica_sulcinodis_2
20
15
10
5
0
Millions of years ago

Aplocheiloidei BioGeoBEARS DEC+J
ancstates: global optim, 2 areas max. d=0; e=0; j=0.0259; LnL=−17.88

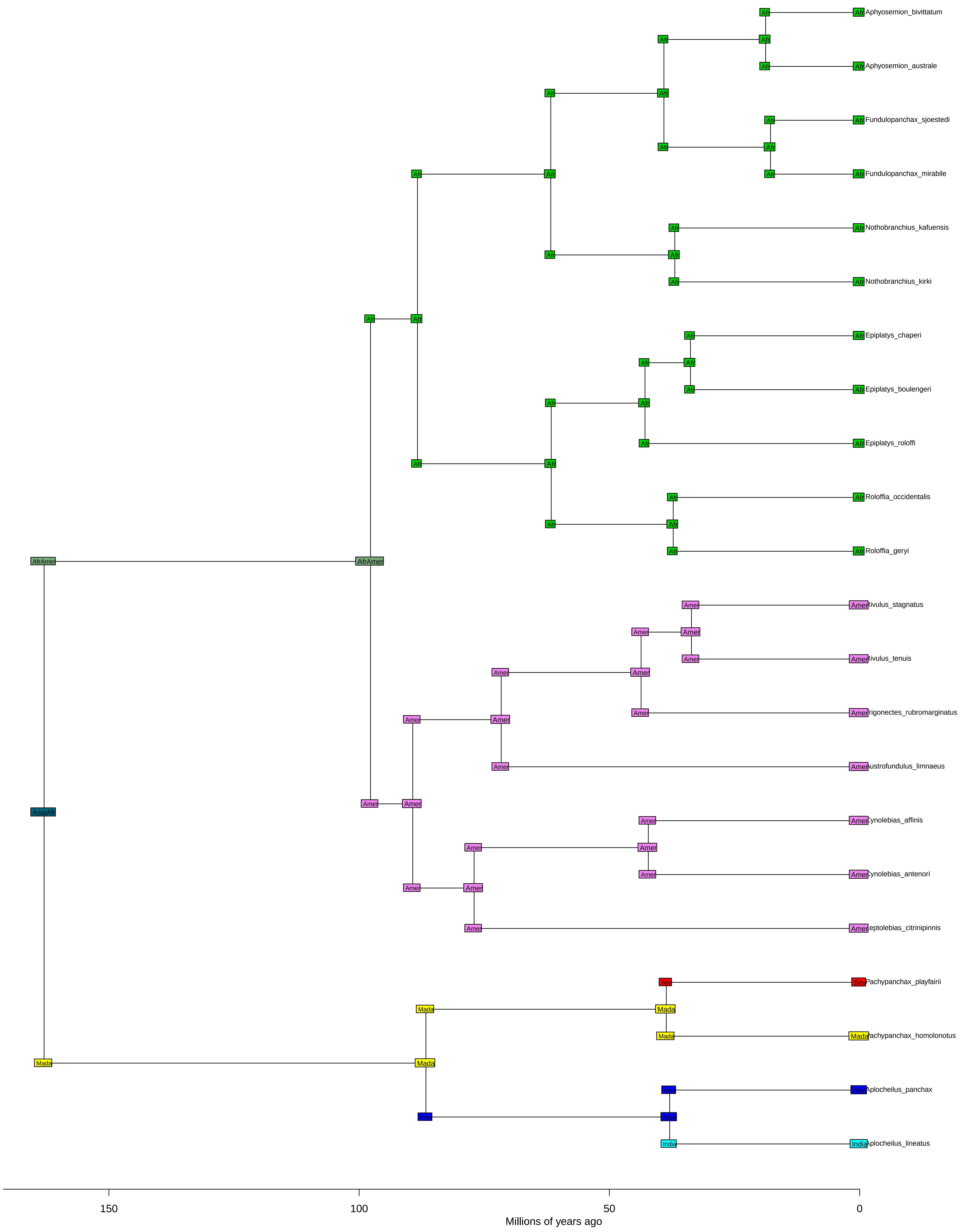

Aplocheiloidei BioGeoBEARS DEC+J
ancstates: global optim, 2 areas max. d=0; e=0; j=0.0259; LnL=−17.88

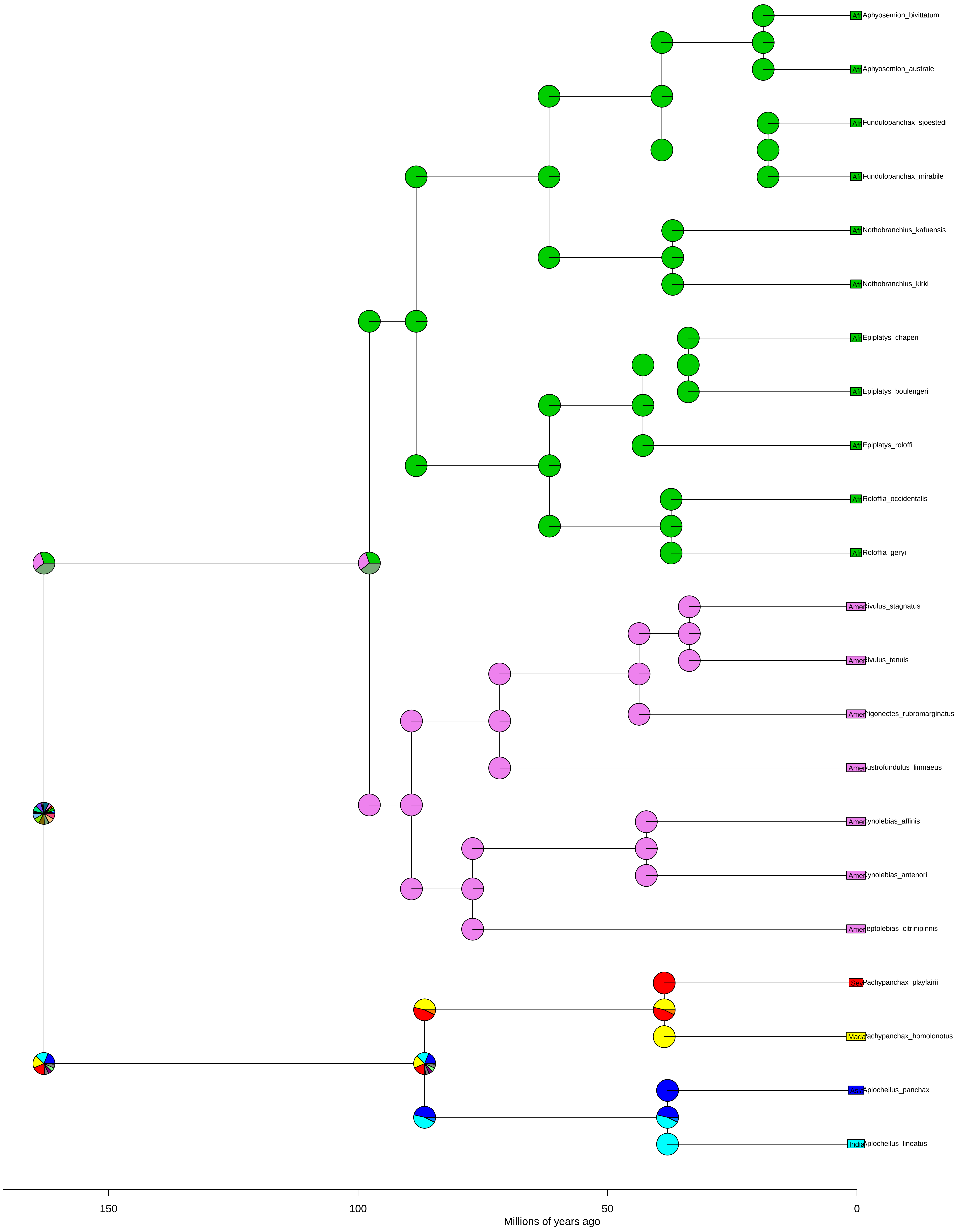

Channa BioGeoBEARS DEC+J
ancstates: global optim, 2 areas max. d=0; e=0; j=0.3815; LnL=−9.44

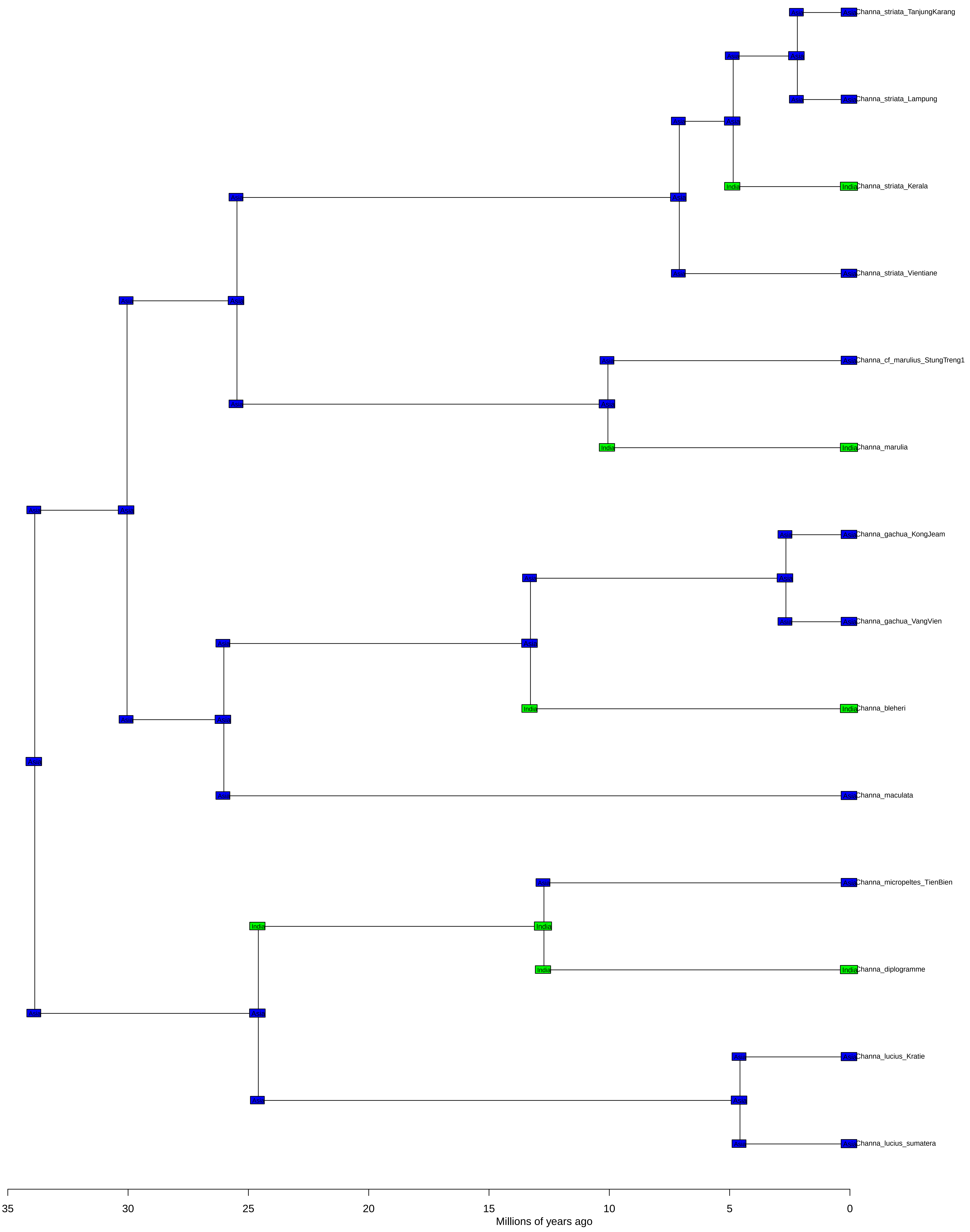

Channa BioGeoBEARS DEC+J
ancstates: global optim, 2 areas max. d=0; e=0; j=0.3815; LnL=−9.44

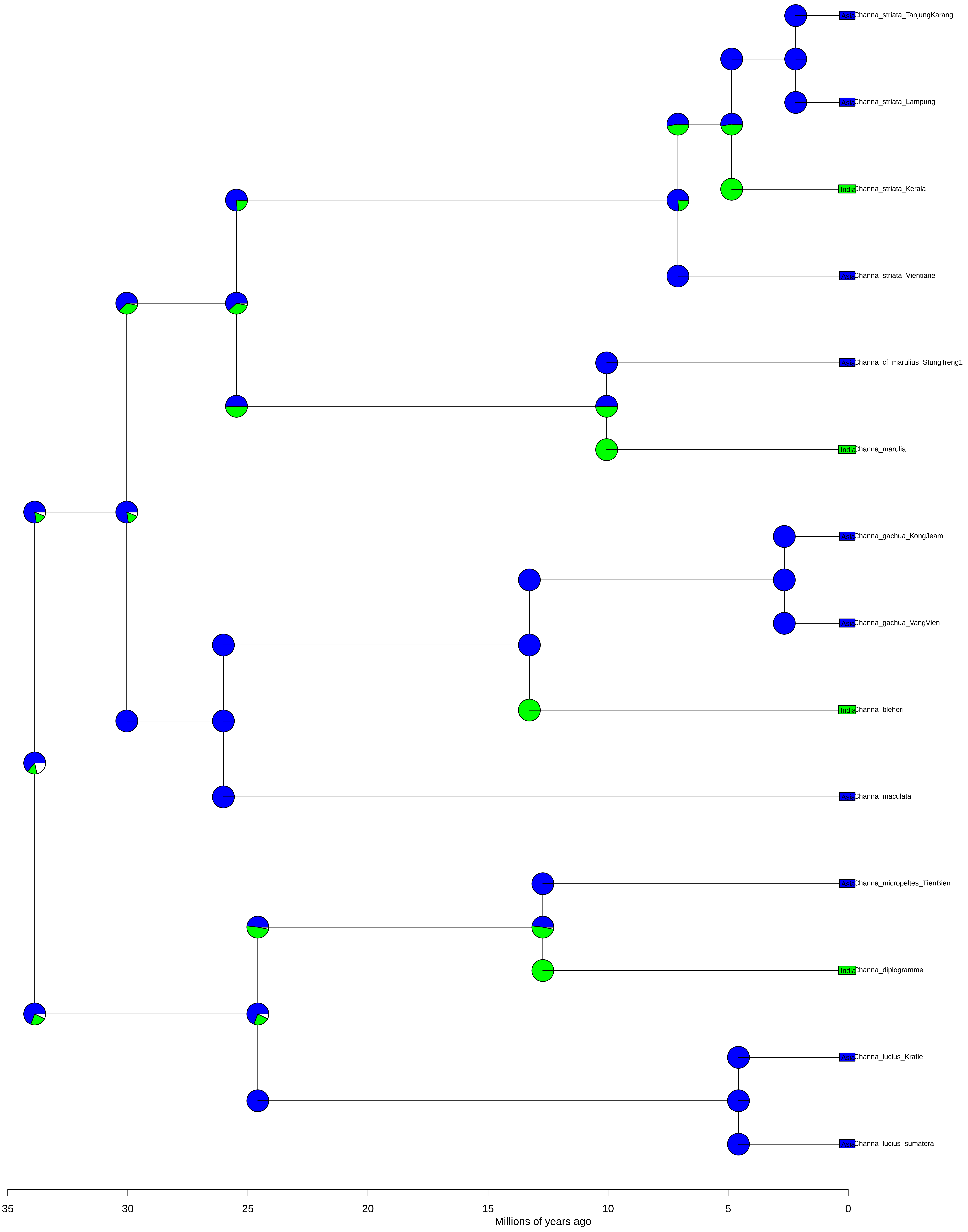

Anabantoidei, Osphronemidae BioGeoBEARS DEC+J
ancstates: global optim, 2 areas max. d=0.0019; e=0; j=0.0207; LnL=−35.48

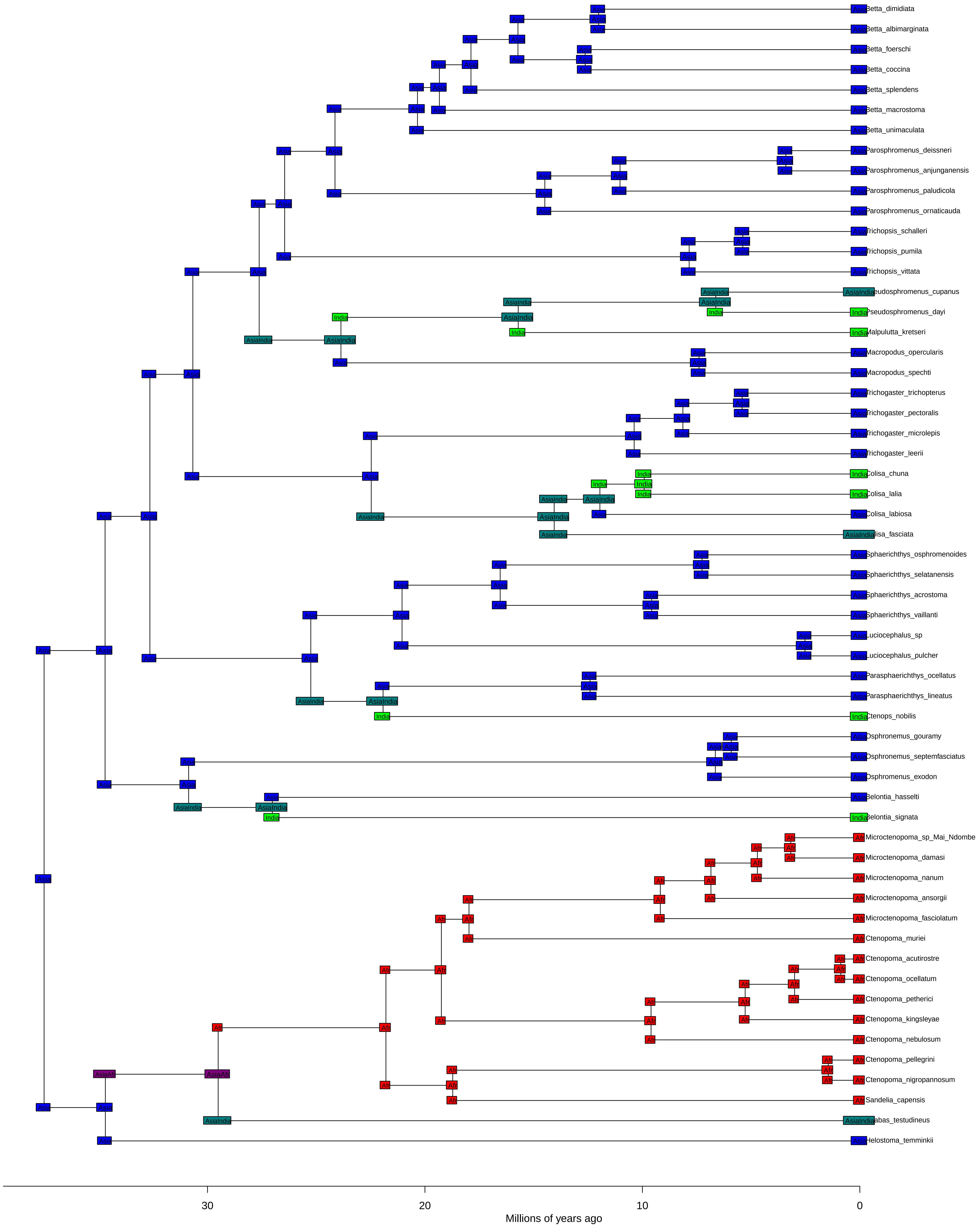

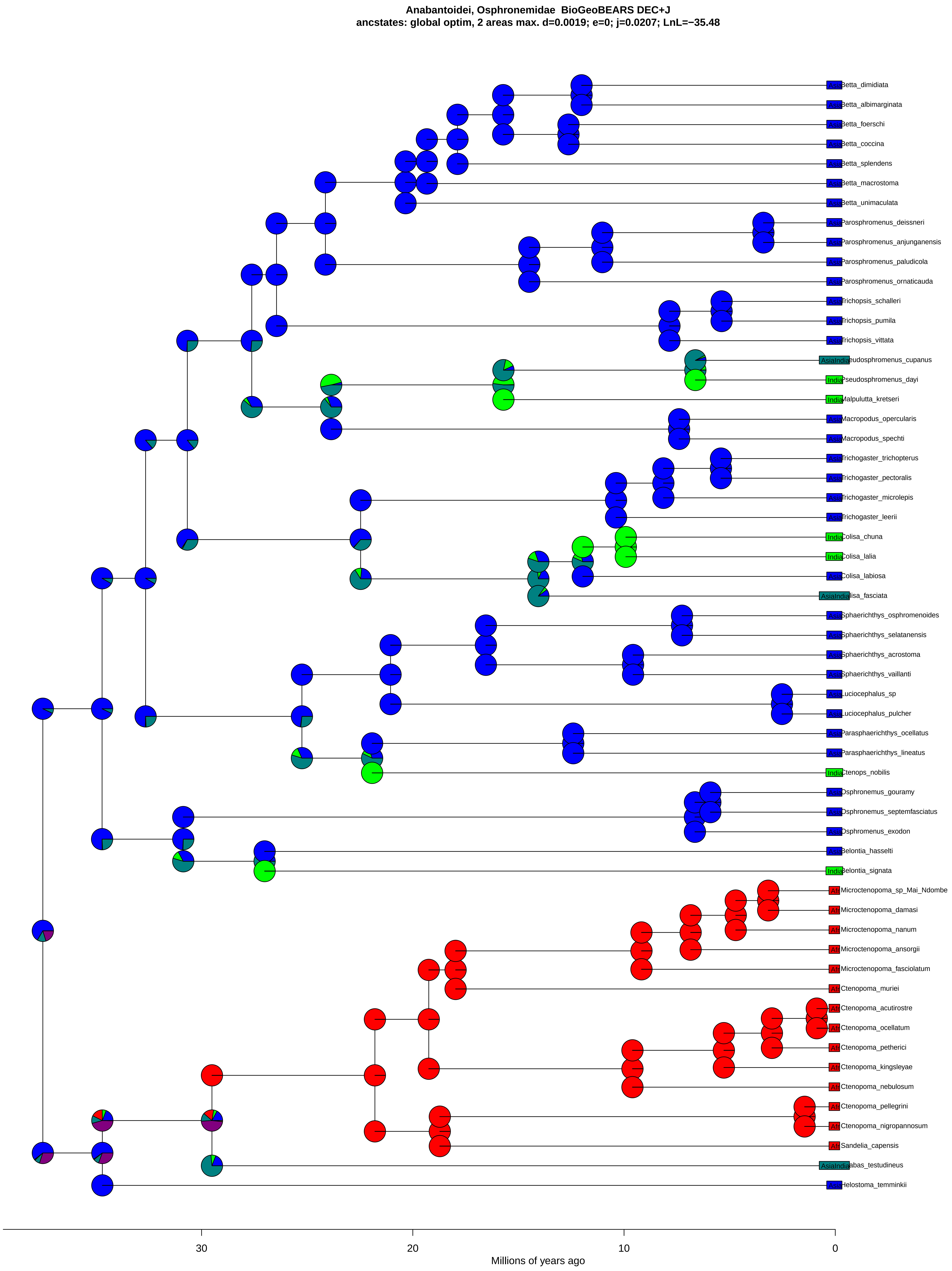

Anabantoidei, Osphronemidae BioGeoBEARS DEC+J
ancstates: global optim, 2 areas max. d=0.0019; e=0; j=0.0207; LnL=−35.48
Betta_dimidiata
Betta_albimarginata
Betta_foerschi
Betta_coccina
Betta_splendens
Betta_macrostoma
Betta_unimaculata
Parosphromenus_deissneri
Parosphromenus_anjunganensis
Parosphromenus_paludicola
Parosphromenus_ornaticauda
Trichopsis_schalleri
Trichopsis_pumila
Trichopsis_vittata
AsiaIndia
eudosphromenus_cupanus
India
Pseudosphromenus_dayi
India
Malpulutta_kretseri
Macropodus_opercularis
Macropodus_spechti
Trichogaster_trichopterus
Trichogaster_pectoralis
Trichogaster_microlepis
Trichogaster_leerii
India
Colisa_chuna
India
Colisa_lalia
Colisa_labiosa
AsiaIndia
lisa_fasciata
Sphaerichthys_osphromenoides
Sphaerichthys_selatanensis
Sphaerichthys_acrostoma
Sphaerichthys_vaillanti
Luciocephalus_sp
Luciocephalus_pulcher
Parasphaerichthys_ocellatus
Parasphaerichthys_lineatus
India
Ctenops_nobilis
Osphronemus_gouramy
Osphronemus_septemfasciatus
Osphronemus_exodon
Belontia_hasselti
India
Belontia_signata
Af
Microctenopoma_sp_Mai_Ndombe
Af
Microctenopoma_damasi
Af
Microctenopoma_nanum
Af
Microctenopoma_ansorgii
Af
Microctenopoma_fasciolatum
Af
Ctenopoma_muriei
Af
Ctenopoma_acutirostre
Af
Ctenopoma_ocellatum
Af
Ctenopoma_petherici
Af
Ctenopoma_kingsleyae
Af
Ctenopoma_nebulosum
Af
Ctenopoma_pellegrini
Af
Ctenopoma_nigropannosum
Af
Sandelia_capensis
AsiaIndia
abas_testudineus
Helostoma_temminkii
30
20
10
0
Millions of years ago

Heteropneustes fossilis BioGeoBEARS DEC+J
ancstates: global optim, 2 areas max. d=0; e=0; j=0.071; LnL=−2.96

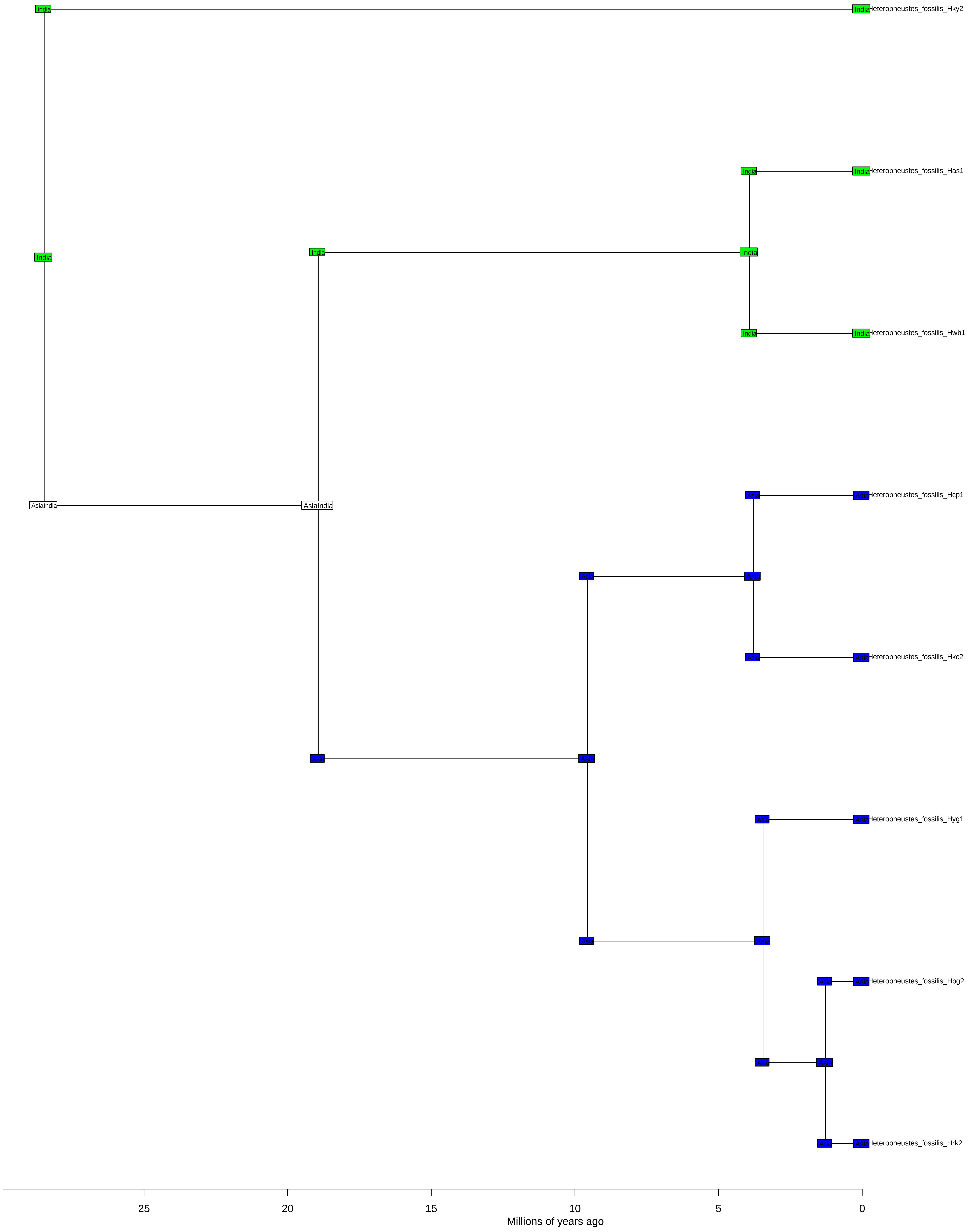

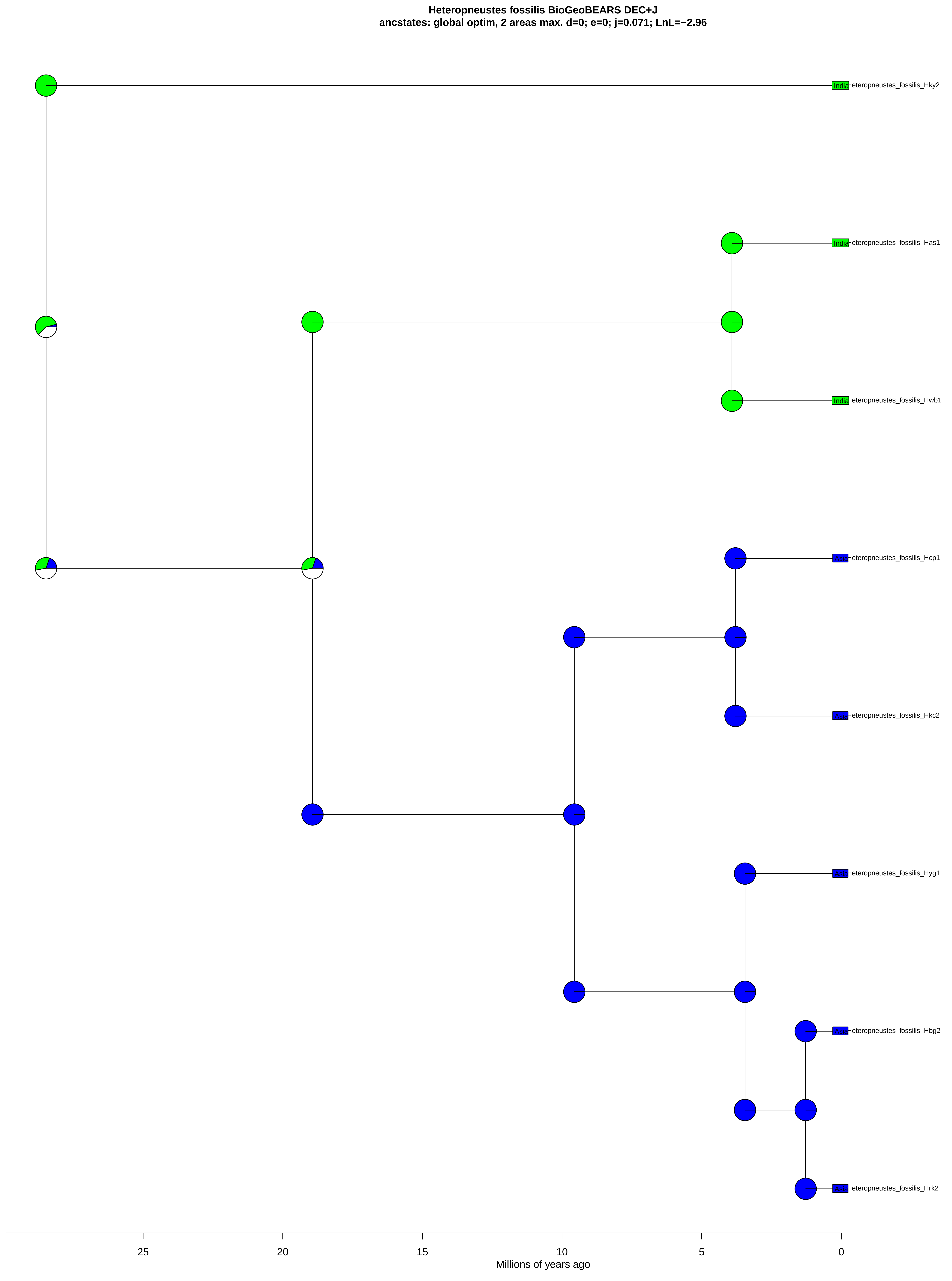
Heteropneustes fossilis BioGeoBEARS DEC+J
ancstates: global optim, 2 areas max. d=0; e=0; j=0.071; LnL=−2.96
India
Heteropneustes_fossilis_Hky2
India
Heteropneustes_fossilis_Has1
India
Heteropneustes_fossilis_Hwb1
Heteropneustes_fossilis_Hcp1
Heteropneustes_fossilis_Hkc2
Heteropneustes_fossilis_Hyg1
Heteropneustes_fossilis_Hbg2
Heteropneustes_fossilis_Hrk2
25
20
15
10
5
0
Millions of years ago

Cobitidae BioGeoBEARS DEC+J
ancstates: global optim, 2 areas max. d=0; e=0; j=0.0252; LnL=−14.02

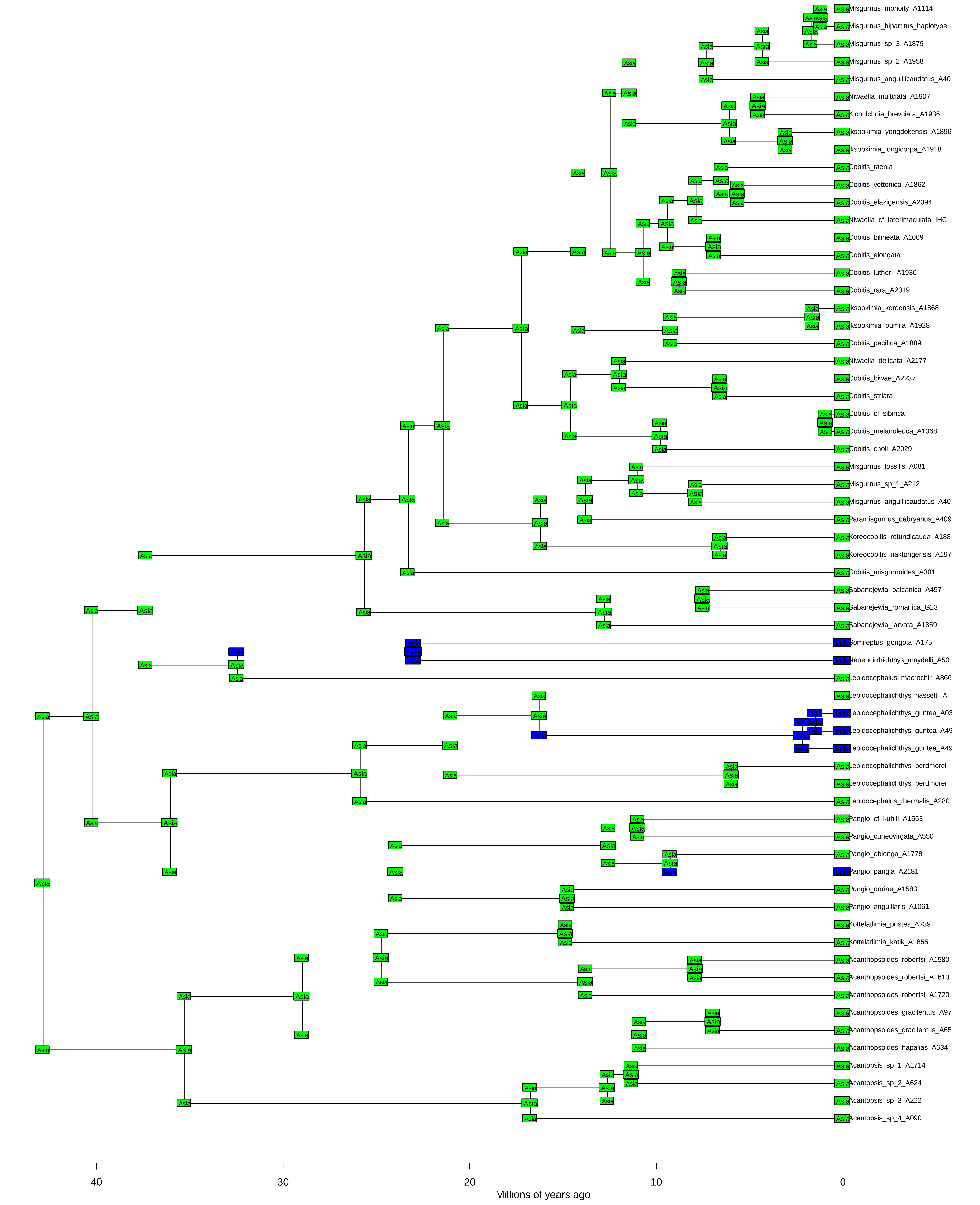

Cobitidae BioGeoBEARS DEC+J
ancstates: global optim, 2 areas max. d=0; e=0; j=0.0252; LnL=−14.02

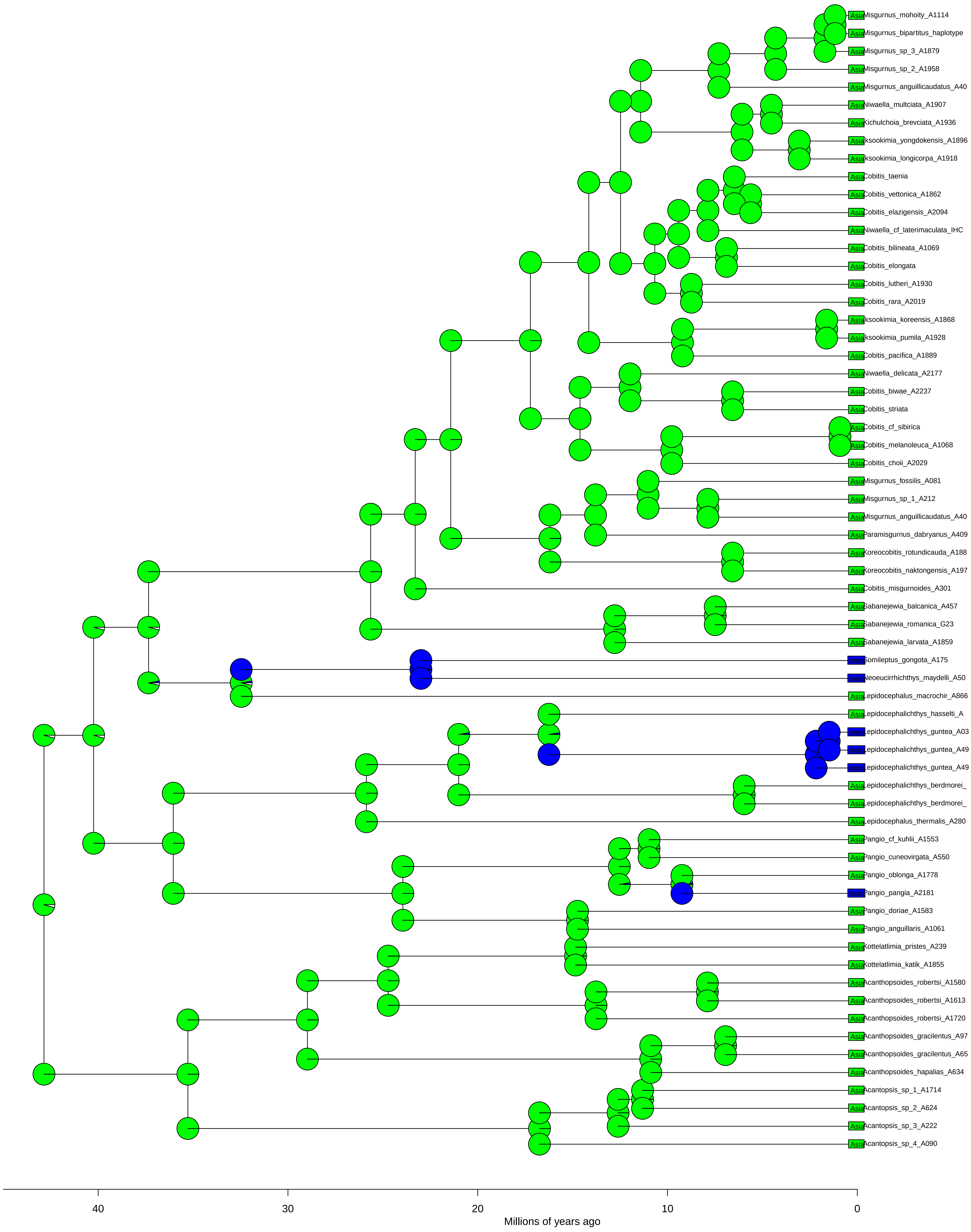

Microhylidae BioGeoBEARS DEC+J
ancstates: global optim, 2 areas max. d=0; e=0; j=0.0258; LnL=−4.32

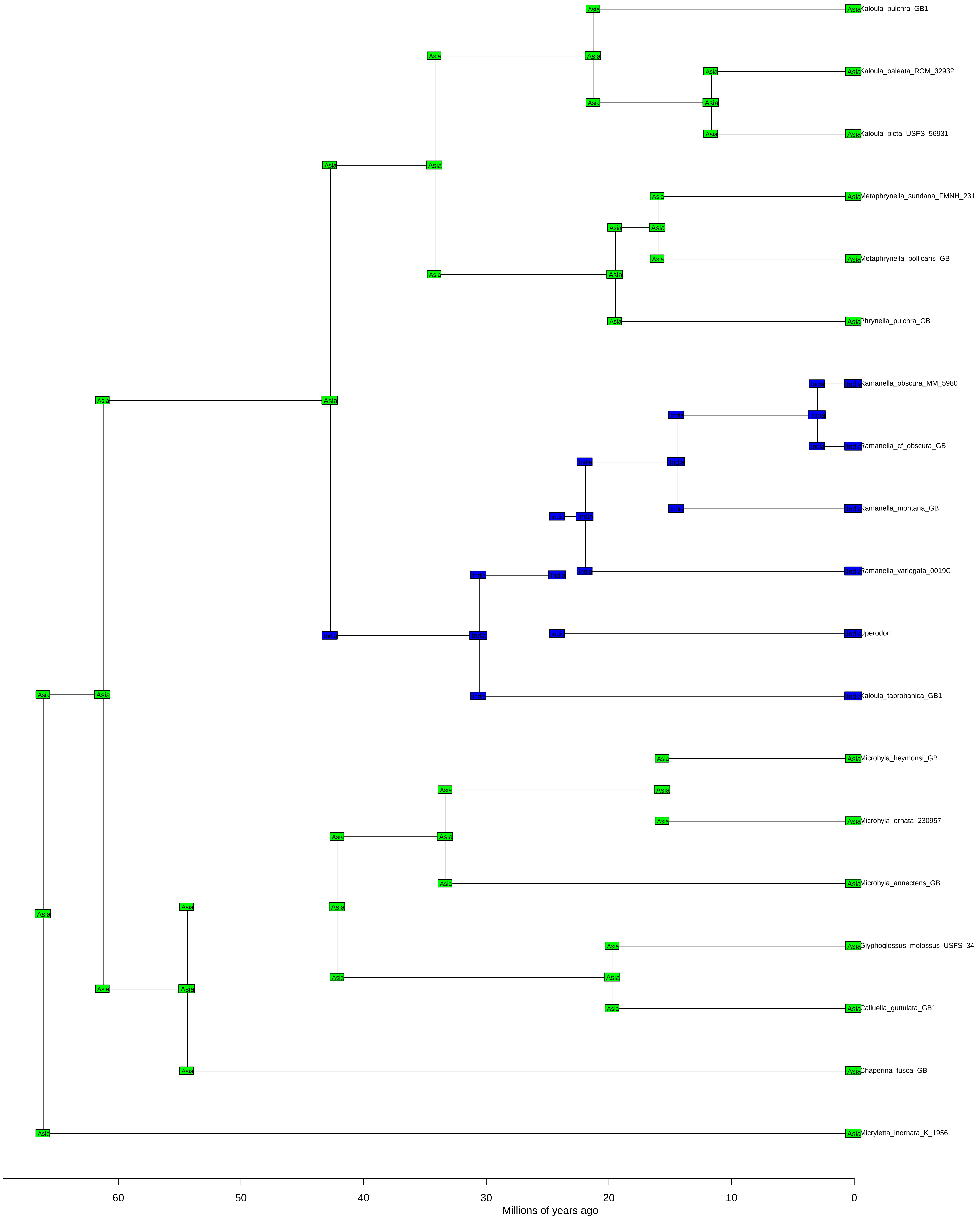

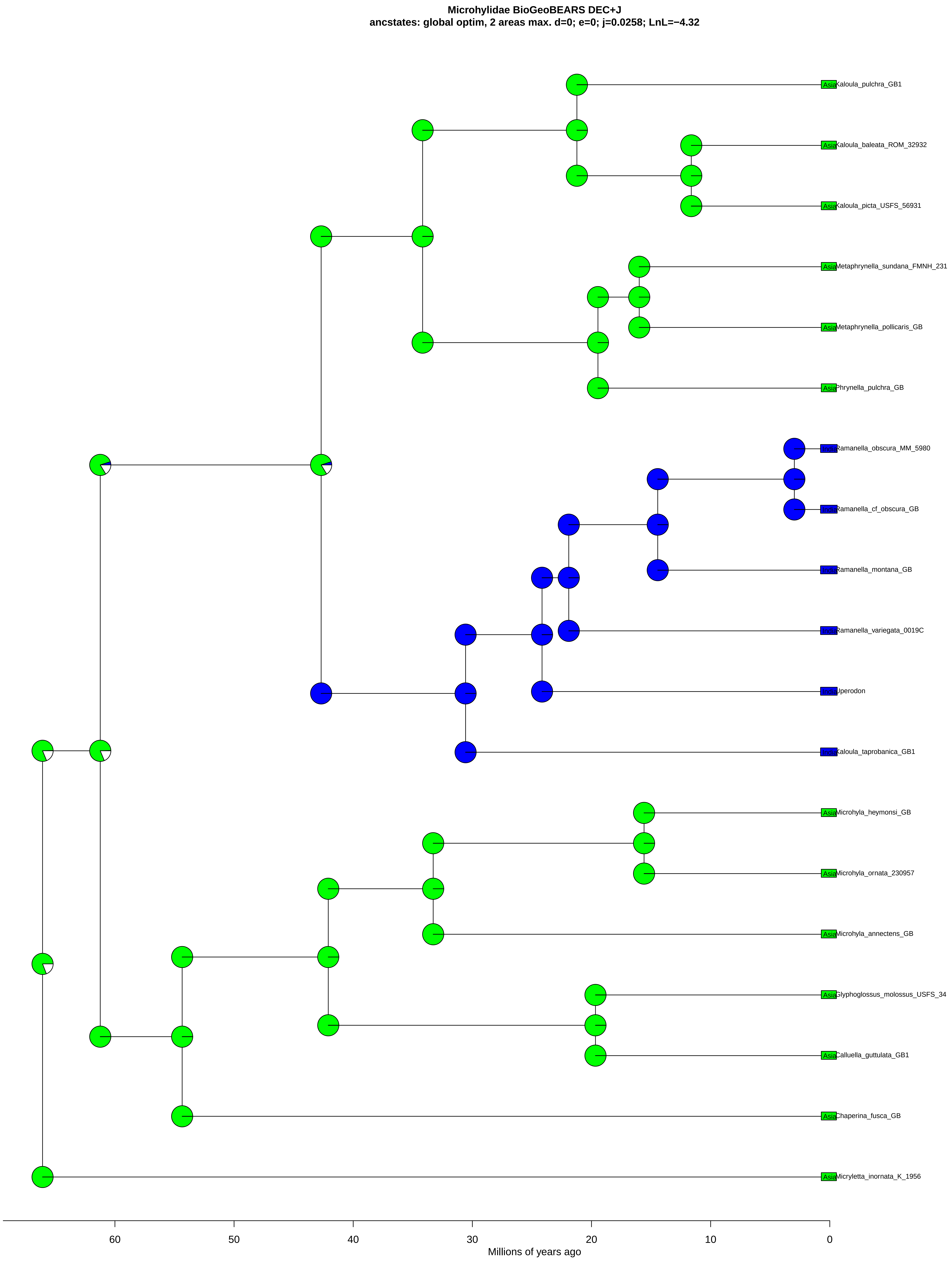
Microhylidae BioGeoBEARS DEC+J
ancstates: global optim, 2 areas max. d=0; e=0; j=0.0258; LnL=−4.32
Kaloula_pulchra_GB1
Kaloula_baleata_ROM_32932
Kaloula_picta_USFS_56931
Metaphrynella_sundana_FMNH_231
Metaphrynella_pollicaris_GB
Phrynella_pulchra_GB
Ramanella_obscura_MM_5980
Ramanella_cf_obscura_GB
Ramanella_montana_GB
Ramanella_variegata_0019C
Uperodon
Kaloula_taprobanica_GB1
Microhyla_heymonsi_GB
Microhyla_ornata_230957
Microhyla_annectens_GB
Glyphoglossus_molossus_USFS_34
Calluella_guttulata_GB1
Chaperina_fusca_GB
Micryletta_inornata_K_1956
Asia
60
50
40
30
20
10
0
Millions of years ago

Dicroglossidae BioGeoBEARS DEC+J
ancstates: global optim, 2 areas max. d=0; e=0; j=0.0324; LnL=−39.67

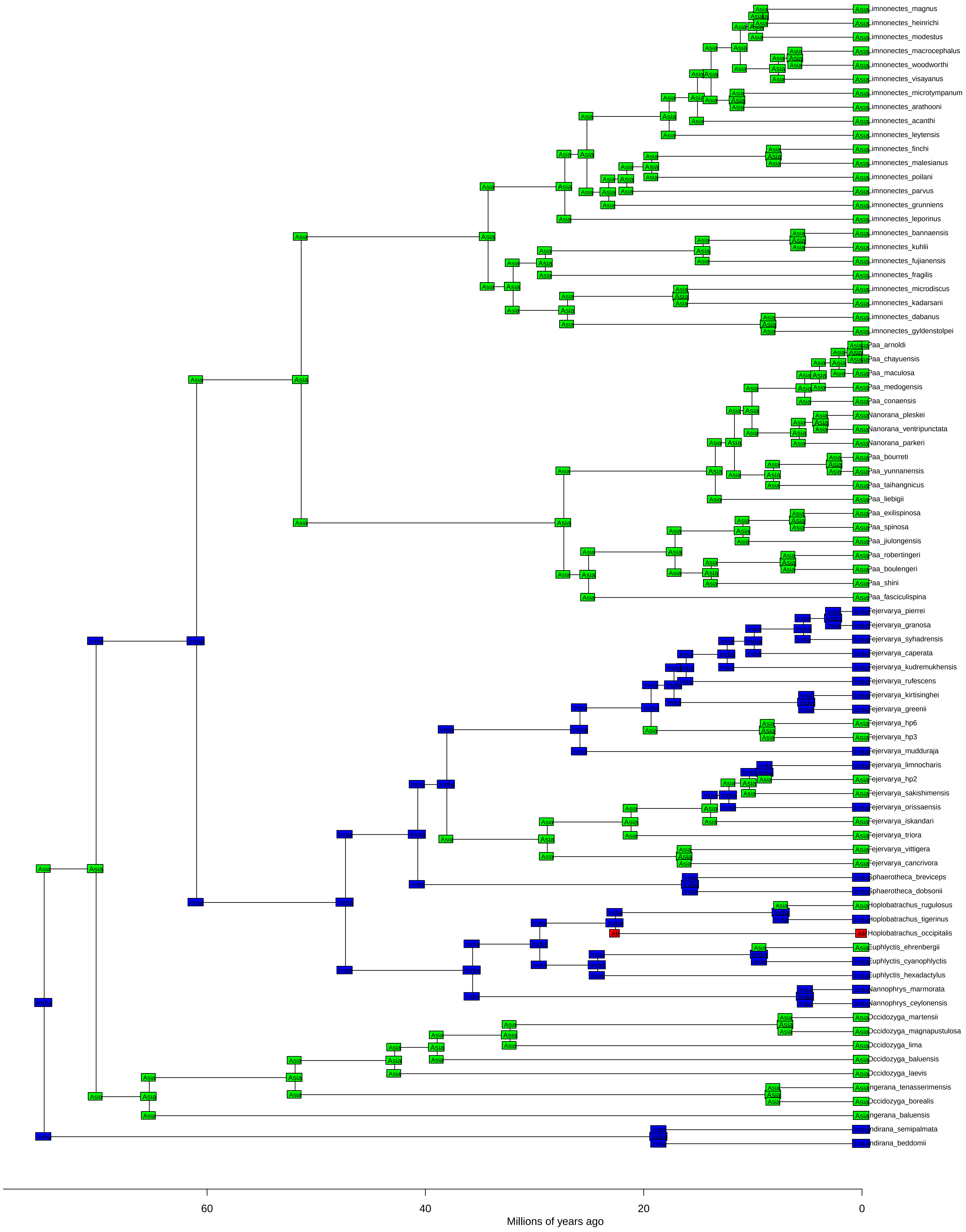

Dicroglossidae BioGeoBEARS DEC+J
ancstates: global optim, 2 areas max. d=0; e=0; j=0.0324; LnL=−39.67

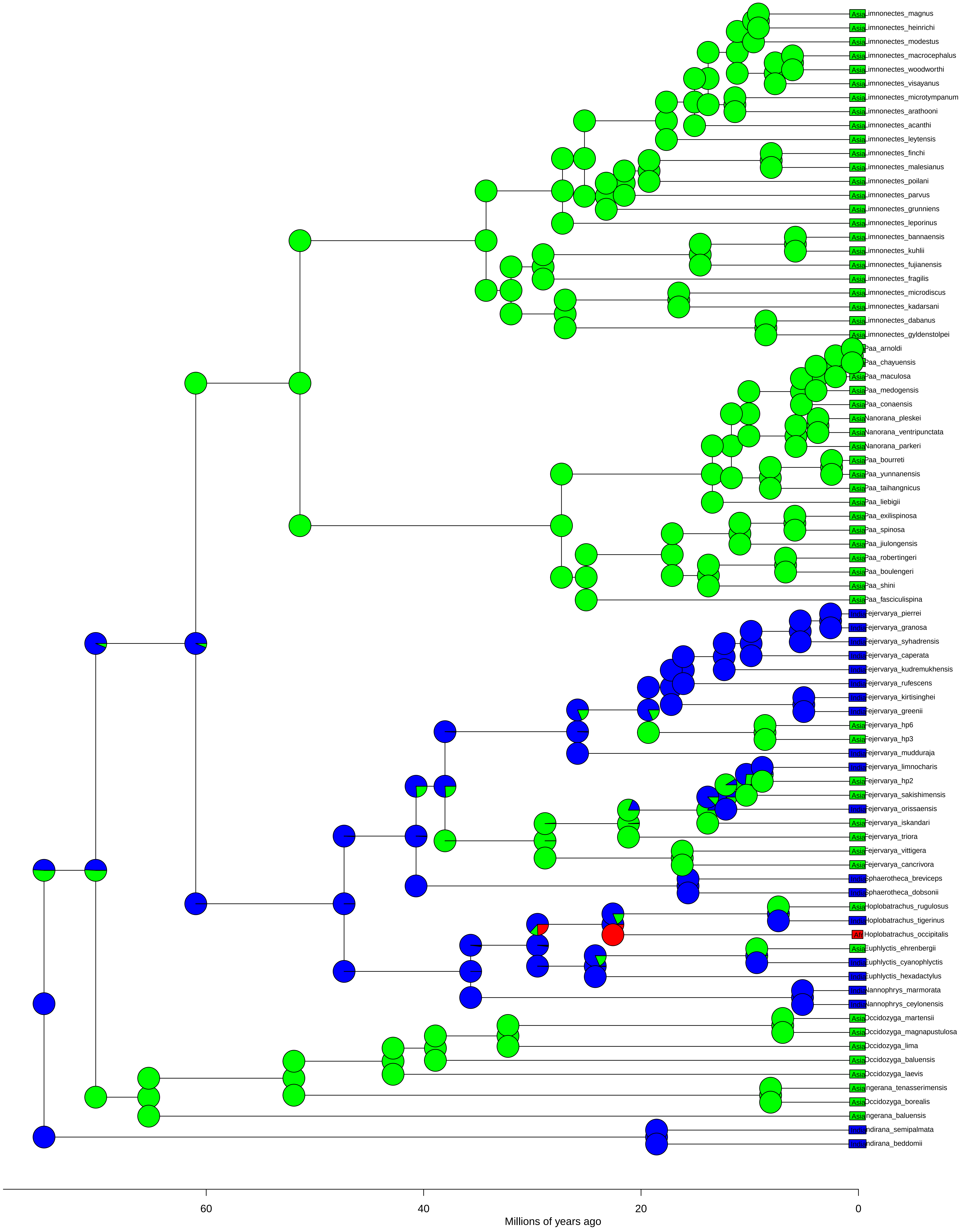

Rhacophoridae BioGeoBEARS DEC+J
ancstates: global optim, 2 areas max. d=0.0067; e=0; j=0.0019; LnL=−307.04

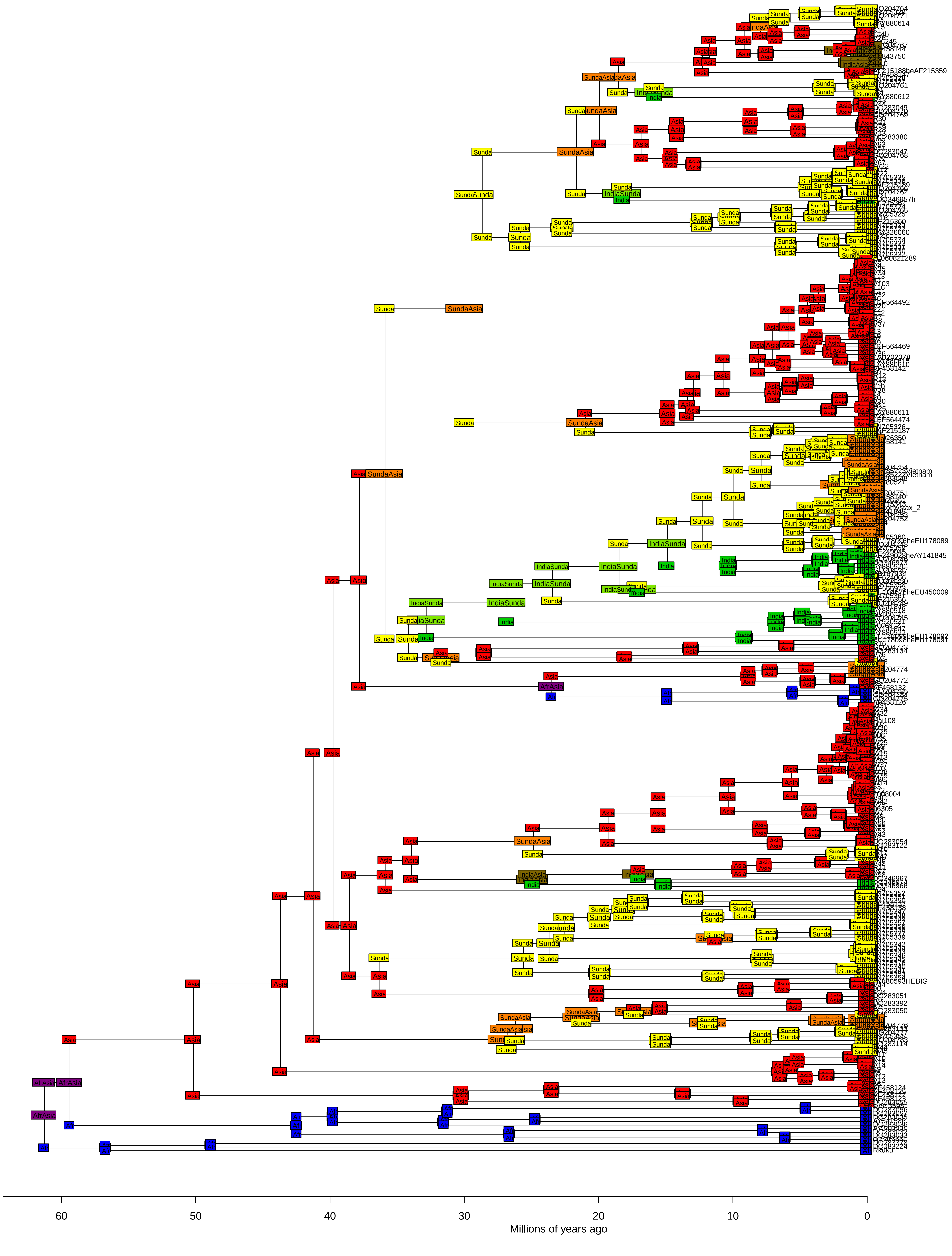

Rhacophoridae BioGeoBEARS DEC+J
ancstates: global optim, 2 areas max. d=0.0067; e=0; j=0.0019; LnL=−307.04

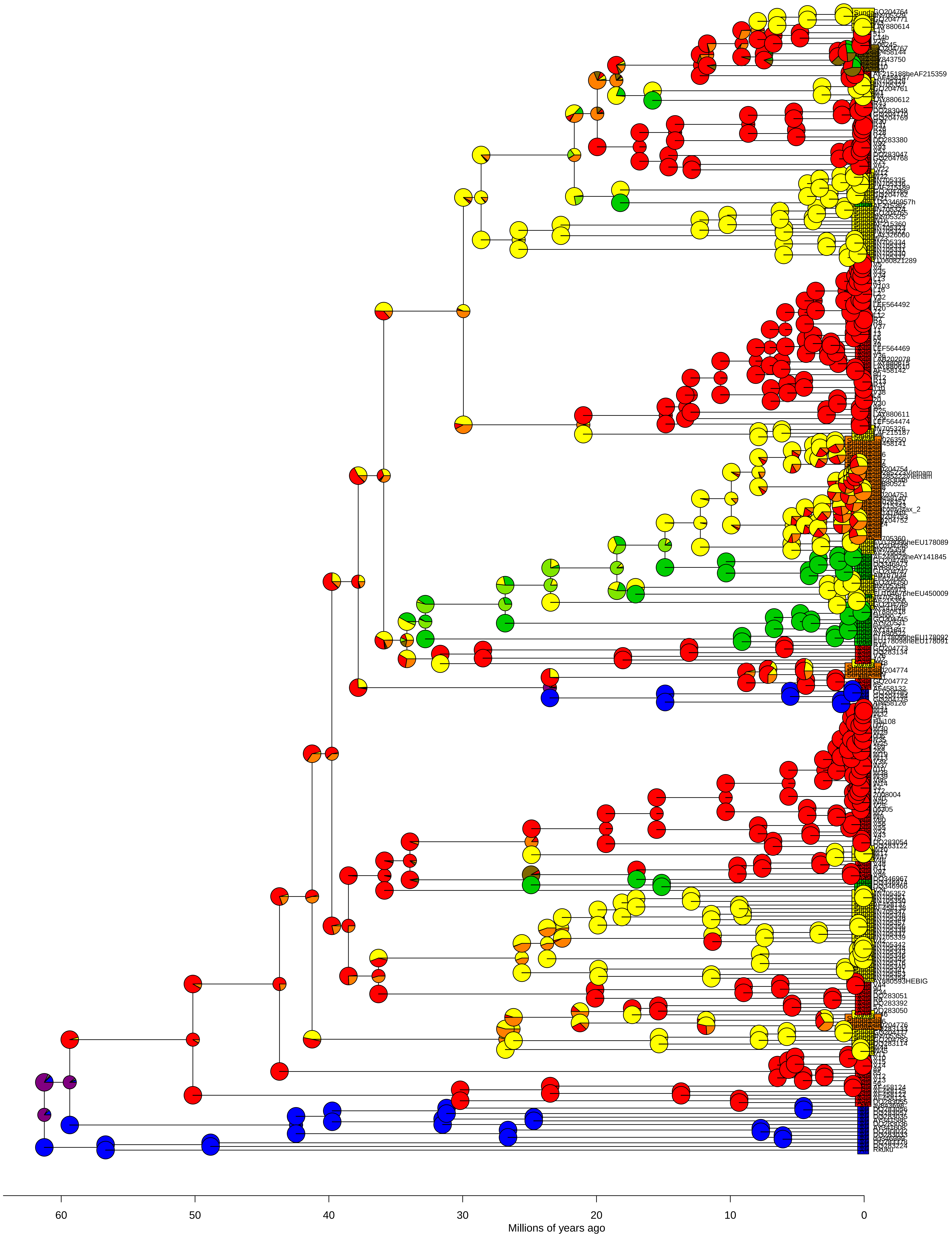

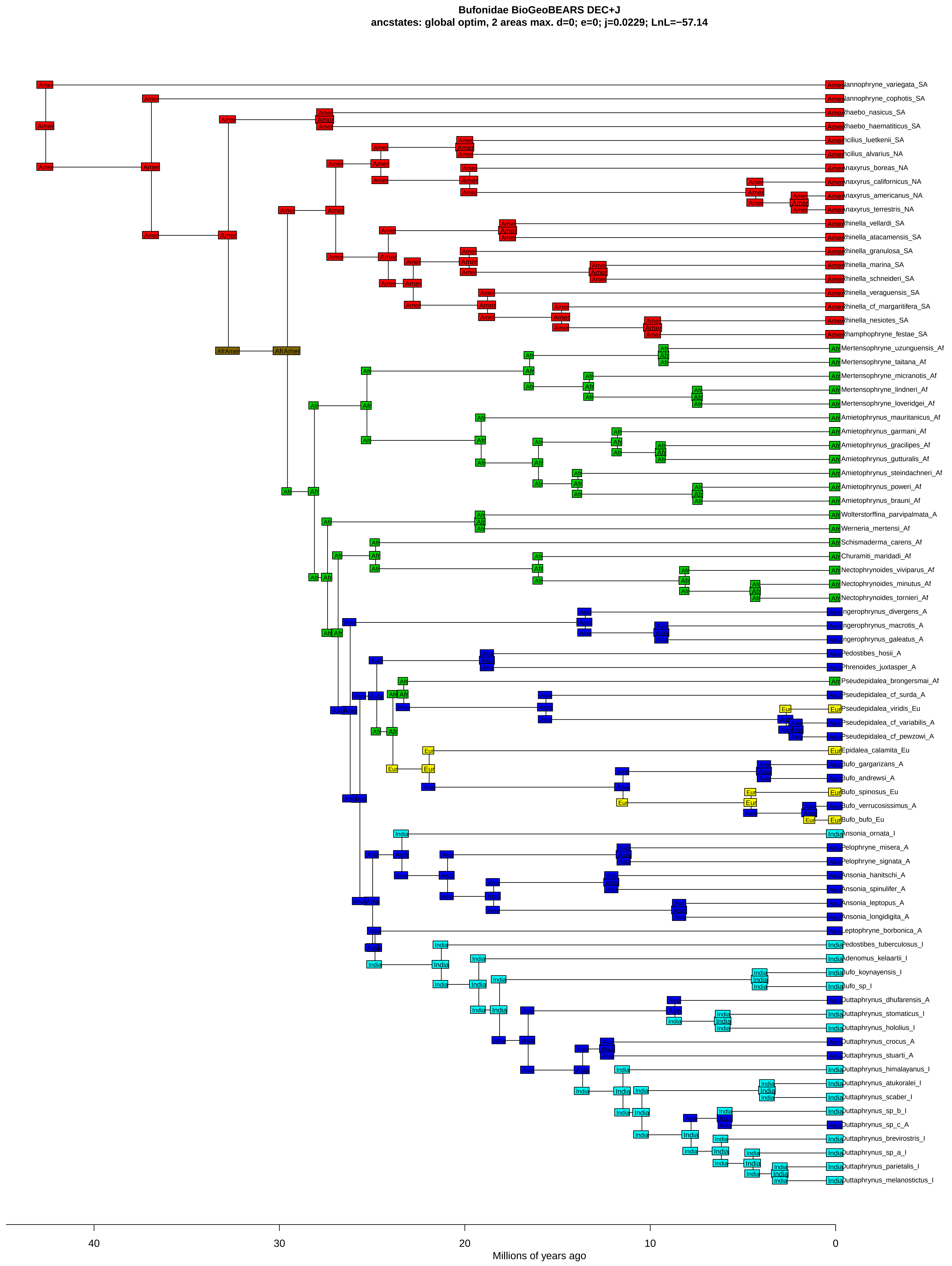
Bufonidae BioGeoBEARS DEC+J
ancstates: global optim, 2 areas max. d=0; e=0; j=0.0229; LnL=−57.14
lannophryne_variegata_SA
lannophryne_cophotis_SA
Rhaebo_nasicus_SA
Rhaebo_haematiticus_SA
ncilius_luetkenii_SA
ncilius_alvarius_NA
Anaxyrus_boreas_NA
Anaxyrus_californicus_NA
Anaxyrus_americanus_NA
Anaxyrus_terrestris_NA
Rhinella_vellardi_SA
Rhinella_atacamensis_SA
Rhinella_granulosa_SA
Rhinella_marina_SA
Rhinella_schneideri_SA
Rhinella_veraguensis_SA
hinella_cf_margaritifera_SA
Rhinella_nesiotes_SA
Rhamphophryne_festae_SA
Mertensophryne_uzunguensis_Af
Mertensophryne_taitana_Af
Mertensophryne_micranotis_Af
Mertensophryne_lindneri_Af
Mertensophryne_loveridgei_Af
Amietophrynus_mauritanicus_Af
Amietophrynus_garmani_Af
Amietophrynus_gracilipes_Af
Amietophrynus_gutturalis_Af
Amietophrynus_steindachneri_Af
Amietophrynus_poweri_Af
Amietophrynus_brauni_Af
Wolterstorffina_parvipalmata_A
Werneria_mertensi_Af
Schismaderma_carens_Af
Churamiti_maridadi_Af
Nectophrynoides_viviparus_Af
Nectophrynoides_minutus_Af
Nectophrynoides_tornieri_Af
ngerophrynus_divergens_A
ngerophrynus_macrotis_A
ngerophrynus_galeatus_A
Pedostibes_hosii_A
Phrenoides_juxtasper_A
Pseudepidalea_brongersmai_Af
Pseudepidalea_cf_surda_A
Pseudepidalea_viridis_Eu
Pseudepidalea_cf_variabilis_A
Pseudepidalea_cf_pewzowi_A
Epidalea_calamita_Eu
Bufo_gargarizans_A
Bufo_andrewsi_A
Bufo_spinosus_Eu
Bufo_verrucosissimus_A
Bufo_bufo_Eu
Ansonia_ornata_I
Pelophryne_misera_A
Pelophryne_signata_A
Ansonia_hanitschi_A
Ansonia_spinulifer_A
Ansonia_leptopus_A
Ansonia_longidigita_A
Leptophryne_borbonica_A
Pedostibes_tuberculosus_I
Adenomus_kelaartii_I
Bufo_koynayensis_I
Bufo_sp_I
Duttaphrynus_dhufarensis_A
Duttaphrynus_stomaticus_I
Duttaphrynus_hololius_I
Duttaphrynus_crocus_A
Duttaphrynus_stuarti_A
Duttaphrynus_himalayanus_I
Duttaphrynus_atukoralei_I
Duttaphrynus_scaber_I
Duttaphrynus_sp_b_I
Duttaphrynus_sp_c_A
Duttaphrynus_brevirostris_I
Duttaphrynus_sp_a_I
Duttaphrynus_parietalis_I
Duttaphrynus_melanostictus_I
40
30
20
10
0
Millions of years ago

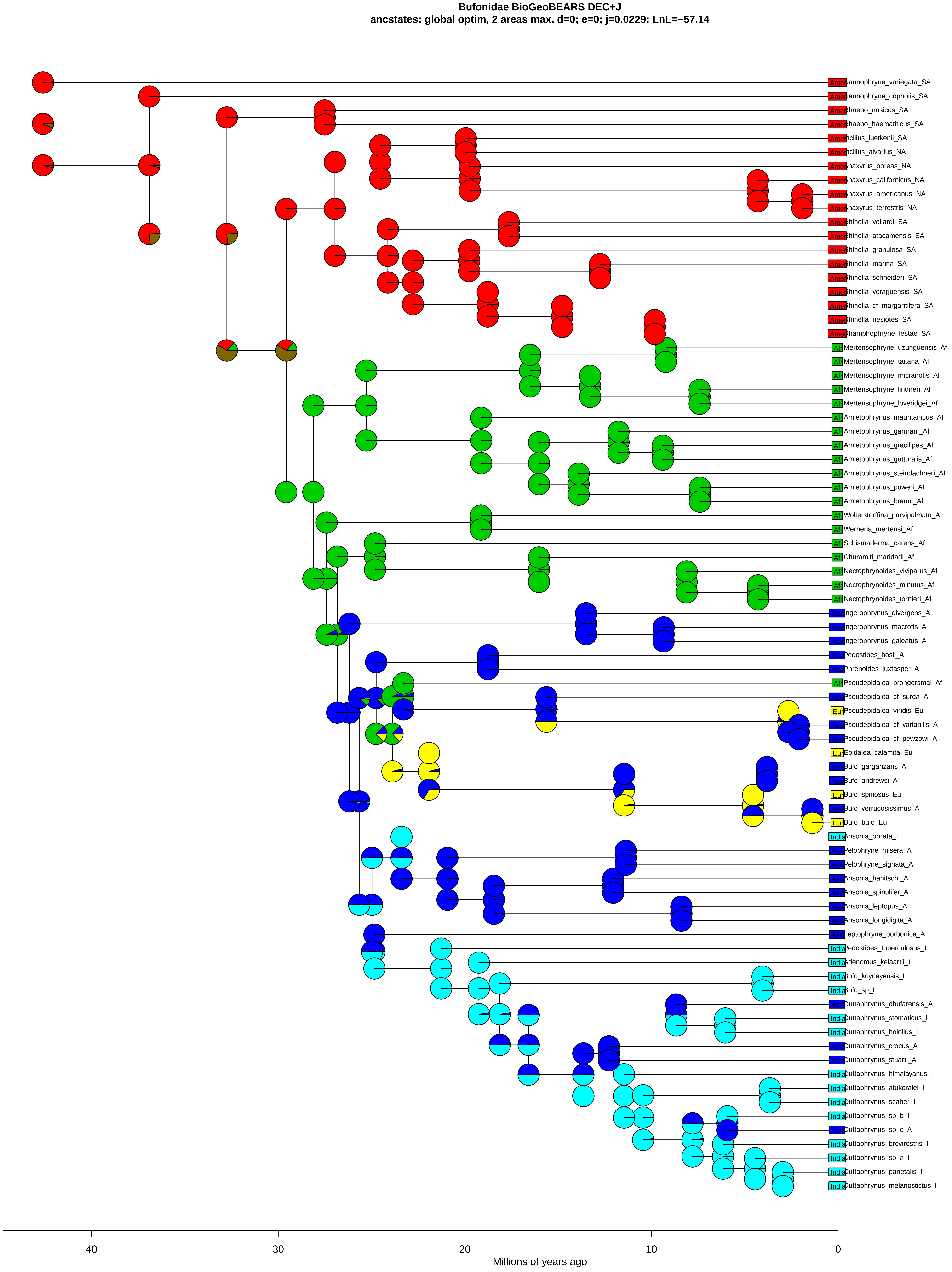

Bufonidae BioGeoBEARS DEC+J
ancstates: global optim, 2 areas max. d=0; e=0; j=0.0229; LnL=−57.14
lannophryne_variegata_SA
lannophryne_cophotis_SA
Rhaebo_nasicus_SA
Rhaebo_haematiticus_SA
ncilius_luetkenii_SA
ncilius_alvarius_NA
Anaxyrus_boreas_NA
Anaxyrus_californicus_NA
Anaxyrus_americanus_NA
Anaxyrus_terrestris_NA
Rhinella_vellardi_SA
Rhinella_atacamensis_SA
Rhinella_granulosa_SA
Rhinella_marina_SA
Rhinella_schneideri_SA
Rhinella_veraguensis_SA
Rhinella_cf_margaritifera_SA
Rhinella_nesiotes_SA
Rhamphophryne_festae_SA
Mertensophryne_uzunguensis_Af
Mertensophryne_taitana_Af
Mertensophryne_micranotis_Af
Mertensophryne_lindneri_Af
Mertensophryne_loveridgei_Af
Amietophrynus_mauritanicus_Af
Amietophrynus_garmani_Af
Amietophrynus_gracilipes_Af
Amietophrynus_gutturalis_Af
Amietophrynus_steindachneri_Af
Amietophrynus_poweri_Af
Amietophrynus_brauni_Af
Wolterstorffina_parvipalmata_A
Werneria_mertensi_Af
Schismaderma_carens_Af
Churamiti_maridadi_Af
Nectophrynoides_viviparus_Af
Nectophrynoides_minutus_Af
Nectophrynoides_tornieri_Af
ngerophrynus_divergens_A
ngerophrynus_macrotis_A
ngerophrynus_galeatus_A
Pedostibes_hosii_A
Phrenoides_juxtasper_A
Pseudepidalea_brongersmai_Af
Pseudepidalea_cf_surda_A
Pseudepidalea_viridis_Eu
Pseudepidalea_cf_variabilis_A
Pseudepidalea_cf_pewzowi_A
Epidalea_calamita_Eu
Bufo_gargarizans_A
Bufo_andrewsi_A
Bufo_spinosus_Eu
Bufo_verrucosissimus_A
Bufo_bufo_Eu
Ansonia_ornata_I
Pelophryne_misera_A
Pelophryne_signata_A
Ansonia_hanitschi_A
Ansonia_spinulifer_A
Ansonia_leptopus_A
Ansonia_longidigita_A
Leptophryne_borbonica_A
Pedostibes_tuberculosus_I
Adenomus_kelaartii_I
Bufo_koynayensis_I
Bufo_sp_I
Duttaphrynus_dhufarensis_A
Duttaphrynus_stomaticus_I
Duttaphrynus_hololius_I
Duttaphrynus_crocus_A
Duttaphrynus_stuarti_A
Duttaphrynus_himalayanus_I
Duttaphrynus_atukoralei_I
Duttaphrynus_scaber_I
Duttaphrynus_sp_b_I
Duttaphrynus_sp_c_A
Duttaphrynus_brevirostris_I
Duttaphrynus_sp_a_I
Duttaphrynus_parietalis_I
Duttaphrynus_melanostictus_I
40
30
20
10
0
Millions of years ago

Crocodylia BioGeoBEARS DEC+J
ancstates: global optim, 2 areas max. d=0.002; e=0; j=0.0797; LnL=−21.48

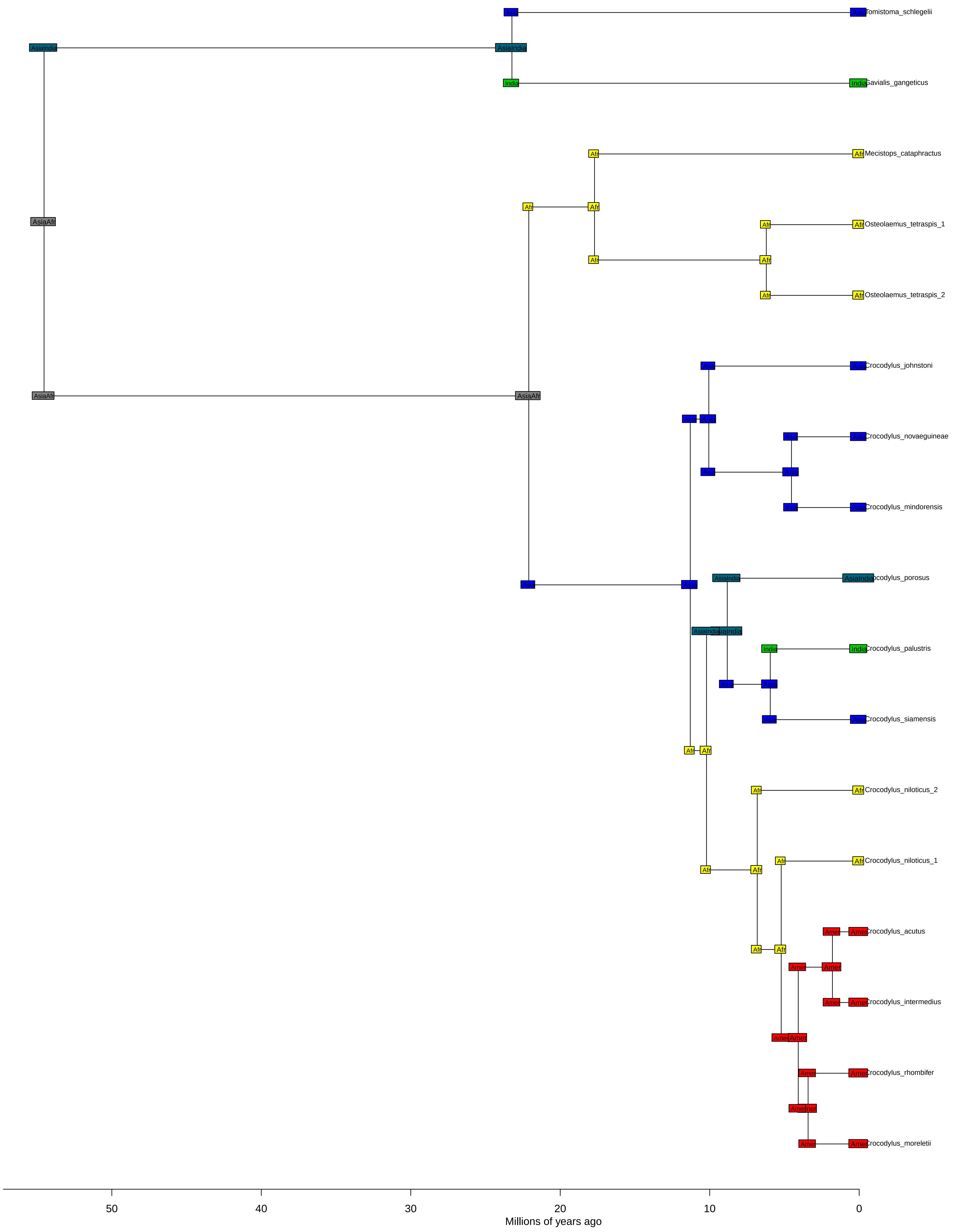

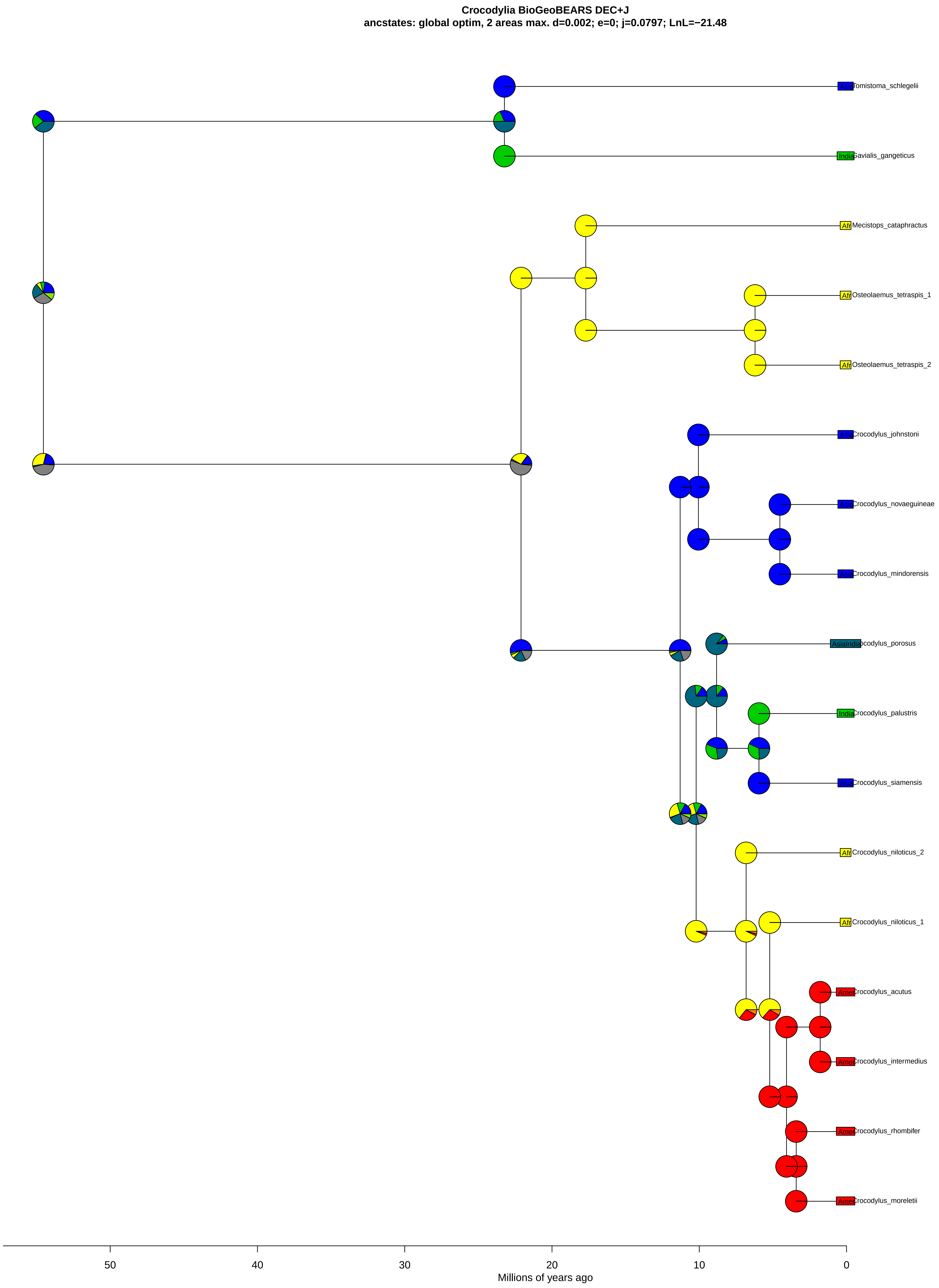

Crocodylia BioGeoBEARS DEC+J
ancstates: global optim, 2 areas max. d=0.002; e=0; j=0.0797; LnL=−21.48
Tomistoma_schlegelii
India
Gavialis_gangeticus
Afr
Mecistops_cataphractus
Afr
Osteolaemus_tetraspis_1
Afr
Osteolaemus_tetraspis_2
Crocodylus_johnstoni
Crocodylus_novaeguineae
Crocodylus_mindorensis
AsiaIndia
Crocodylus_porosus
India
Crocodylus_palustris
Crocodylus_siamensis
Afr
Crocodylus_niloticus_2
Afr
Crocodylus_niloticus_1
Amer
Crocodylus_acutus
Amer
Crocodylus_intermedius
Amer
Crocodylus_rhombifer
Amer
Crocodylus_moreletii
50
40
30
20
10
0
Millions of years ago

Cyrtodactylus BioGeoBEARS DEC+J
ancstates: global optim, 2 areas max. d=0; e=0; j=0.0501; LnL=−31.94

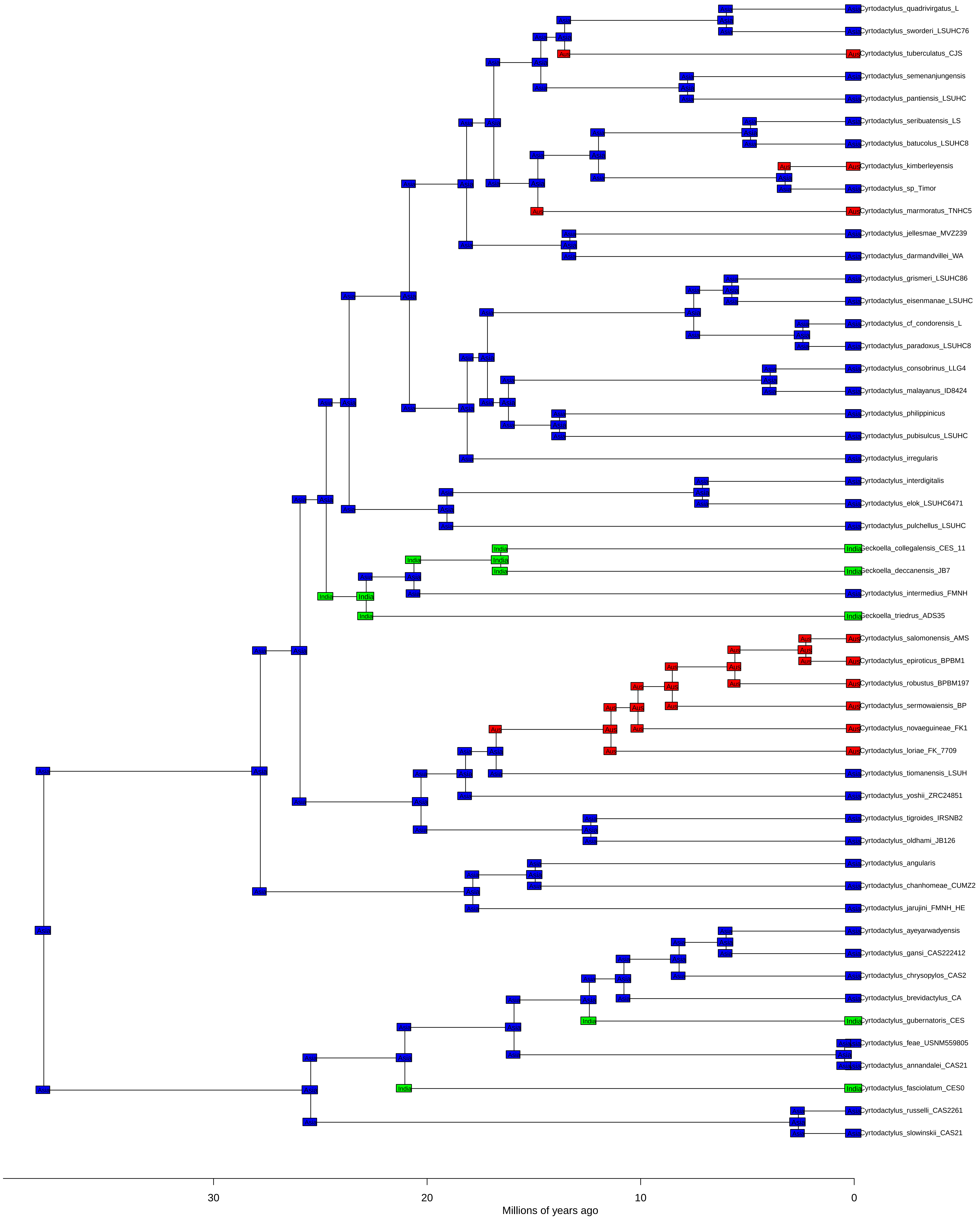

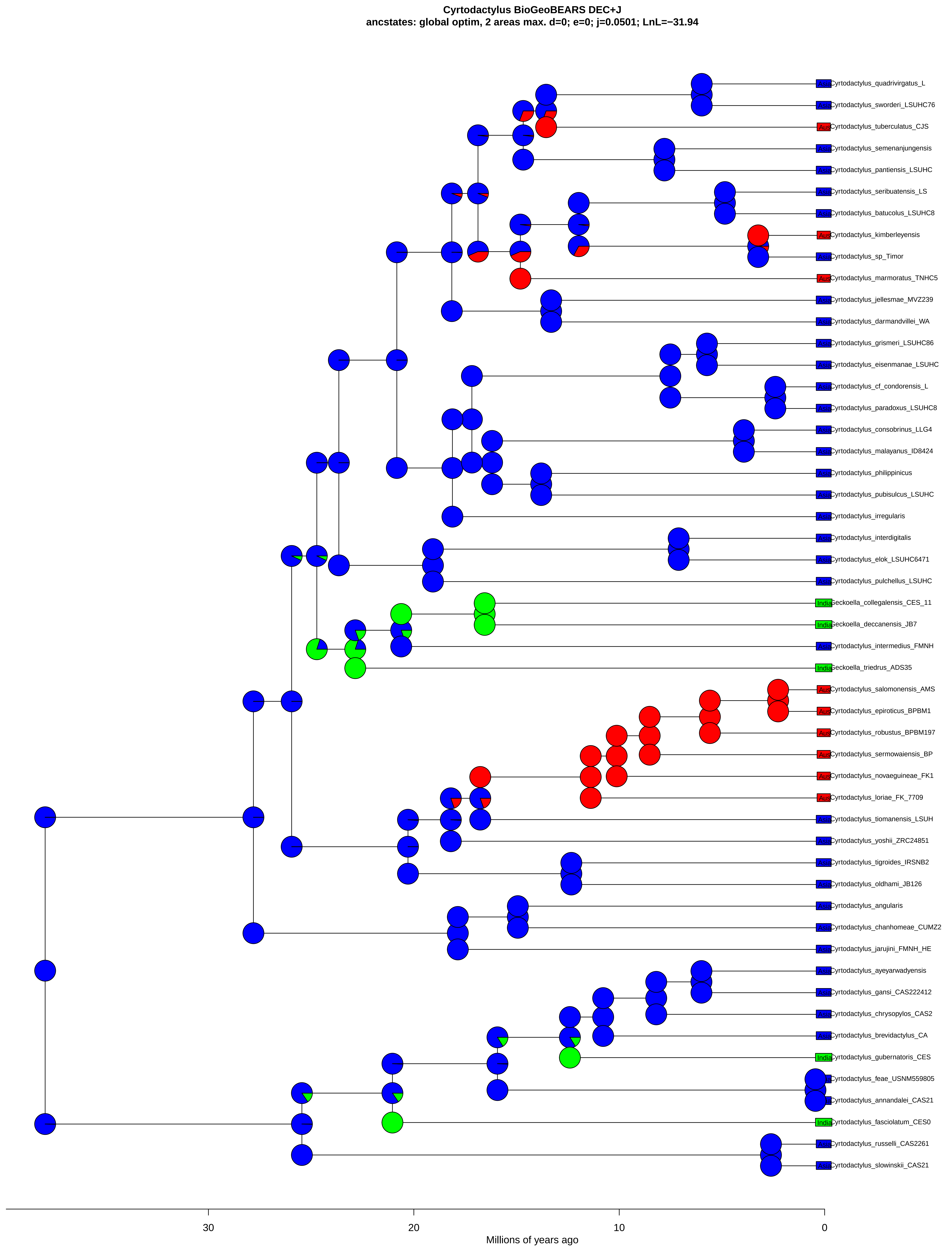
Cyrtodactylus BioGeoBEARS DEC+J
ancstates: global optim, 2 areas max. d=0; e=0; j=0.0501; LnL=−31.94
Cyrtodactylus_quadrivirgatus_L
Cyrtodactylus_sworderi_LSUHC76
Cyrtodactylus_tuberculatus_CJS
Cyrtodactylus_semenanjungensis
Cyrtodactylus_pantiensis_LSUHC
Cyrtodactylus_seribuatensis_LS
Cyrtodactylus_batucolus_LSUHC8
Cyrtodactylus_kimberleyensis
Cyrtodactylus_sp_Timor
Cyrtodactylus_marmoratus_TNHC5
Cyrtodactylus_jellesmae_MVZ239
Cyrtodactylus_darmandvillei_WA
Cyrtodactylus_grismeri_LSUHC86
Cyrtodactylus_eisenmanae_LSUHC
Cyrtodactylus_cf_condorensis_L
Cyrtodactylus_paradoxus_LSUHC8
Cyrtodactylus_consobrinus_LLG4
Cyrtodactylus_malayanus_ID8424
Cyrtodactylus_philippinicus
Cyrtodactylus_pubisulcus_LSUHC
Cyrtodactylus_irregularis
Cyrtodactylus_interdigitalis
Cyrtodactylus_elok_LSUHC6471
Cyrtodactylus_pulchellus_LSUHC
India Geckoella_collegalensis_CES_11
India Geckoella_deccanensis_JB7
Cyrtodactylus_intermedius_FMNH
India Geckoella_triedrus_ADS35
Aus Cyrtodactylus_salomonensis_AMS
Aus Cyrtodactylus_epiroticus_BPBM1
Aus Cyrtodactylus_robustus_BPBM197
Aus Cyrtodactylus_sermowaiensis_BP
Aus Cyrtodactylus_novaeguineae_FK1
Aus Cyrtodactylus_loriae_FK_7709
Cyrtodactylus_tiomanensis_LSUH
Cyrtodactylus_yoshii_ZRC24851
Cyrtodactylus_tigroides_IRSNB2
Cyrtodactylus_oldhami_JB126
Cyrtodactylus_angularis
Cyrtodactylus_chanhomeae_CUMZ2
Cyrtodactylus_jarujini_FMNH_HE
Cyrtodactylus_ayeyarwadyensis
Cyrtodactylus_gansi_CAS222412
Cyrtodactylus_chrysopylos_CAS2
Cyrtodactylus_brevidactylus_CA
India Cyrtodactylus_gubernatoris_CES
Cyrtodactylus_feae_USNM559805
Cyrtodactylus_annandalei_CAS21
India Cyrtodactylus_fasciolatum_CES0
Cyrtodactylus_russelli_CAS2261
Cyrtodactylus_slowinskii_CAS21
30
20
10
0
Millions of years ago

Scincidae BioGeoBEARS DEC+J
ancstates: global optim, 2 areas max. d=0; e=0; j=0.0232; LnL=−47.68

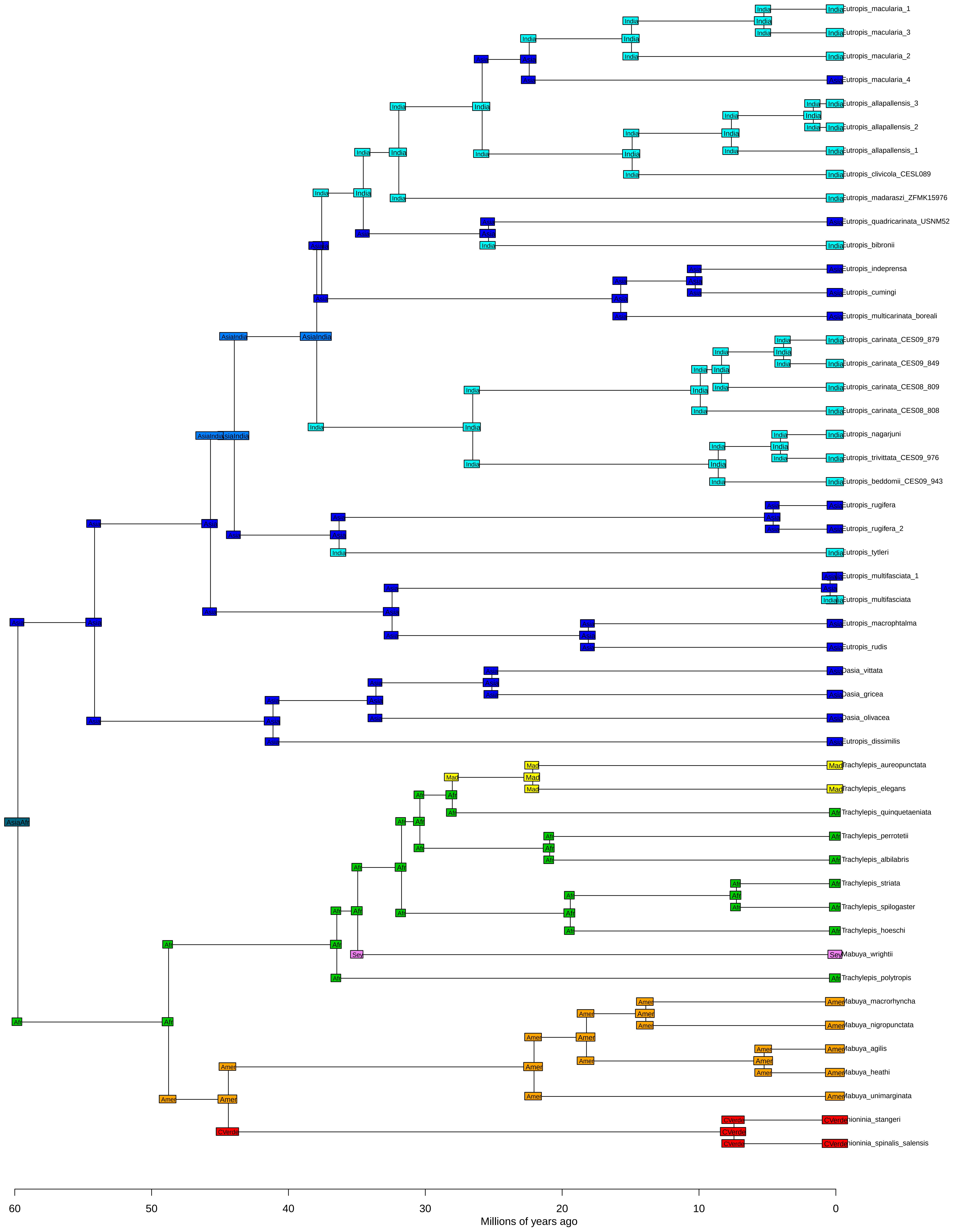

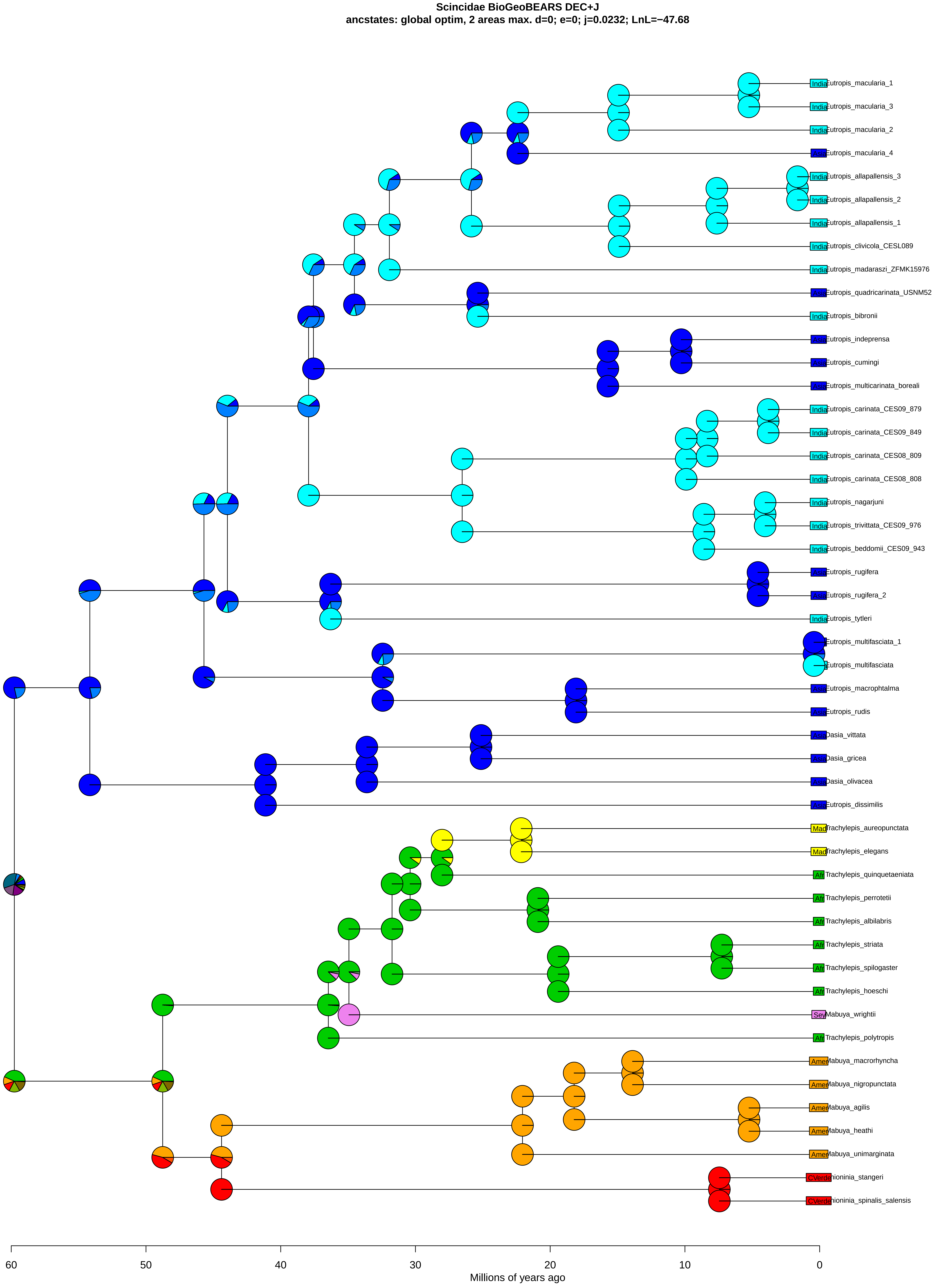

Scincidae BioGeoBEARS DEC+J
ancstates: global optim, 2 areas max. d=0; e=0; j=0.0232; LnL=−47.68
India Eutropis_macularia_1
India Eutropis_macularia_3
India Eutropis_macularia_2
Eutropis_macularia_4
India Eutropis_allapallensis_3
India Eutropis_allapallensis_2
India Eutropis_allapallensis_1
India Eutropis_clivicola_CESL089
India Eutropis_madaraszi_ZFMK15976
Eutropis_quadricarinata_USNM52
India Eutropis_bibronii
Eutropis_indeprensa
Eutropis_cumingi
Eutropis_multicarinata_boreali
India Eutropis_carinata_CES09_879
India Eutropis_carinata_CES09_849
India Eutropis_carinata_CES08_809
India Eutropis_carinata_CES08_808
India Eutropis_nagarjuni
India Eutropis_trivittata_CES09_976
India Eutropis_beddomii_CES09_943
Eutropis_rugifera
Eutropis_rugifera_2
India Eutropis_tytleri
Eutropis_multifasciata_1
Eutropis_multifasciata
Eutropis_macrophtalma
Eutropis_rudis
Dasia_vittata
Dasia_gricea
Dasia_olivacea
Eutropis_dissimilis
Mad Trachylepis_aureopunctata
Mad Trachylepis_elegans
Afr Trachylepis_quinquetaeniata
Afr Trachylepis_perrotetii
Afr Trachylepis_albilabris
Afr Trachylepis_striata
Afr Trachylepis_spilogaster
Afr Trachylepis_hoeschi
Sey Mabuya_wrightii
Afr Trachylepis_polytropis
Amer Mabuya_macrorhyncha
Amer Mabuya_nigropunctata
Amer Mabuya_agilis
Amer Mabuya_heathi
Amer Mabuya_unimarginata
CVerde hioninia_stangeri
CVerde hioninia_spinalis_salensis
60
50
40
30
20
10
0
Millions of years ago

Acrodonta BioGeoBEARS DEC+J
ancstates: global optim, 2 areas max. d=0.0024; e=0; j=0.038; LnL=−44.36

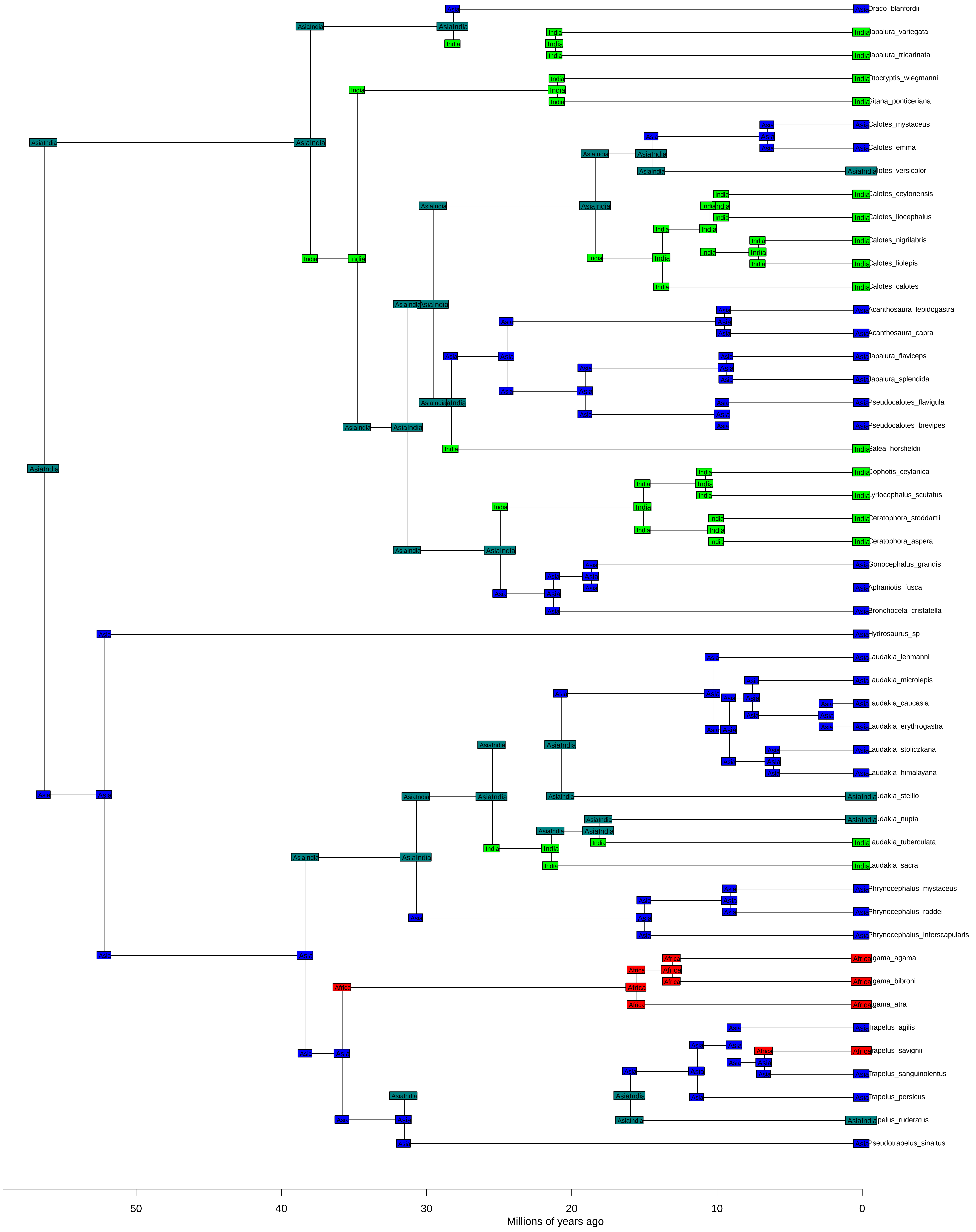

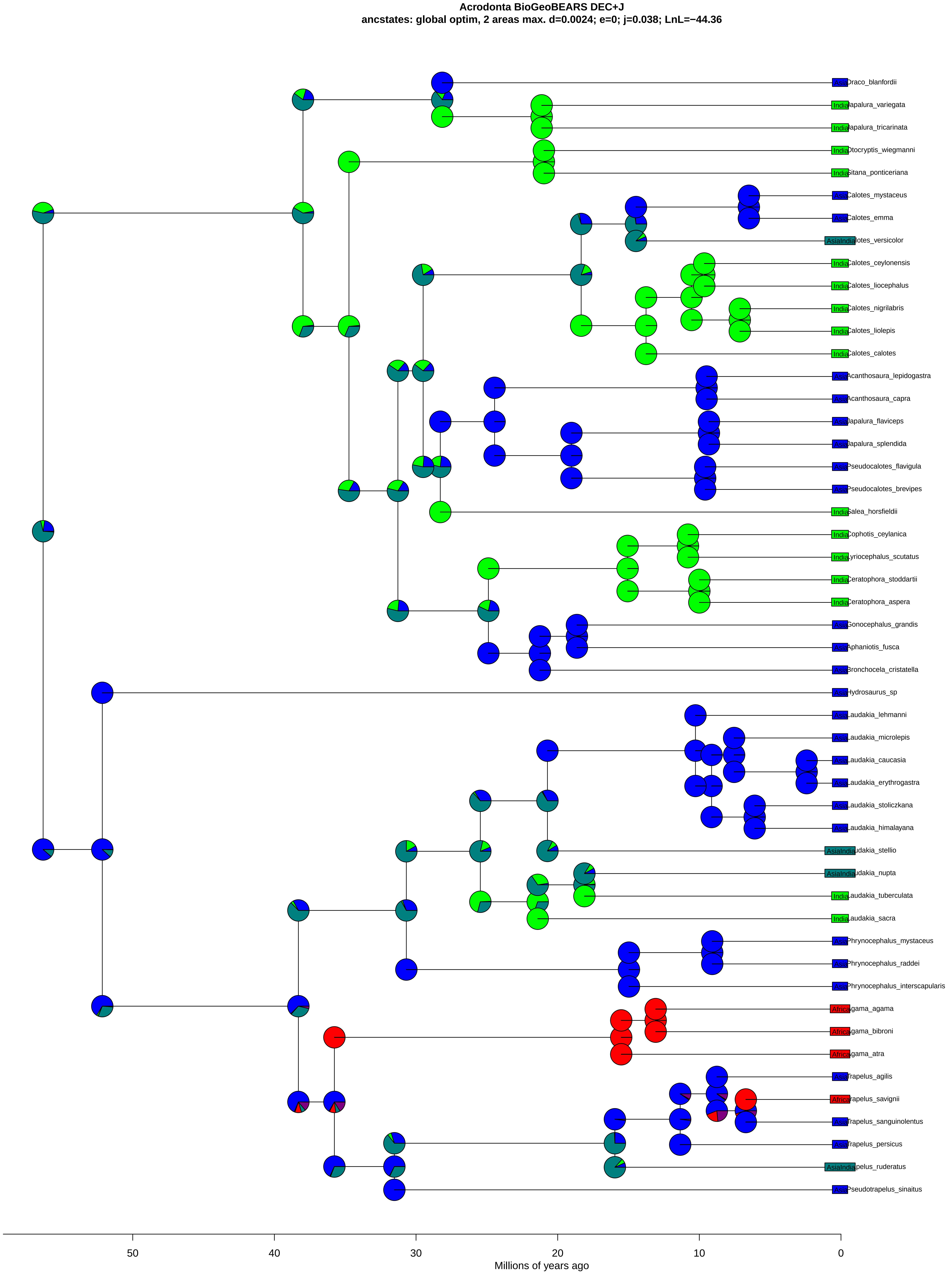

Acrodonta BioGeoBEARS DEC+J
ancstates: global optim, 2 areas max. d=0.0024; e=0; j=0.038; LnL=−44.36
Draco_blanfordii
India Japalura_variegata
India Japalura_tricarinata
India Otocryptis_wiegmanni
India Sitana_ponticeriana
Calotes_mystaceus
Calotes_emma
AsiaIndia ...lotes_versicolor
India Calotes_ceylonensis
India Calotes_liocephalus
India Calotes_nigrilabris
India Calotes_liolepis
India Calotes_calotes
Acanthosaura_lepidogastra
Acanthosaura_capra
Japalura_flaviceps
Japalura_splendida
Pseudocalotes_flavigula
Pseudocalotes_brevipes
India Salea_horsfieldii
India Cophotis_ceylanica
India ...yriocephalus_scutatus
India Ceratophora_stoddartii
India Ceratophora_aspera
Gonocephalus_grandis
Aphaniotis_fusca
Bronchocela_cristatella
Hydrosaurus_sp
Laudakia_lehmanni
Laudakia_microlepis
Laudakia_caucasia
Laudakia_erythrogastra
Laudakia_stoliczkana
Laudakia_himalayana
AsiaIndia ...udakia_stellio
AsiaIndia ...udakia_nupta
India Laudakia_tuberculata
India Laudakia_sacra
Phrynocephalus_mystaceus
Phrynocephalus_raddei
Phrynocephalus_interscapularis
Africa ...gama_agama
Africa ...gama_bibroni
Africa ...gama_atra
Trapelus_agilis
Africa ...rapelus_savignii
Trapelus_sanguinolentus
Trapelus_persicus
AsiaIndia ...pelus_ruderatus
Pseudotrapelus_sinaitus
50
40
30
20
10
0
Millions of years ago

Agamidae−2 BioGeoBEARS DEC+J
ancstates: global optim, 2 areas max. d=0; e=0; j=0.0283; LnL=−4.58

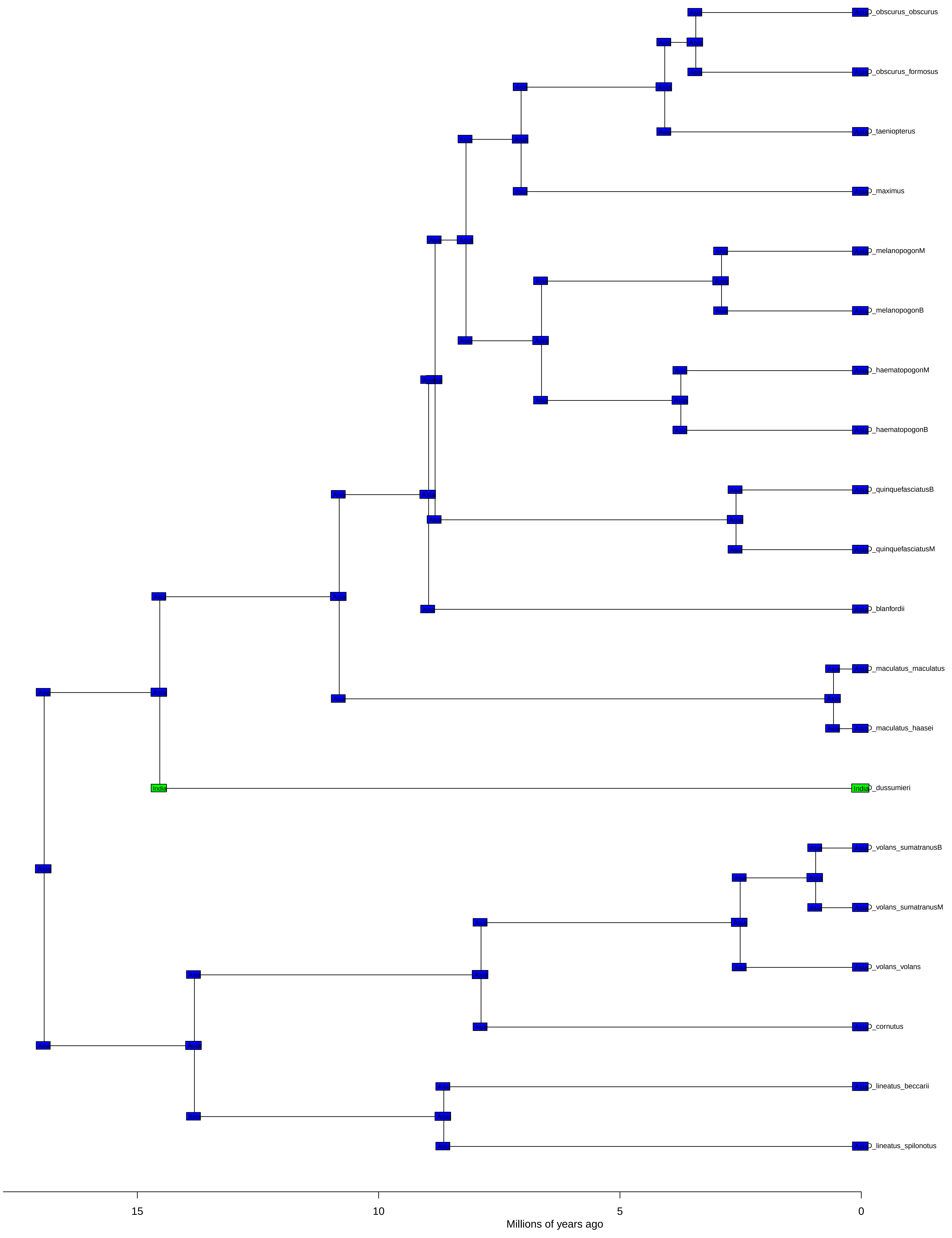

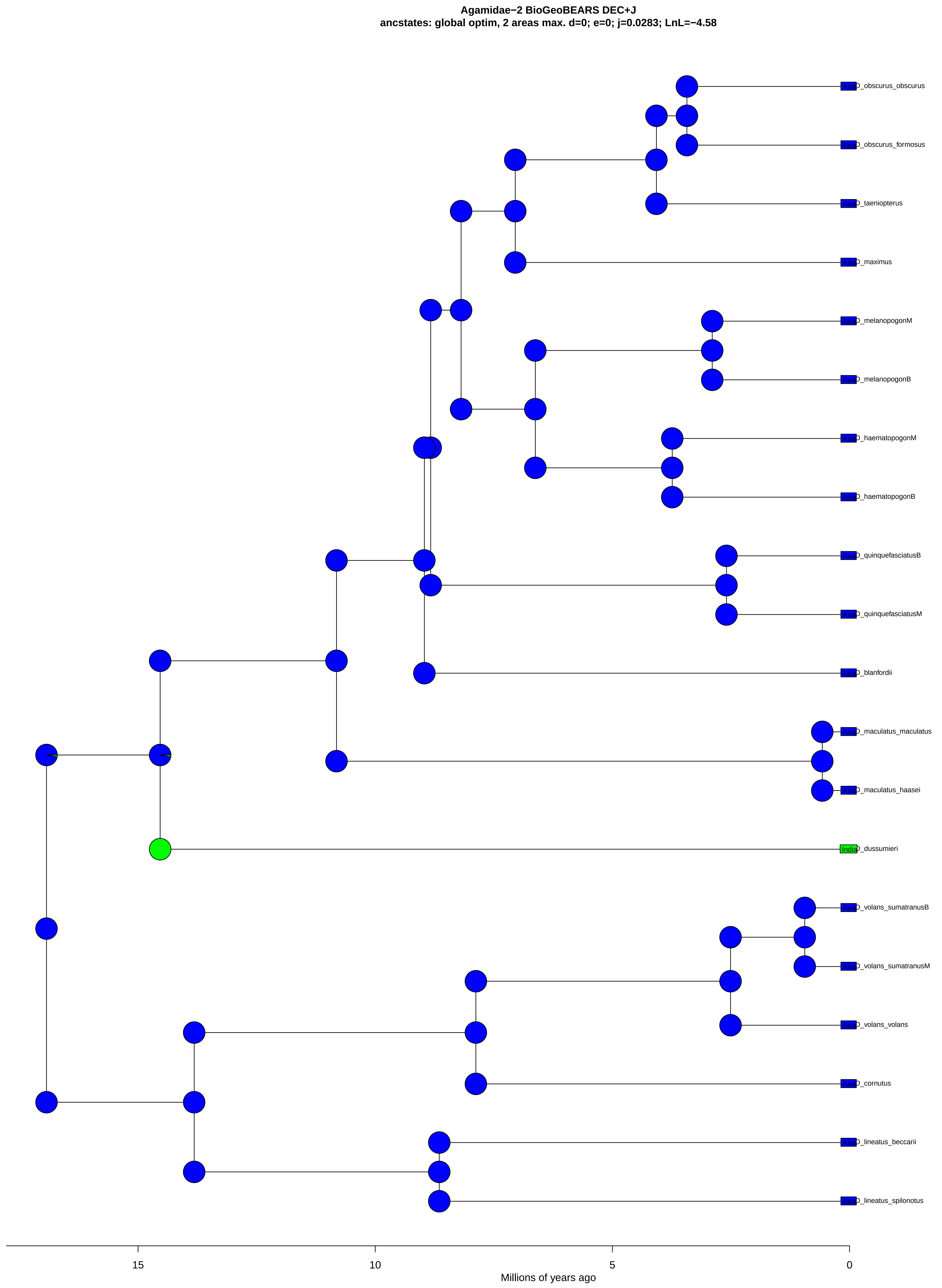

Agamidae−2 BioGeoBEARS DEC+J
ancstates: global optim, 2 areas max. d=0; e=0; j=0.0283; LnL=−4.58
D_obscurus_obscurus
D_obscurus_formosus
D_taeniopterus
D_maximus
D_melanopogonM
D_melanopogonB
D_haematopogonM
D_haematopogonB
D_quinquefasciatusB
D_quinquefasciatusM
D_blanfordii
D_maculatus_maculatus
D_maculatus_haasei
India
D_dussumieri
D_volans_sumatranusB
D_volans_sumatranusM
D_volans_volans
D_cornutus
D_lineatus_beccarii
D_lineatus_spilonotus
15
10
5
0
Millions of years ago

Boidae BioGeoBEARS DEC+J
ancstates: global optim, 2 areas max. d=5e−04; e=0; j=0.0422; LnL=−40.40

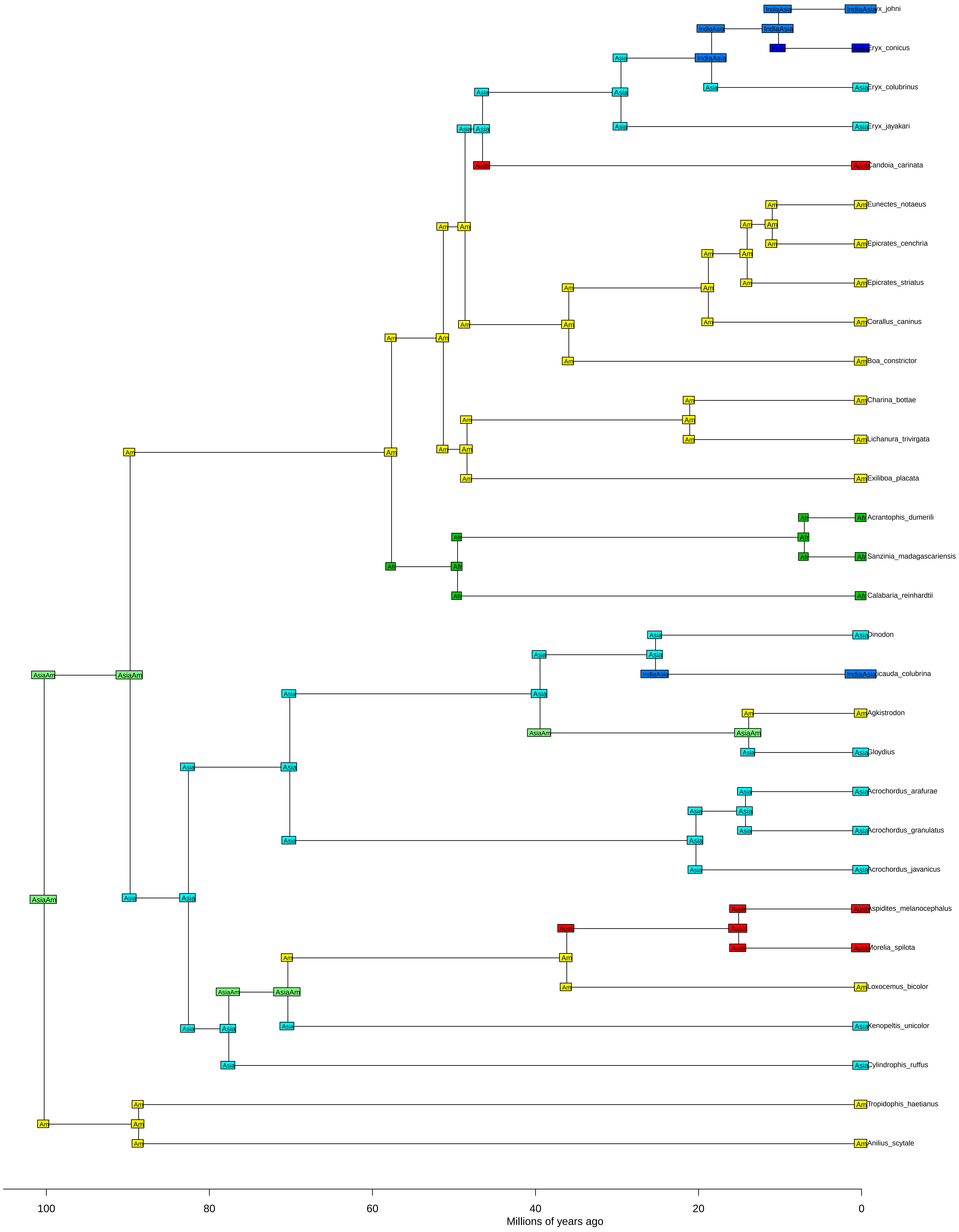

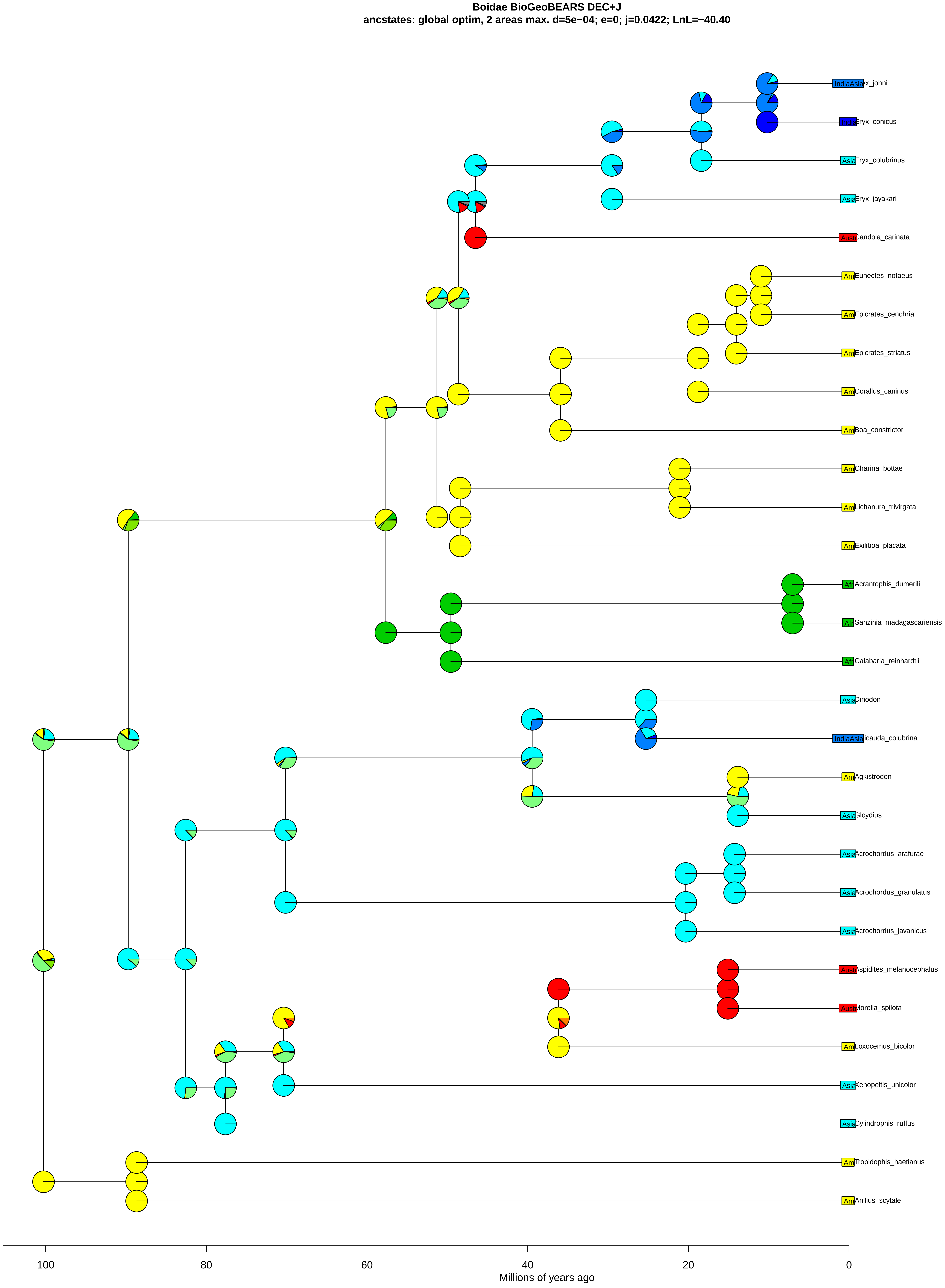

Boidae BioGeoBEARS DEC+J
ancstates: global optim, 2 areas max. d=5e−04; e=0; j=0.0422; LnL=−40.40
IndiaAsia Eryx_johni
Eryx_conicus
Asia Eryx_colubrinus
Asia Eryx_jayakari
Austr Candoia_carinata
Am Eunectes_notaeus
Am Epicrates_cenchria
Am Epicrates_striatus
Am Corallus_caninus
Am Boa_constrictor
Am Charina_bottae
Am Lichanura_trivirgata
Am Exiliboa_placata
Afr Acrantophis_dumerili
Afr Sanzinia_madagascariensis
Afr Calabaria_reinhardtii
Asia Dinodon
IndiaAsia icauda_colubrina
Am Agkistrodon
Asia Gloydius
Asia Acrochordus_arafurae
Asia Acrochordus_granulatus
Asia Acrochordus_javanicus
Austr Aspidites_melanocephalus
Austr Morelia_spilota
Am Loxocemus_bicolor
Asia Xenopeltis_unicolor
Asia Cylindrophis_ruffus
Am Tropidophis_haetianus
Am Anilius_scytale
100
80
60
40
20
0
Millions of years ago

Viperidae BioGeoBEARS DEC+J
ancstates: global optim, 2 areas max. d=7e−04; e=0; j=0.0211; LnL=−101.90

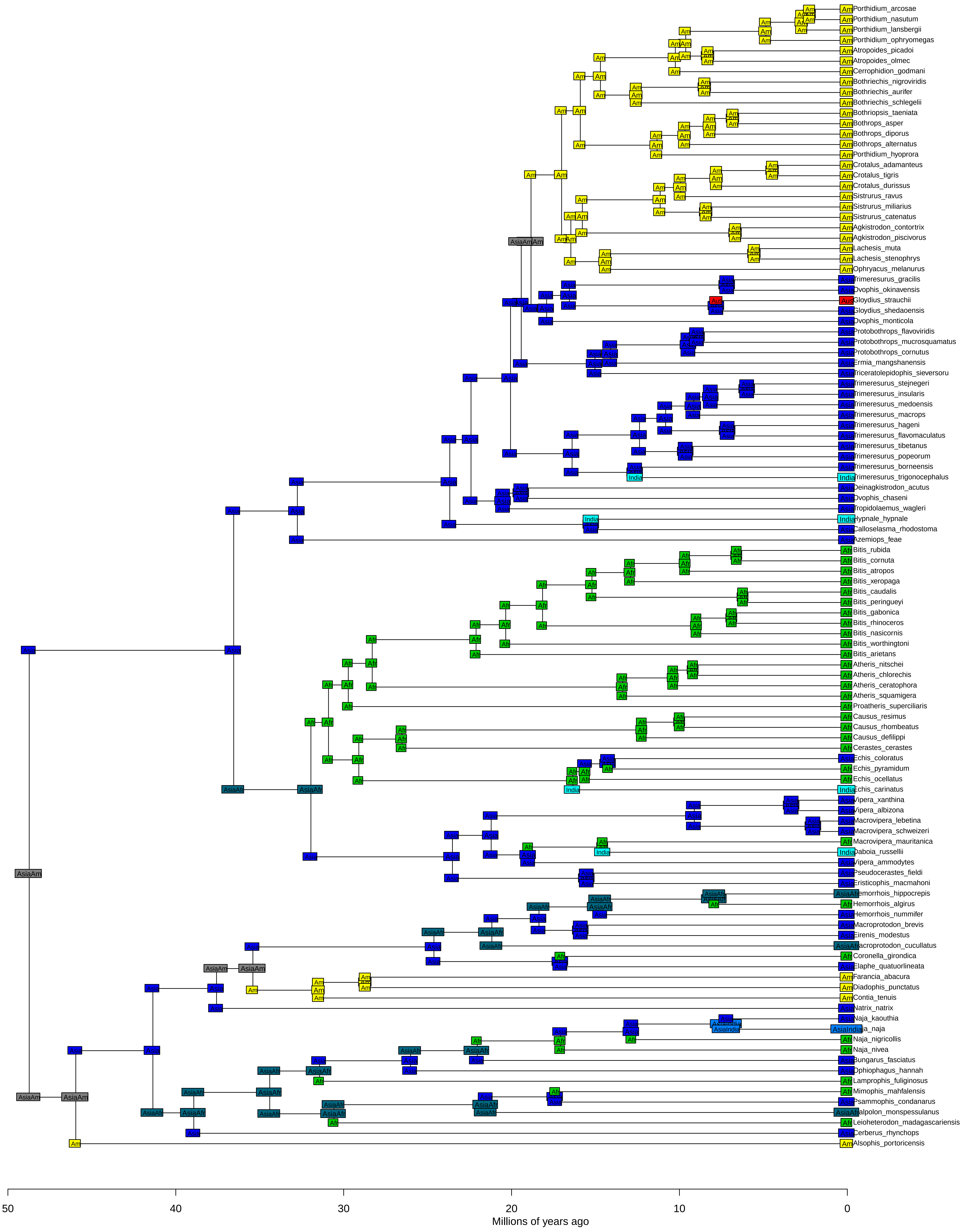

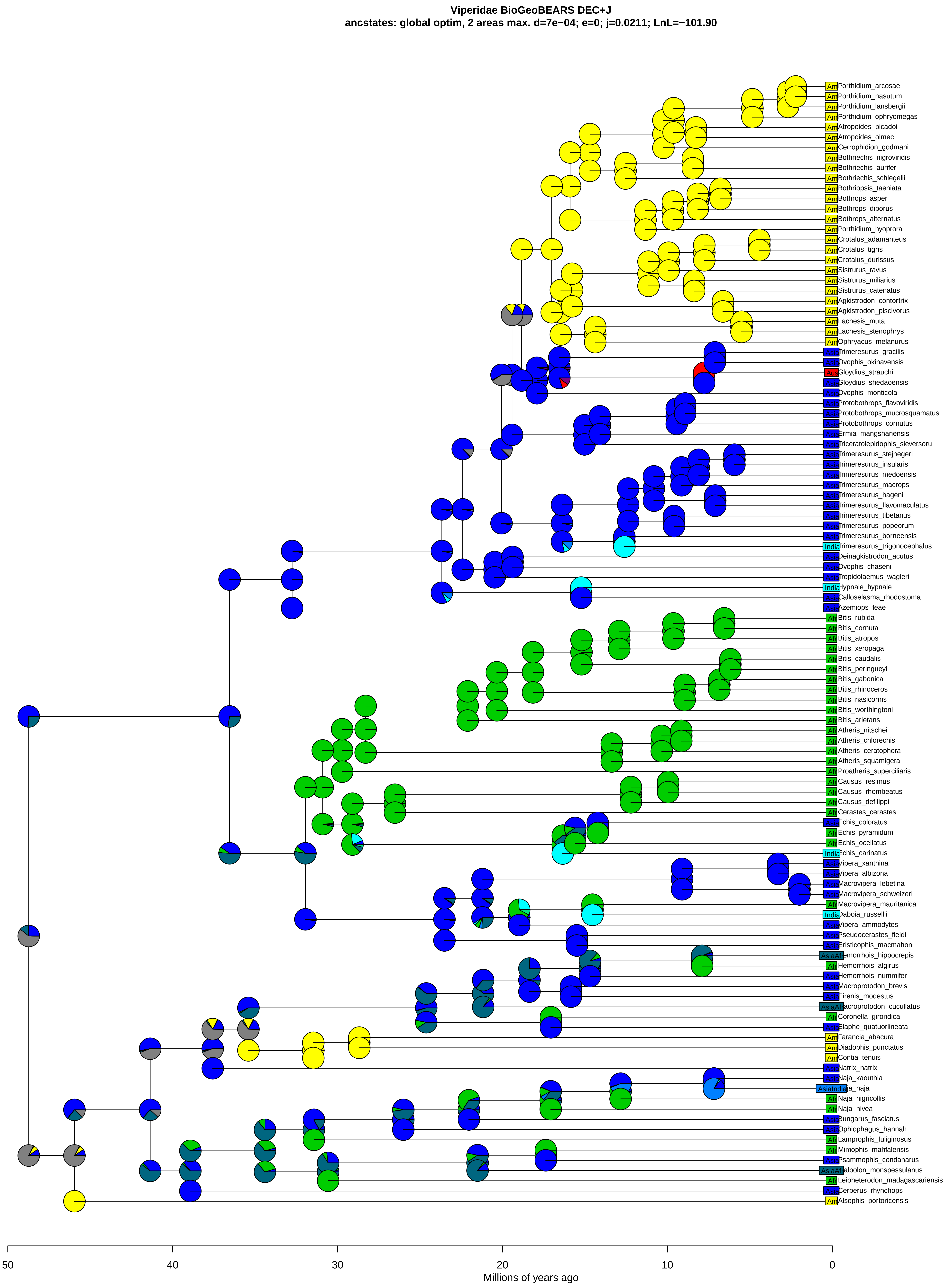

Viperidae BioGeoBEARS DEC+J
ancstates: global optim, 2 areas max. d=7e−04; e=0; j=0.0211; LnL=−101.90
Porthidium_arcosae
Porthidium_nasutum
Porthidium_lansbergii
Porthidium_ophryomegas
Atropoides_picadoi
Atropoides_olmec
Cerrophidion_godmani
Bothriechis_nigroviridis
Bothriechis_aurifer
Bothriechis_schlegelii
Bothriopsis_taeniata
Bothrops_asper
Bothrops_diporus
Bothrops_alternatus
Porthidium_hyoprora
Crotalus_adamanteus
Crotalus_tigris
Crotalus_durissus
Sistrurus_ravus
Sistrurus_miliarius
Sistrurus_catenatus
Agkistrodon_contortrix
Agkistrodon_piscivorus
Lachesis_muta
Lachesis_stenophrys
Ophryacus_melanurus
Trimeresurus_gracilis
Ovophis_okinavensis
Gloydius_strauchii
Gloydius_shedaoensis
Ovophis_monticola
Protobothrops_flavoviridis
Protobothrops_mucrosquamatus
Protobothrops_cornutus
Ermia_mangshanensis
Triceratolepidophis_sieversoru
Trimeresurus_stejnegeri
Trimeresurus_insularis
Trimeresurus_medoensis
Trimeresurus_macrops
Trimeresurus_hageni
Trimeresurus_flavomaculatus
Trimeresurus_tibetanus
Trimeresurus_popeorum
Trimeresurus_borneensis
Trimeresurus_trigonocephalus
Deinagkistrodon_acutus
Ovophis_chaseni
Tropidolaemus_wagleri
Hypnale_hypnale
Calloselasma_rhodostoma
Azemiops_feae
Bitis_rubida
Bitis_cornuta
Bitis_atropos
Bitis_xeropaga
Bitis_caudalis
Bitis_peringueyi
Bitis_gabonica
Bitis_rhinoceros
Bitis_nasicornis
Bitis_worthingtoni
Bitis_arietans
Atheris_nitschei
Atheris_chlorechis
Atheris_ceratophora
Atheris_squamigera
Proatheris_superciliaris
Causus_resimus
Causus_rhombeatus
Causus_defilippi
Cerastes_cerastes
Echis_coloratus
Echis_pyramidum
Echis_ocellatus
Echis_carinatus
Vipera_xanthina
Vipera_albizona
Macrovipera_lebetina
Macrovipera_schweizeri
Macrovipera_mauritanica
Daboia_russellii
Vipera_ammodytes
Pseudocerastes_fieldi
Eristicophis_macmahoni
emorrhois_hippocrepis
Hemorrhois_algirus
Hemorrhois_nummifer
Macroprotodon_brevis
Eirenis_modestus
acroprotodon_cucullatus
Coronella_girondica
Elaphe_quatuorlineata
Farancia_abacura
Diadophis_punctatus
Contia_tenuis
Natrix_natrix
Naja_kaouthia
ja_naja
Naja_nigricollis
Naja_nivea
Bungarus_fasciatus
Ophiophagus_hannah
Lamprophis_fuliginosus
Mimophis_mahfalensis
Psammophis_condanarus
alpolon_monspessulanus
Leioheterodon_madagascariensis
Cerberus_rhynchops
Alsophis_portoricensis
50
40
30
20
10
0
Millions of years ago

Certhiidae BioGeoBEARS DEC
ancstates: global optim, 2 areas max. d=0.1046; e=0; j=0; LnL=−23.68

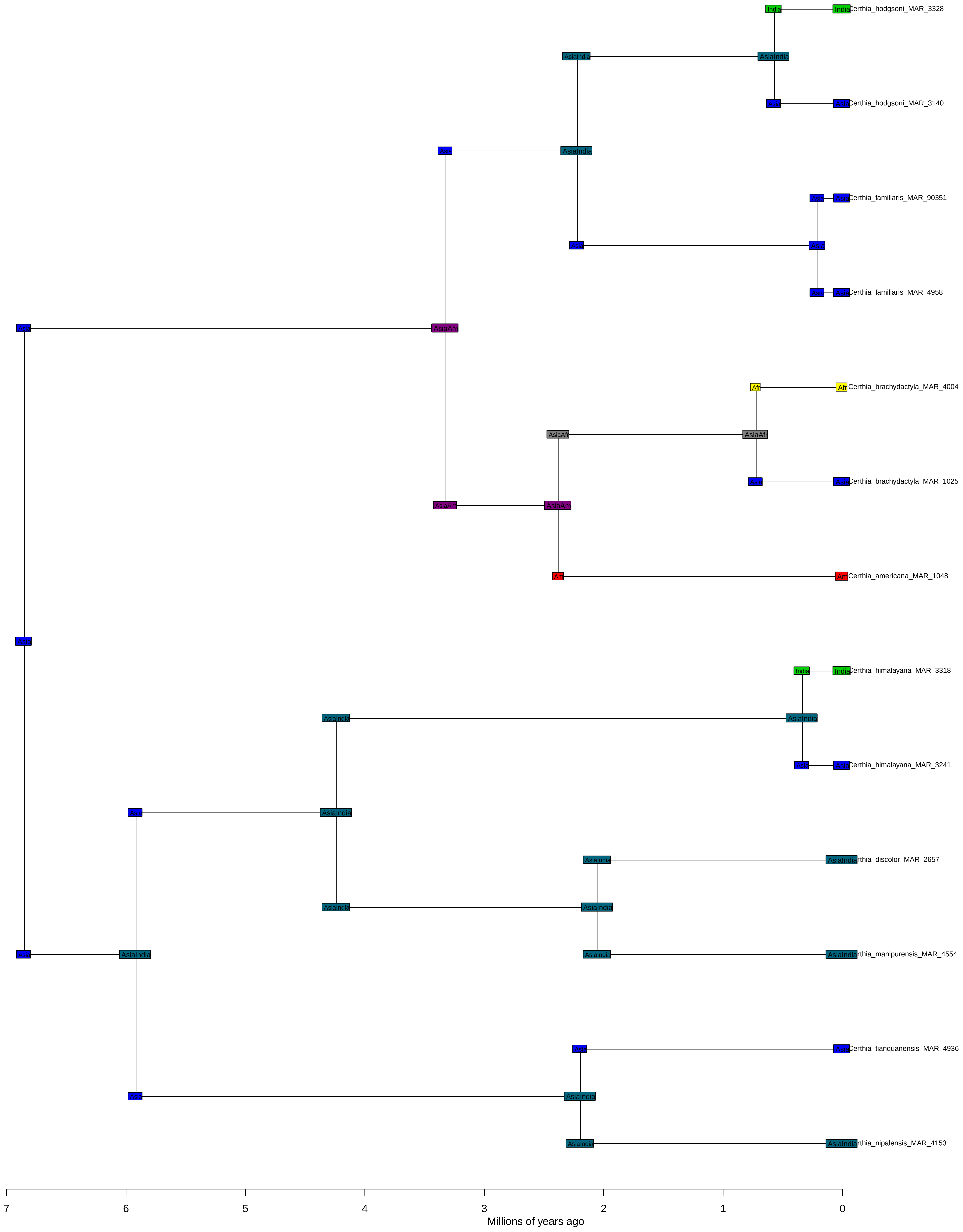

Certhiidae BioGeoBEARS DEC
ancstates: global optim, 2 areas max. d=0.1046; e=0; j=0; LnL=−23.68

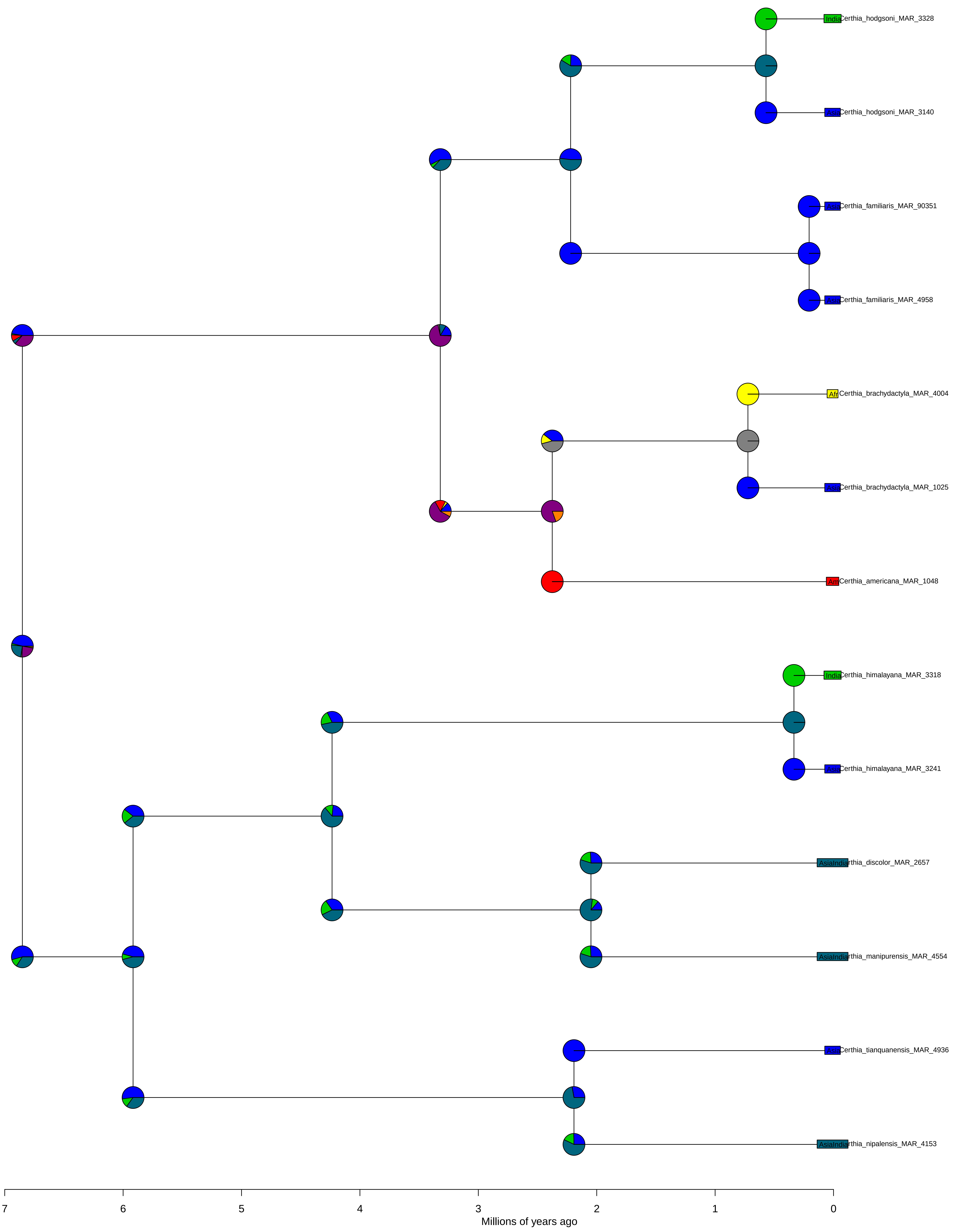

Aegithalidae BioGeoBEARS DEC
ancstates: global optim, 2 areas max. d=0.0344; e=0.0097; j=0; LnL=−21.85

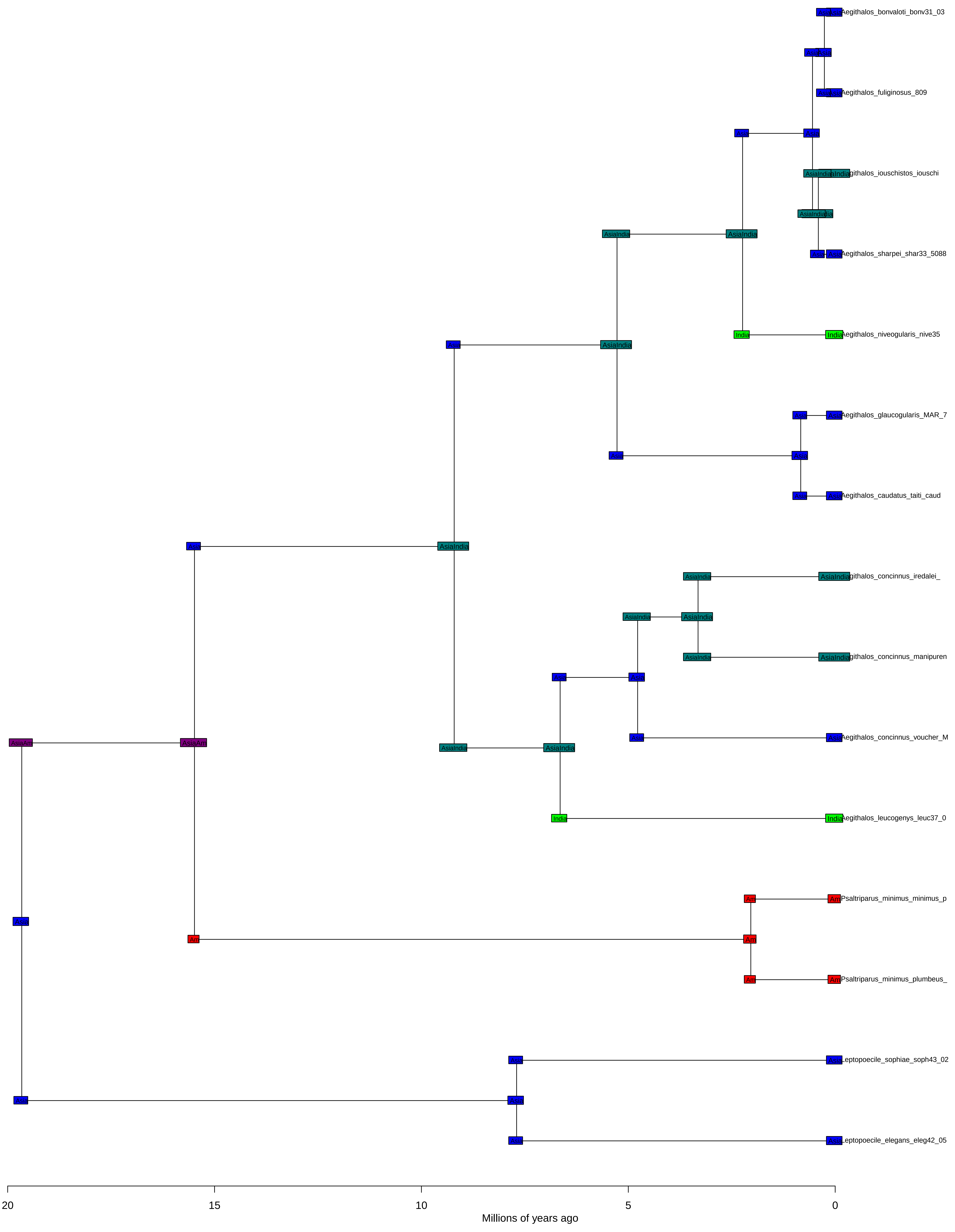

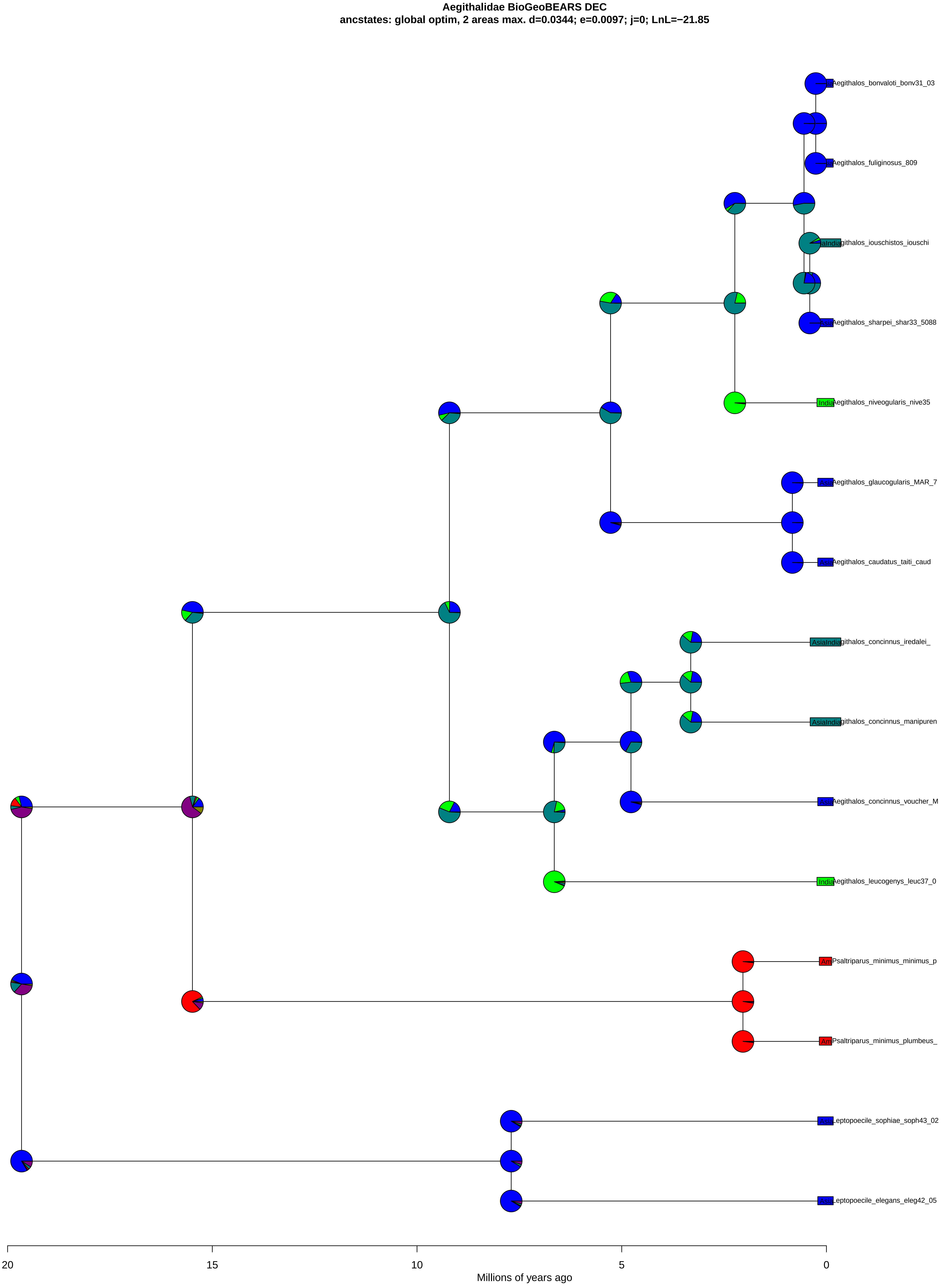
Aegithalidae BioGeoBEARS DEC
ancstates: global optim, 2 areas max. d=0.0344; e=0.0097; j=0; LnL=−21.85
Aegithalos_bonvaloti_bonv31_03
Aegithalos_fuliginosus_809
Aegithalos_iouschistos_iouschi
Aegithalos_sharpei_shar33_5088
India
Aegithalos_niveogularis_nive35
Aegithalos_glaucogularis_MAR_7
Aegithalos_caudatus_taiti_caud
AsiaIndia
Aegithalos_concinnus_iredalei_
Aegithalos_concinnus_manipuren
Aegithalos_concinnus_voucher_M
Aegithalos_leucogenys_leuc37_0
Am
Psaltriparus_minimus_minimus_p
Psaltriparus_minimus_plumbeus_
Leptopoecile_sophiae_soph43_02
Leptopoecile_elegans_eleg42_05
20
15
10
5
0
Millions of years ago

Paridae BioGeoBEARS DEC+J
ancstates: global optim, 2 areas max. d=0.0493; e=0; j=0.0566; LnL=−19.27

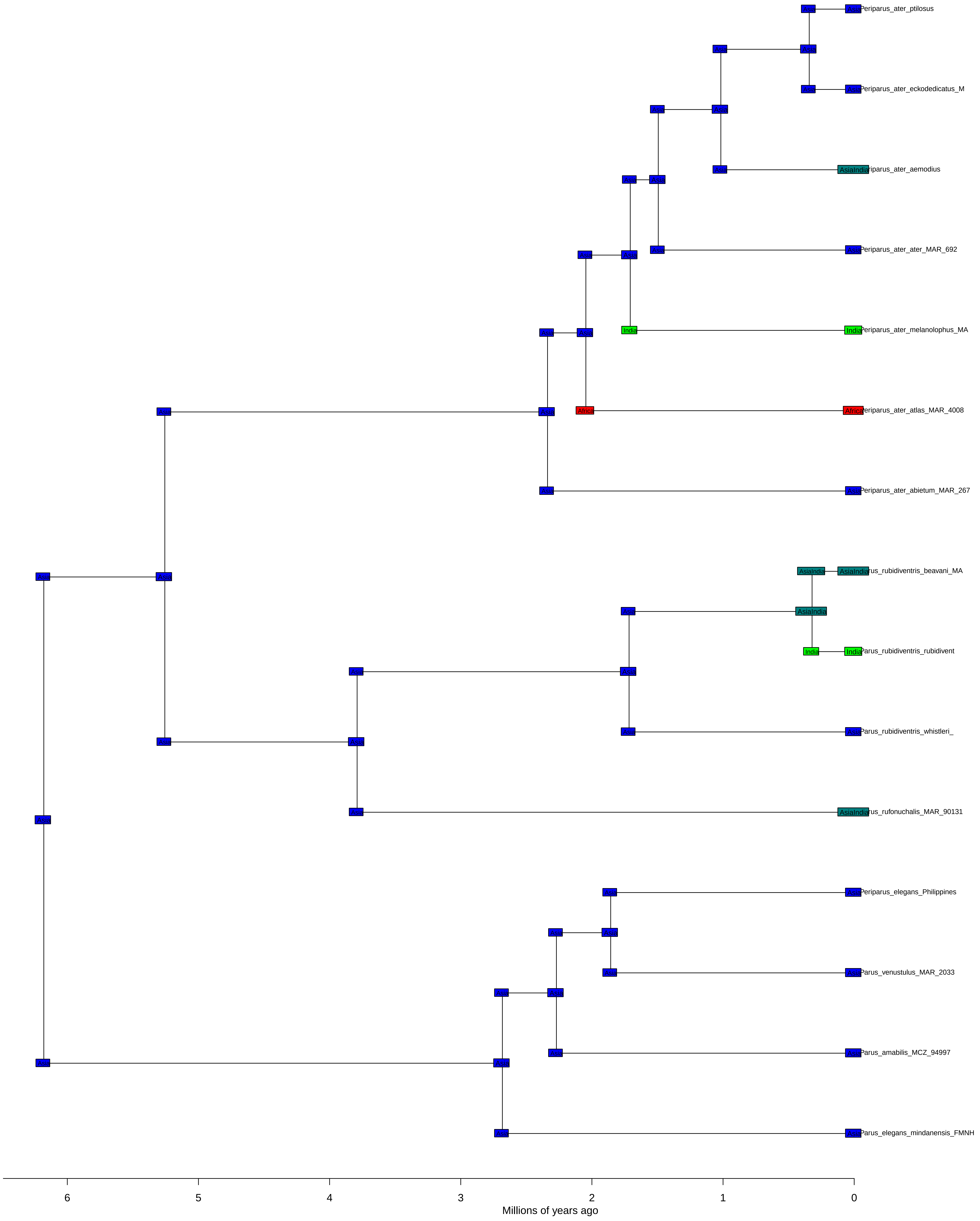

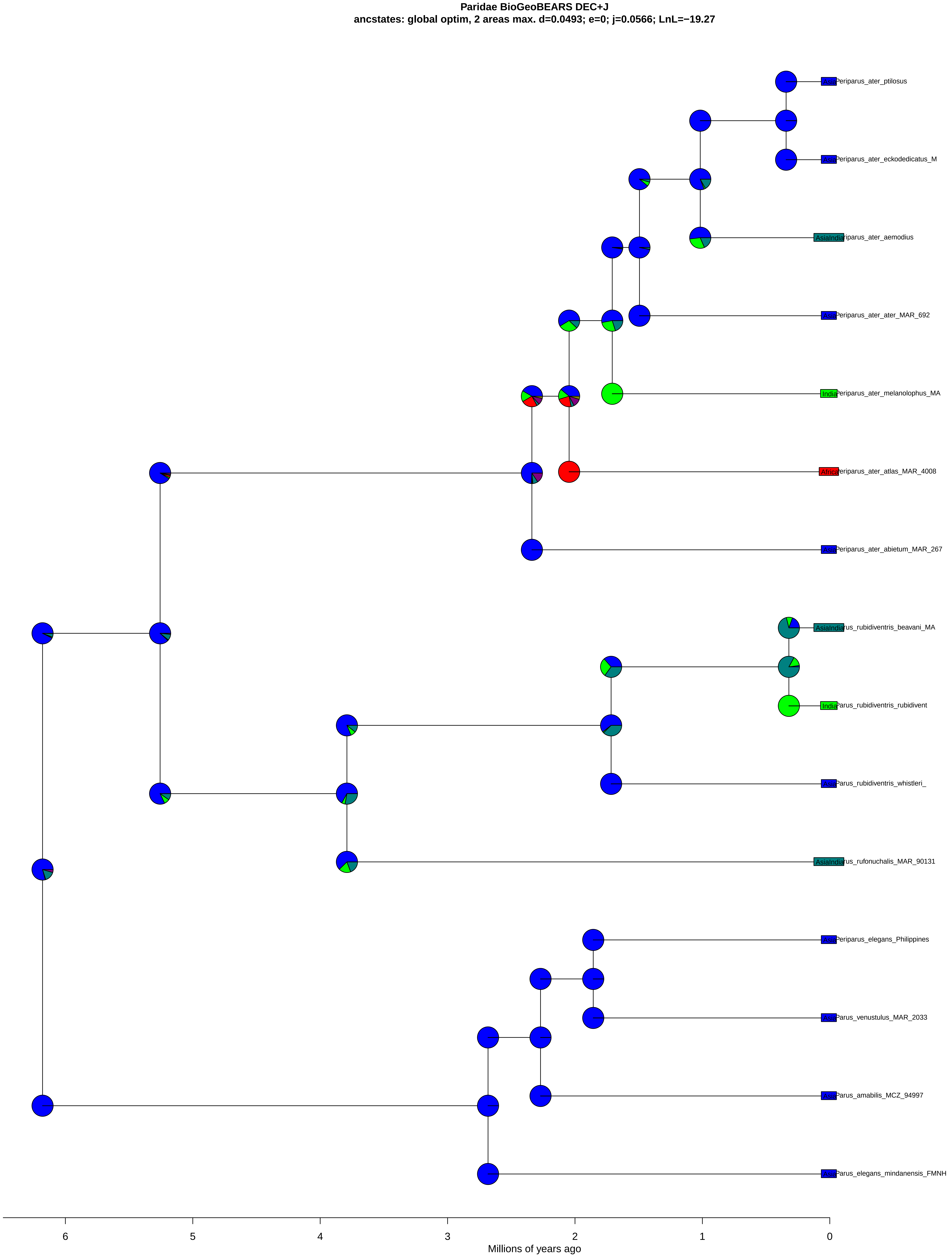

Paridae BioGeoBEARS DEC+J
ancstates: global optim, 2 areas max. d=0.0493; e=0; j=0.0566; LnL=−19.27
Periparus_ater_ptilosus
Periparus_ater_eckodedicatus_M
AsiaIndia
riparus_ater_aemodius
Periparus_ater_ater_MAR_692
India
Periparus_ater_melanolophus_MA
Africa
eriparus_ater_atlas_MAR_4008
Periparus_ater_abietum_MAR_267
AsiaIndia
rus_rubidiventris_beavani_MA
India
Parus_rubidiventris_rubidivent
Parus_rubidiventris_whistleri_
AsiaIndia
rus_rufonuchalis_MAR_90131
Periparus_elegans_Philippines
Parus_venustulus_MAR_2033
Parus_amabilis_MCZ_94997
Parus_elegans_mindanensis_FMNH
6
5
4
3
2
1
0
Millions of years ago

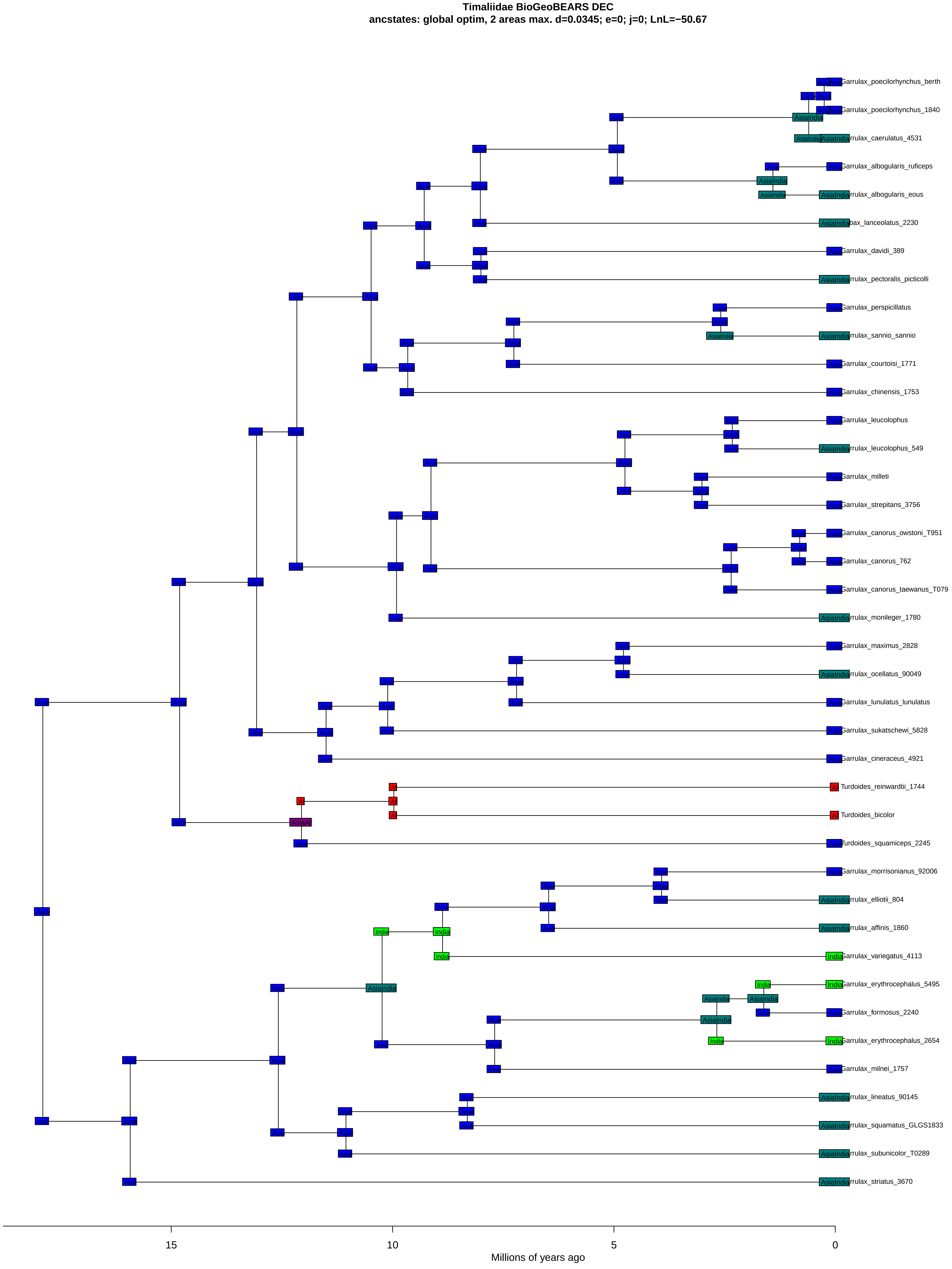

Timaliidae BioGeoBEARS DEC
ancstates: global optim, 2 areas max. d=0.0345; e=0; j=0; LnL=−50.67
Garrulax_poecilorhynchus_berth
Garrulax_poecilorhynchus_1840
rrulax_caerulatus_4531
Garrulax_albogularis_ruficeps
rrulax_albogularis_eous
bax_lanceolatus_2230
Garrulax_davidi_389
rrulax_pectoralis_picticolli
Garrulax_perspicillatus
rrulax_sannio_sannio
Garrulax_courtoisi_1771
Garrulax_chinensis_1753
Garrulax_leucolophus
rrulax_leucolophus_549
Garrulax_milleti
Garrulax_strepitans_3756
Garrulax_canorus_owstoni_T951
Garrulax_canorus_762
Garrulax_canorus_taewanus_T079
rrulax_monileger_1780
Garrulax_maximus_2828
rrulax_ocellatus_90049
Garrulax_lunulatus_lunulatus
Garrulax_sukatschewi_5828
Garrulax_cineraceus_4921
Turdoides_reinwardtii_1744
Turdoides_bicolor
Turdoides_squamiceps_2245
Garrulax_morrisonianus_92006
rrulax_elliotii_804
rrulax_affinis_1860
Garrulax_variegatus_4113
Garrulax_erythrocephalus_5495
Garrulax_formosus_2240
Garrulax_erythrocephalus_2654
Garrulax_milnei_1757
rrulax_lineatus_90145
rrulax_squamatus_GLGS1833
rrulax_subunicolor_T0289
rrulax_striatus_3670
AsiaIndia
India
15
10
5
0
Millions of years ago

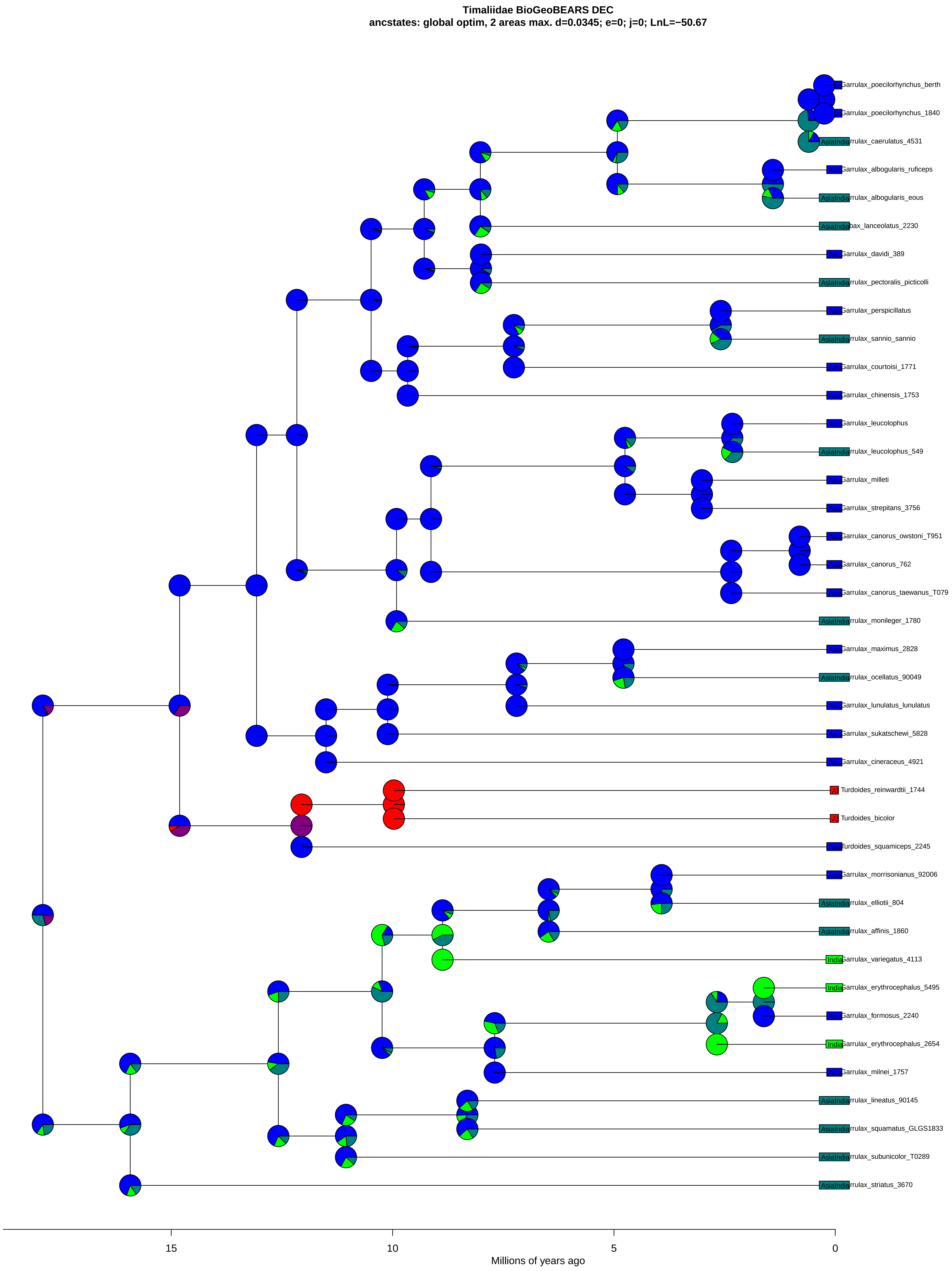
Timaliidae BioGeoBEARS DEC
ancstates: global optim, 2 areas max. d=0.0345; e=0; j=0; LnL=−50.67
Garrulax_poecilorhynchus_berth
Garrulax_poecilorhynchus_1840
AsiaIndia rrulax_caerulatus_4531
Garrulax_albogularis_ruficeps
AsiaIndia rrulax_albogularis_eous
AsiaIndia bax_lanceolatus_2230
Garrulax_davidi_389
AsiaIndia rrulax_pectoralis_picticolli
Garrulax_perspicillatus
AsiaIndia rrulax_sannio_sannio
Garrulax_courtoisi_1771
Garrulax_chinensis_1753
Garrulax_leucolophus
AsiaIndia rrulax_leucolophus_549
Garrulax_milleti
Garrulax_strepitans_3756
Garrulax_canorus_owstoni_T951
Garrulax_canorus_762
Garrulax_canorus_taewanus_T079
AsiaIndia rrulax_monileger_1780
Garrulax_maximus_2828
AsiaIndia rrulax_ocellatus_90049
Garrulax_lunulatus_lunulatus
Garrulax_sukatschewi_5828
Garrulax_cineraceus_4921
Turdoides_reinwardtii_1744
Turdoides_bicolor
Turdoides_squamiceps_2245
Garrulax_morrisonianus_92006
AsiaIndia rrulax_elliotii_804
AsiaIndia rrulax_affinis_1860
India Garrulax_variegatus_4113
India Garrulax_erythrocephalus_5495
Garrulax_formosus_2240
India Garrulax_erythrocephalus_2654
Garrulax_milnei_1757
AsiaIndia rrulax_lineatus_90145
AsiaIndia rrulax_squamatus_GLGS1833
AsiaIndia rrulax_subunicolor_T0289
AsiaIndia rrulax_striatus_3670
15
10
5
0
Millions of years ago

Pyrrhula BioGeoBEARS DEC
ancstates: global optim, 2 areas max. d=0.1031; e=0; j=0; LnL=−18.59

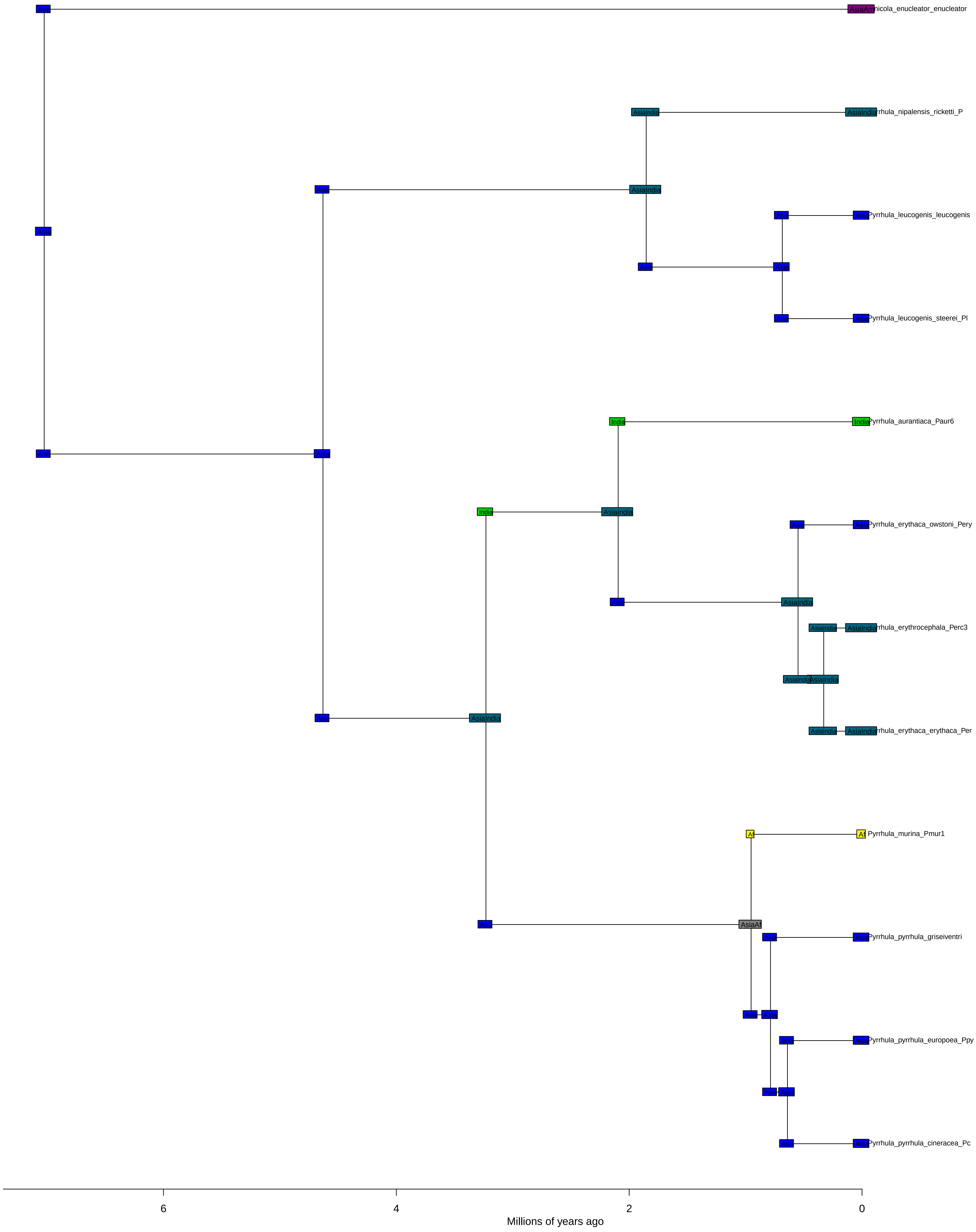

Pyrrhula BioGeoBEARS DEC
ancstates: global optim, 2 areas max. d=0.1031; e=0; j=0; LnL=−18.59

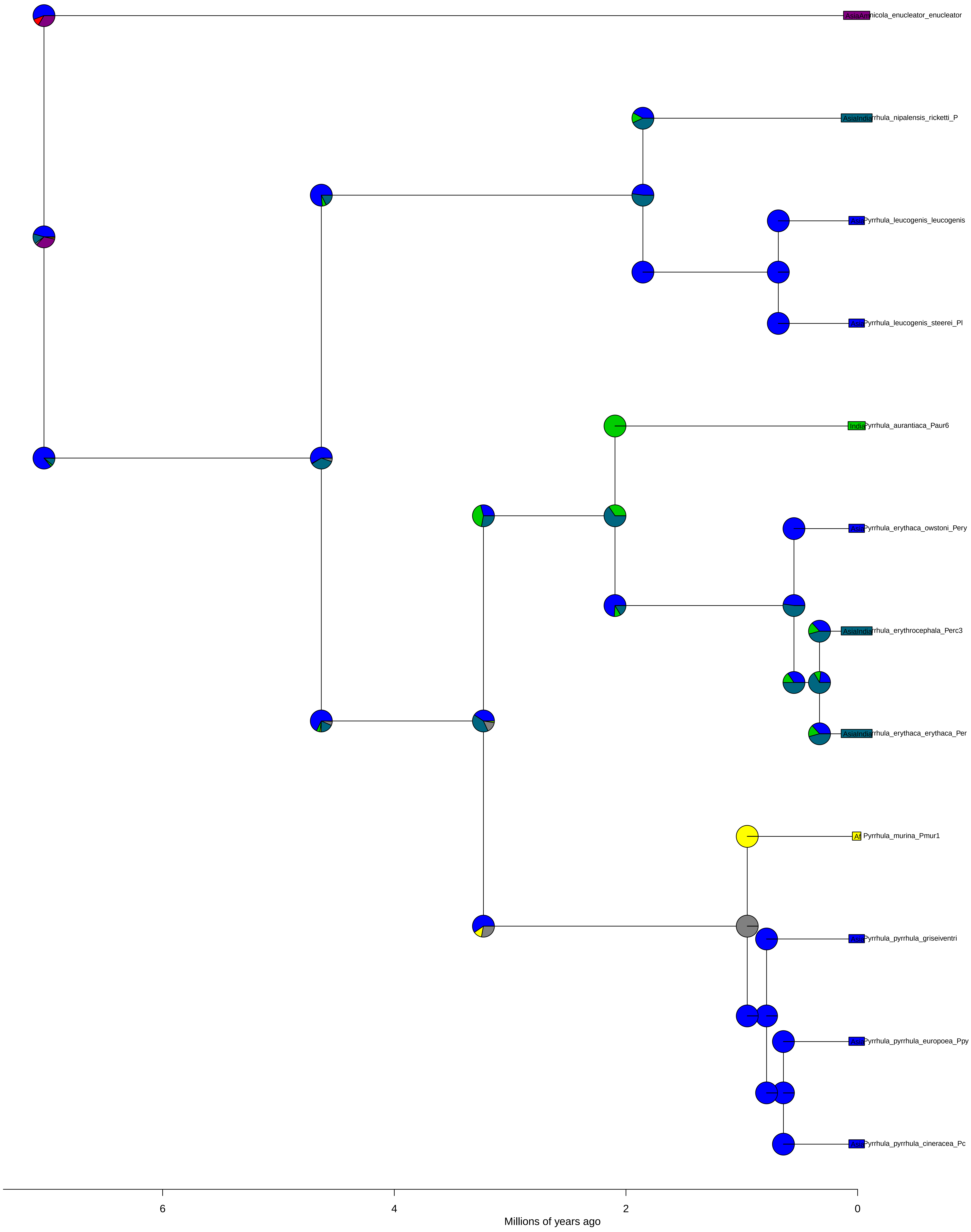

Psittacula BioGeoBEARS DEC+J
ancstates: global optim, 2 areas max. d=0.0707; e=0; j=0.1075; LnL=−28.60

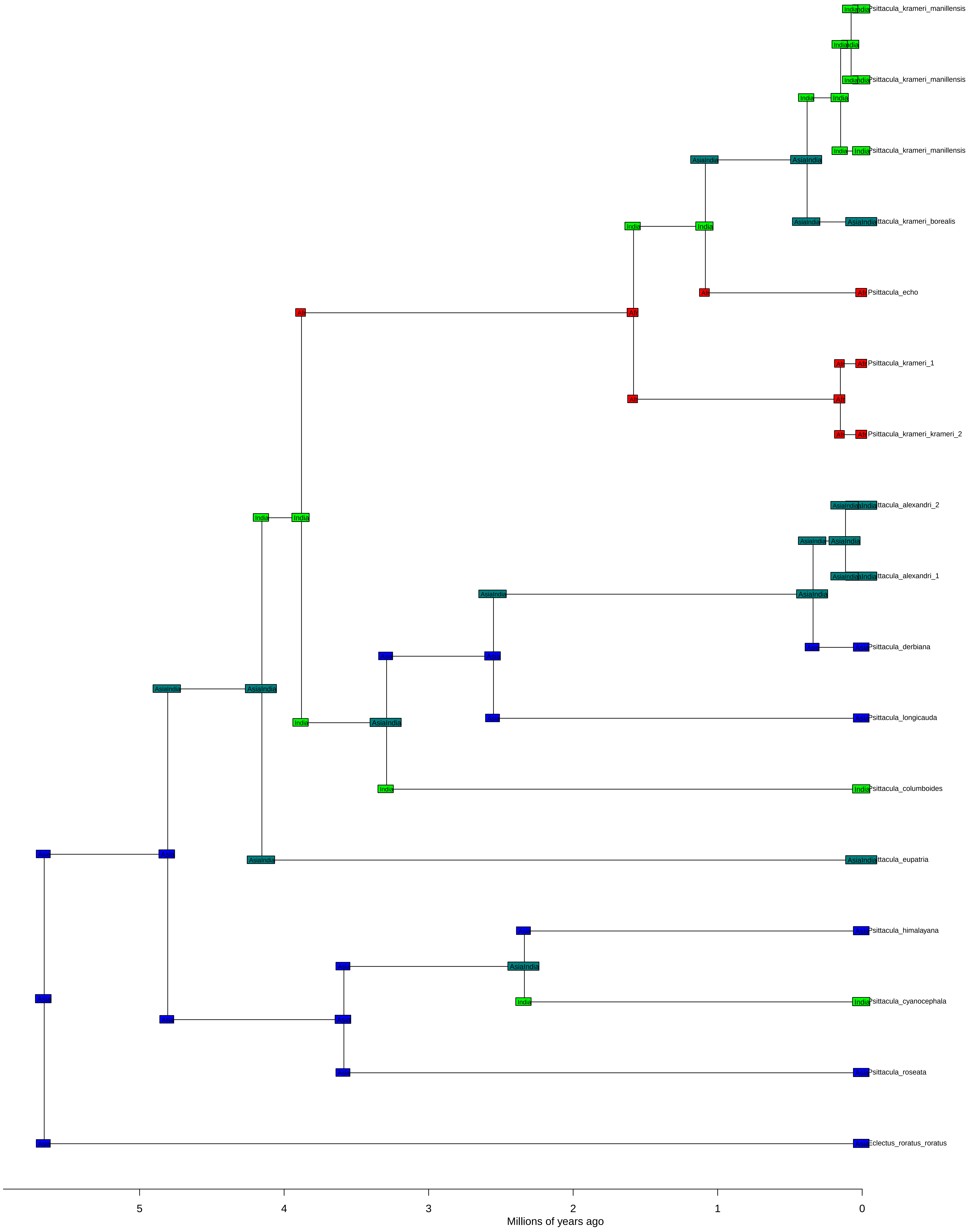

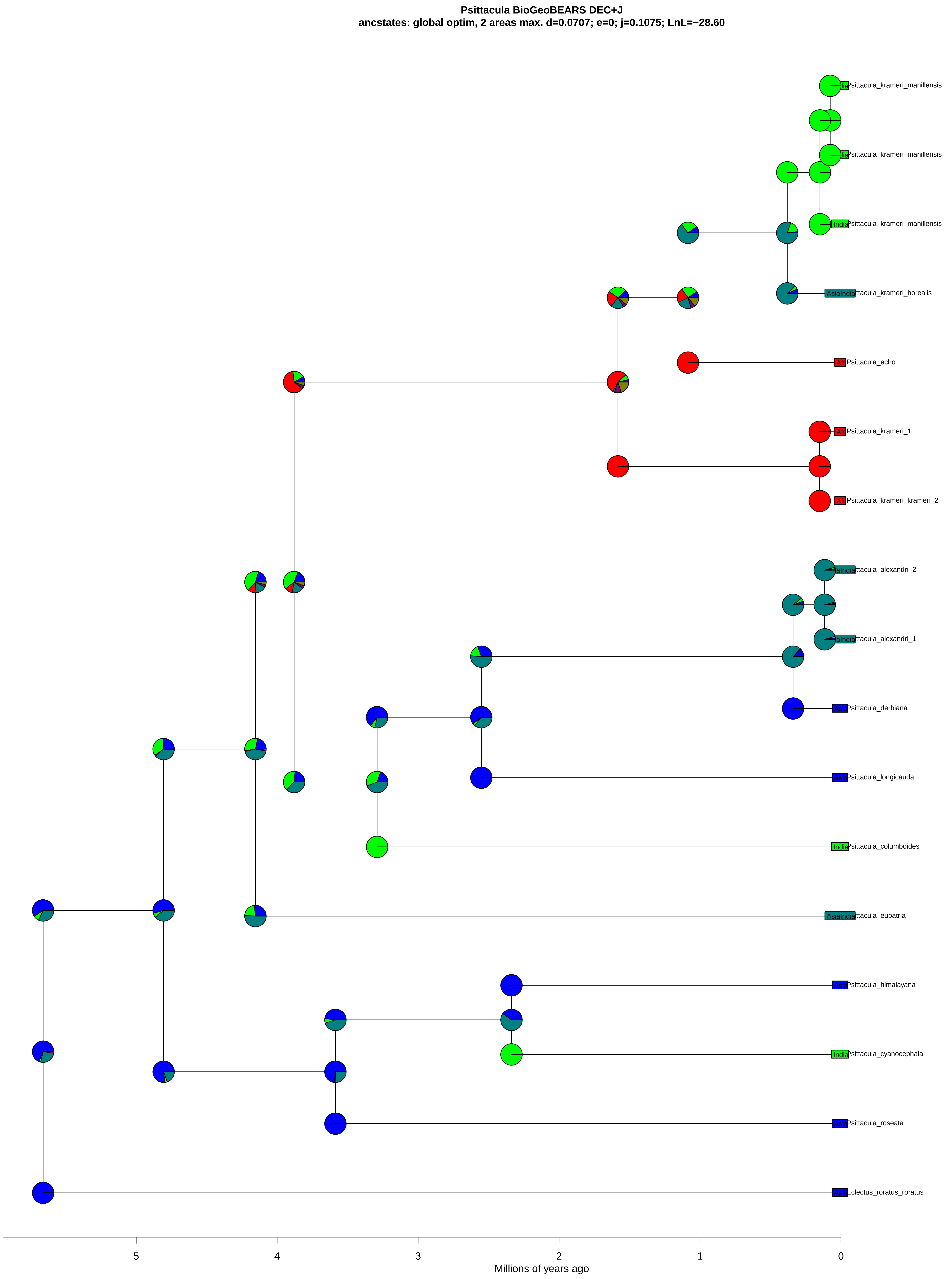

Psittacula BioGeoBEARS DEC+J
ancstates: global optim, 2 areas max. d=0.0707; e=0; j=0.1075; LnL=−28.60
Psittacula_krameri_manillensis
Psittacula_krameri_manillensis
India
Psittacula_krameri_manillensis
AsiaIndia
ttacula_krameri_borealis
Af
Psittacula_echo
Af
Psittacula_krameri_1
Af
Psittacula_krameri_krameri_2
ttacula_alexandri_2
ttacula_alexandri_1
Psittacula_derbiana
Psittacula_longicauda
India
Psittacula_columboides
AsiaIndia
ttacula_eupatria
Psittacula_himalayana
India
Psittacula_cyanocephala
Psittacula_roseata
Eclectus_roratus_roratus
5
4
3
2
1
0
Millions of years ago

Nisaetus (Accipitridae) BioGeoBEARS DEC
ancstates: global optim, 2 areas max. d=0.3846; e=0; j=0; LnL=−11.51

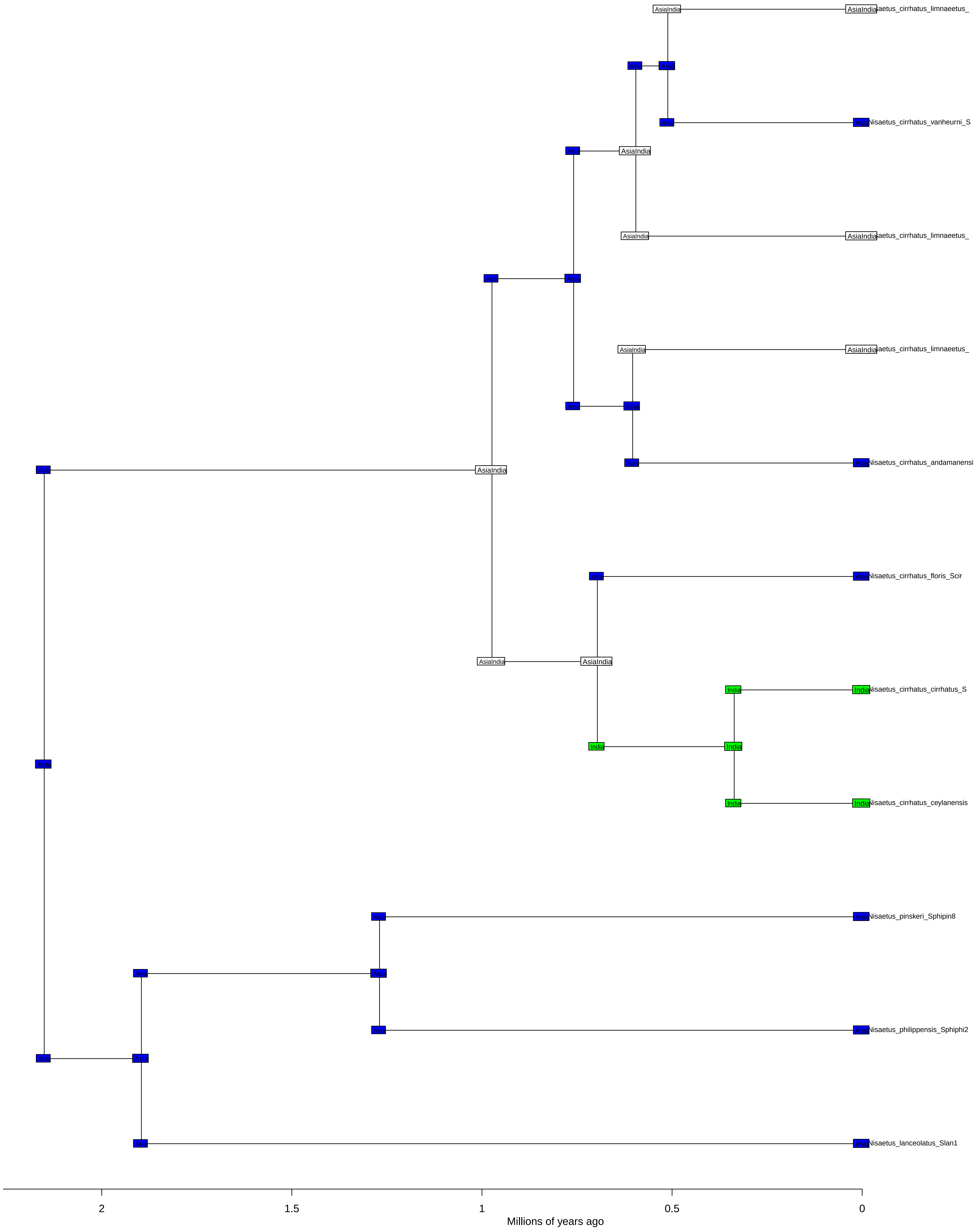

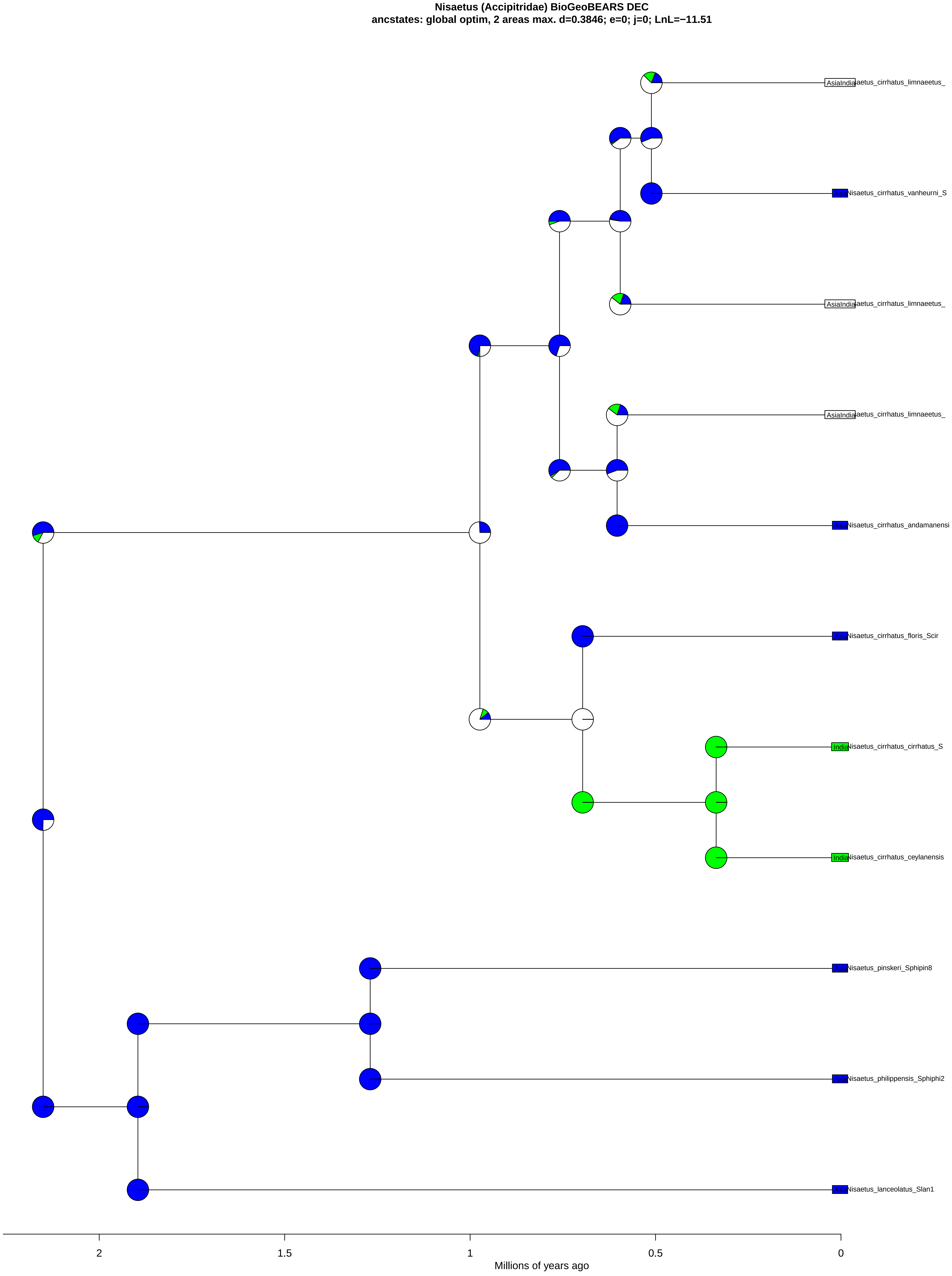
Nisaetus (Accipitridae) BioGeoBEARS DEC
ancstates: global optim, 2 areas max. d=0.3846; e=0; j=0; LnL=−11.51
AsiaIndia
aetus_cirrhatus_limnaeetus_
Nisaetus_cirrhatus_vanheurni_S
AsiaIndia
aetus_cirrhatus_limnaeetus_
AsiaIndia
aetus_cirrhatus_limnaeetus_
Nisaetus_cirrhatus_andamanensi
Nisaetus_cirrhatus_floris_Scir
India
Nisaetus_cirrhatus_cirrhatus_S
India
Nisaetus_cirrhatus_ceylanensis
Nisaetus_pinskeri_Sphipin8
Nisaetus_philippensis_Sphiphi2
Nisaetus_lanceolatus_Slan1
2
1.5
1
0.5
0
Millions of years ago

Sciuridae, Pteromyini BioGeoBEARS DEC+J
ancstates: global optim, 2 areas max. d=0.0048; e=0; j=0.1439; LnL=−32.24

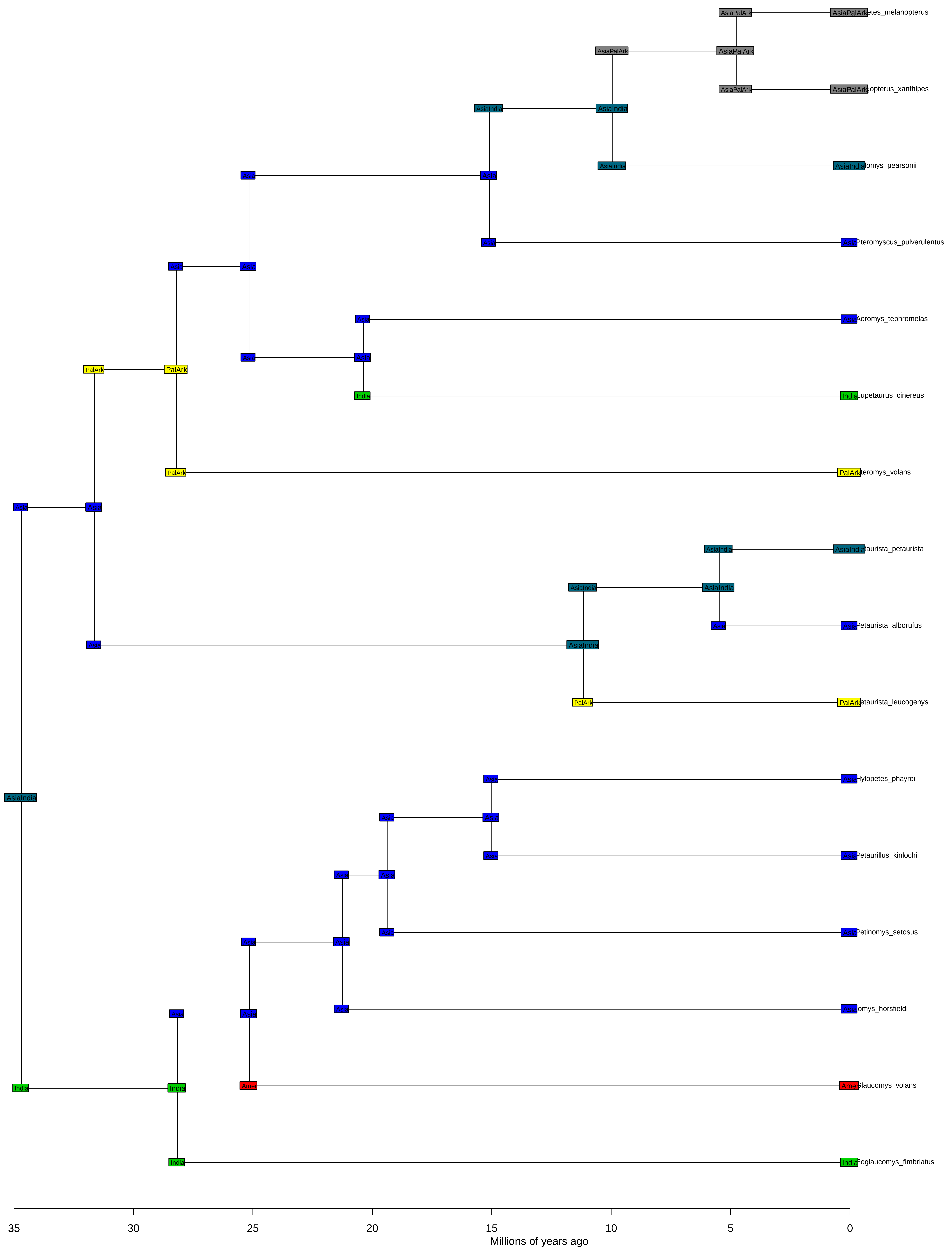

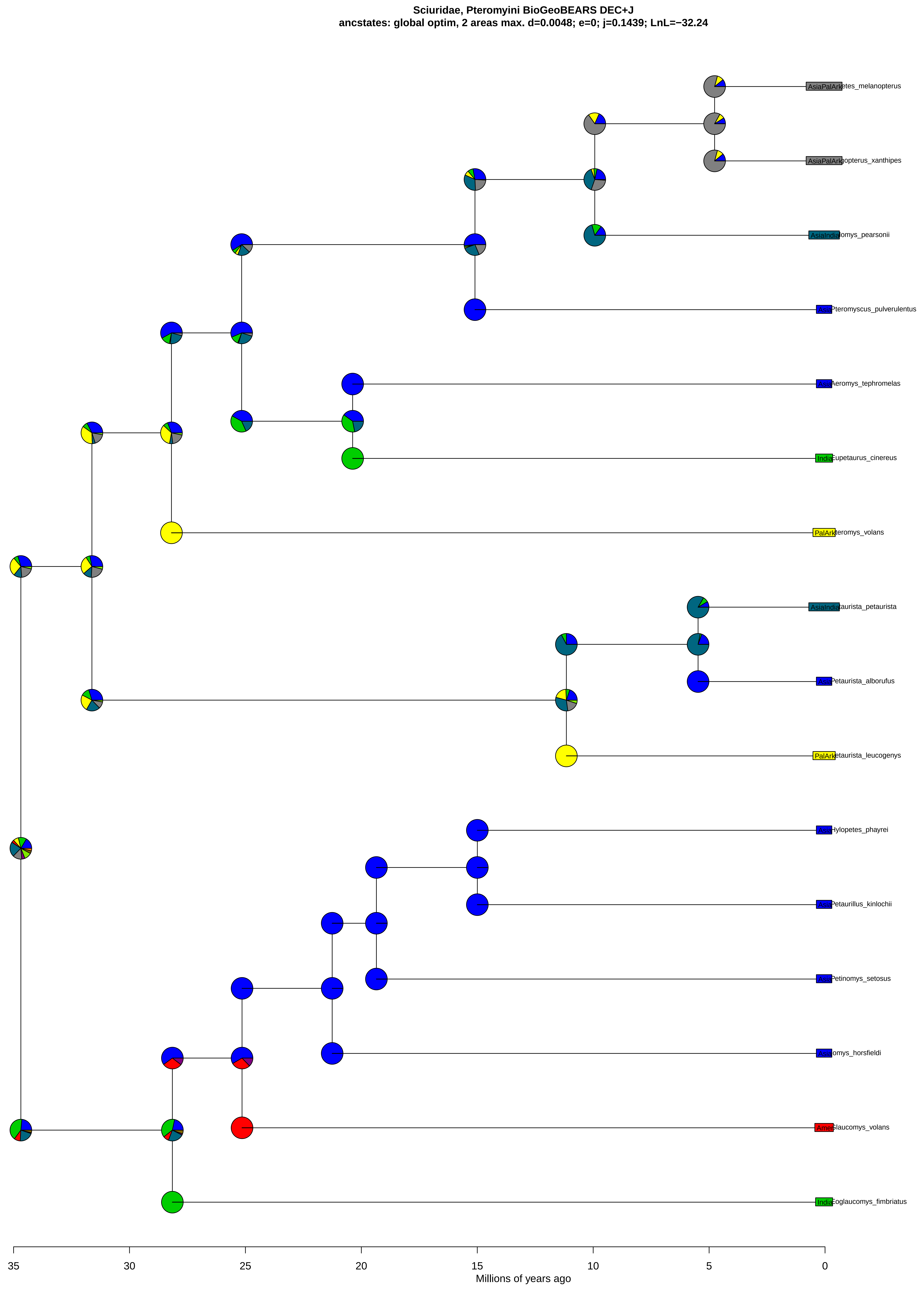
Sciuridae, Pteromyini BioGeoBEARS DEC+J
ancstates: global optim, 2 areas max. d=0.0048; e=0; j=0.1439; LnL=−32.24
AsiaPalArketes_melanopterus
AsiaPalArkopterus_xanthipes
AsiaIndiaomys_pearsonii
AsiaPteromyscus_pulverulentus
AsiaAeromys_tephromelas
IndiaEupetaurus_cinereus
PalArkteromys_volans
AsiaIndiaaurista_petaurista
AsiaPetaurista_alborufus
PalArkPetaurista_leucogenys
AsiaHylopetes_phayrei
AsiaPetaurillus_kinlochii
AsiaPetinomys_setosus
Asiaomys_horsfieldi
AmerGlaucomys_volans
IndiaEoglaucomys_fimbriatus
35
30
25
20
15
10
5
0
Millions of years ago

Ruminantia BioGeoBEARS DEC+J

ancstates: global optim, 3 areas max. d=0.0027; e=0; j=0.022; LnL=−142.23

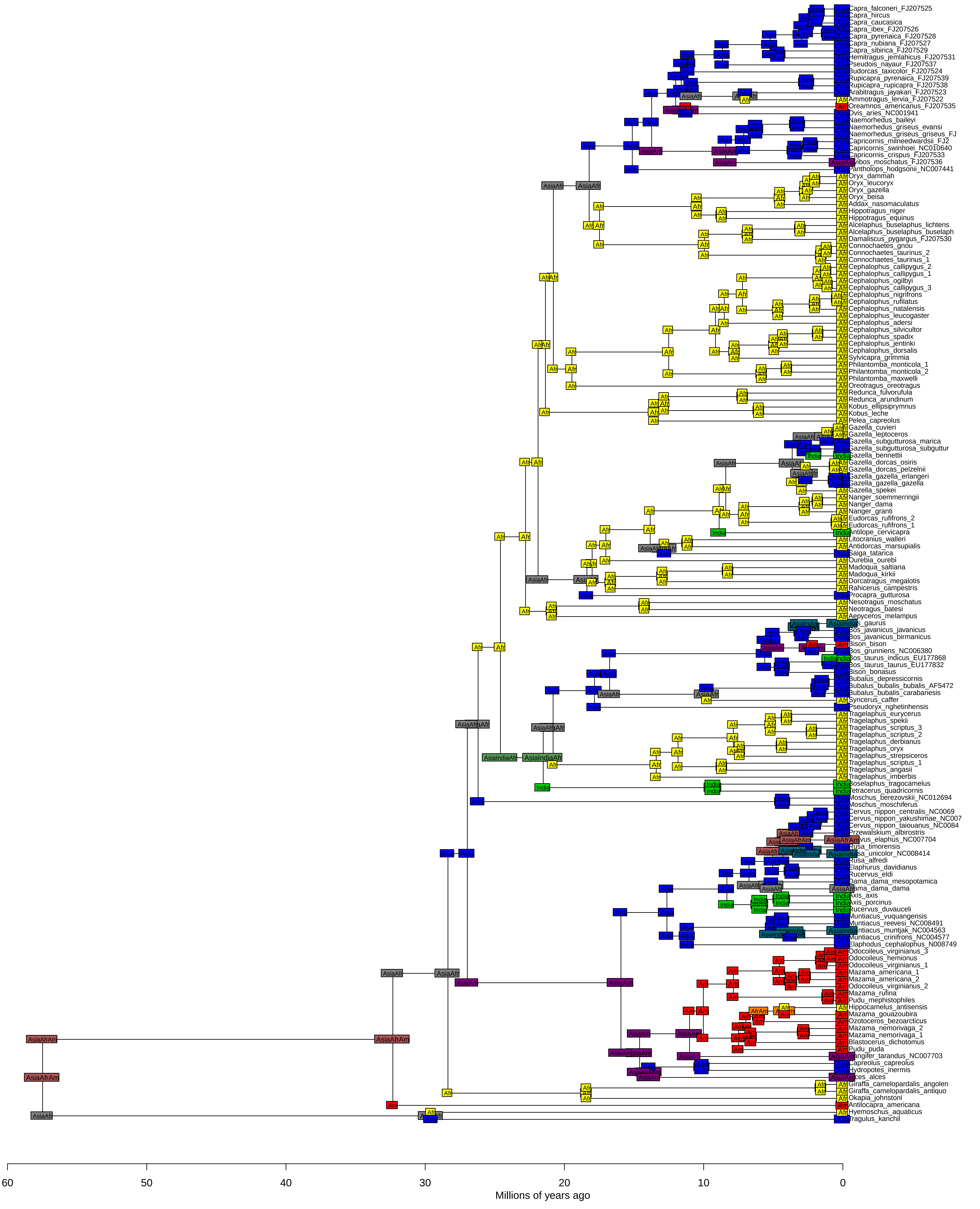

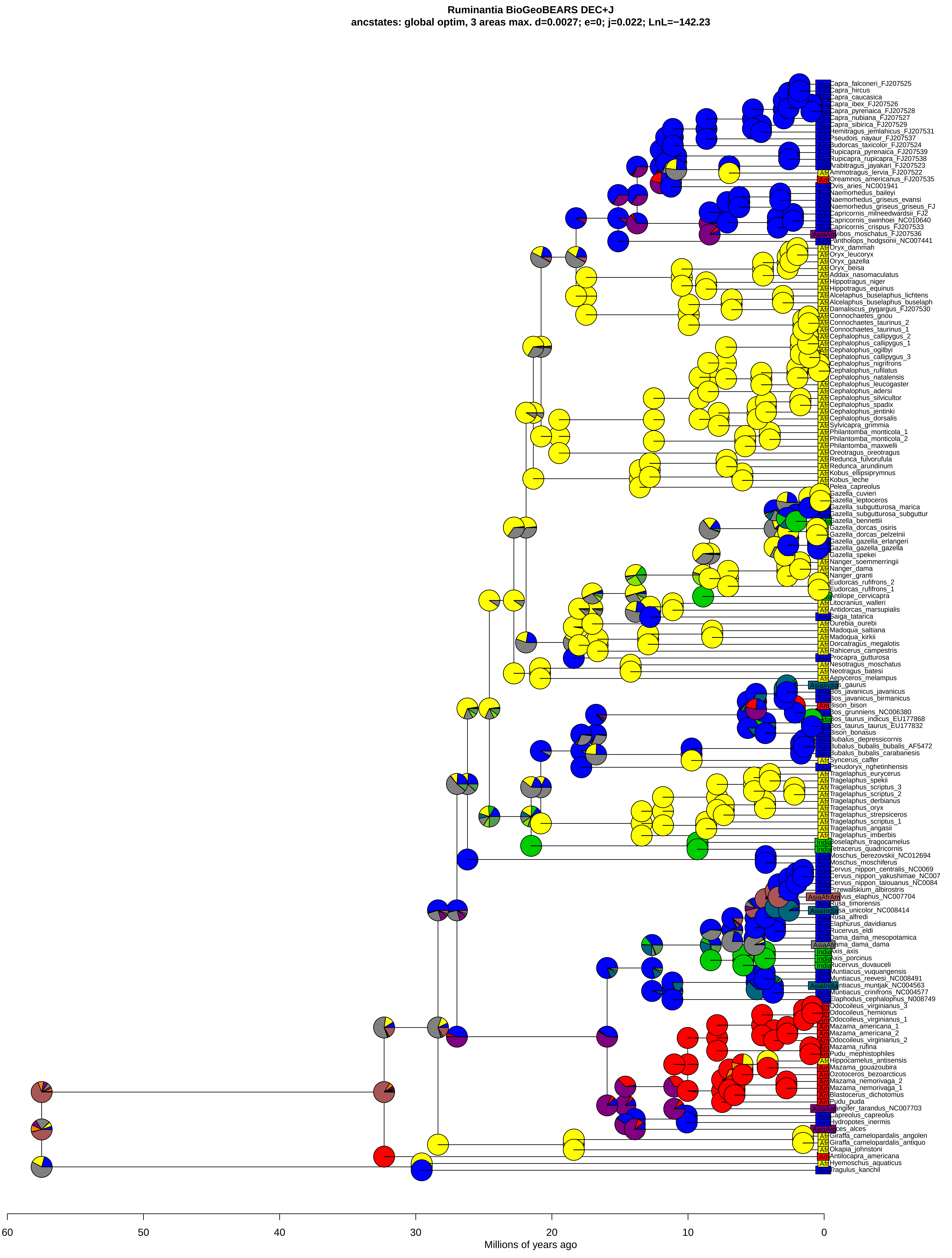
Ruminantia BioGeoBEARS DEC+J
ancstates: global optim, 3 areas max. d=0.0027; e=0; j=0.022; LnL=−142.23
Capra_falconeri_FJ207525
Capra_hircus
Capra_caucasica
Capra_ibex_FJ207526
Capra_pyrenaica_FJ207528
Capra_nubiana_FJ207527
Capra_sibirica_FJ207529
Hemitragus_jemlahicus_FJ207531
Pseudois_nayaur_FJ207537
Budorcas_taxicolor_FJ207524
Rupicapra_pyrenaica_FJ207539
Rupicapra_rupicapra_FJ207538
Arabitragus_jayakari_FJ207523
Ammotragus_lervia_FJ207522
Oreamnos_americanus_FJ207535
Ovis_aries_NC001941
Naemorhedus_baileyi
Naemorhedus_griseus_evansi
Naemorhedus_griseus_griseus_FJ
Capricornis_milneedwardsii_FJ2
Capricornis_swinhoei_NC010640
Capricornis_crispus_FJ207533
Pantholops_hodgsonii_NC007441
Oryx_dammah
Oryx_leucoryx
Oryx_gazella
Oryx_beisa
Addax_nasomaculatus
Hippotragus_niger
Hippotragus_equinus
Alcelaphus_buselaphus_lichtens
Alcelaphus_buselaphus_buselaph
Damaliscus_pygargus_FJ207530
Connochaetes_gnou
Connochaetes_taurinus_2
Connochaetes_taurinus_1
Cephalophus_callipygus_2
Cephalophus_callipygus_1
Cephalophus_ogilbyi
Cephalophus_callipygus_3
Cephalophus_nigrifrons
Cephalophus_rufilatus
Cephalophus_natalensis
Cephalophus_leucogaster
Cephalophus_adersi
Cephalophus_silvicultor
Cephalophus_spadix
Cephalophus_jentinki
Cephalophus_dorsalis
Sylvicapra_grimmia
Philantomba_monticola_1
Philantomba_monticola_2
Philantomba_maxwelli
Oreotragus_oreotragus
Redunca_fulvorufula
Redunca_arundinum
Kobus_ellipsiprymnus
Kobus_leche
Pelea_capreolus
Gazella_cuvieri
Gazella_leptoceros
Gazella_subgutturosa_marica
Gazella_subgutturosa_subguttur
Gazella_bennettii
Gazella_dorcas_osiris
Gazella_dorcas_pelzelnii
Gazella_gazella_erlangeri
Gazella_gazella_gazella
Gazella_spekei
Nanger_soemmerringii
Nanger_dama
Nanger_granti
Eudorcas_rufifrons_2
Eudorcas_rufifrons_1
Antilope_cervicapra
Litocranius_walleri
Antidorcas_marsupialis
Saiga_tatarica
Ourebia_ourebi
Madoqua_saltiana
Madoqua_kirkii
Dorcatragus_megalotis
Rahicerus_campestris
Procapra_gutturosa
Nesotragus_moschatus
Neotragus_batesi
Aepyceros_melampus
Bos_javanicus_javanicus
Bos_javanicus_birmanicus
Bison_bison
Bos_grunniens_NC006380
Bos_taurus_indicus_EU177868
Bos_taurus_taurus_EU177832
Bison_bonasus
Bubalus_depressicornis
Bubalus_bubalis_bubalis_AF5472
Bubalus_bubalis_carabanesis
Syncerus_caffer
Pseudoryx_nghetinhensis
Tragelaphus_eurycerus
Tragelaphus_spekii
Tragelaphus_scriptus_3
Tragelaphus_scriptus_2
Tragelaphus_derbianus
Tragelaphus_oryx
Tragelaphus_strepsiceros
Tragelaphus_scriptus_1
Tragelaphus_angasii
Tragelaphus_imberbis
Boselaphus_tragocamelus
Tetracerus_quadricornis
Moschus_berezovskii_NC012694
Moschus_moschiferus
Cervus_nippon_centralis_NC0069
Cervus_nippon_yakushimae_NC007
Cervus_nippon_taiouanus_NC0084
Przewalskium_albirostris
Rusa_timorensis
Rusa_alfredi
Elaphurus_davidianus
Rucervus_eldi
Dama_dama_mesopotamica
Axis_axis
Axis_porcinus
Rucervus_duvauceli
Muntiacus_vuquangensis
Muntiacus_reevesi_NC008491
Muntiacus_crinifrons_NC004577
Elaphodus_cephalophus_N008749
Odocoileus_virginianus_3
Odocoileus_hemionus
Odocoileus_virginianus_1
Mazama_americana_1
Mazama_americana_2
Odocoileus_virginianus_2
Mazama_rufina
Pudu_mephistophiles
Hippocamelus_antisensis
Mazama_gouazoubira
Ozotoceros_bezoarcticus
Mazama_nemorivaga_2
Mazama_nemorivaga_1
Blastocerus_dichotomus
Pudu_puda
Capreolus_capreolus
Hydropotes_inermis
Giraffa_camelopardalis_angolen
Giraffa_camelopardalis_antiquo
Okapia_johnstoni
Antilocapra_americana
Hyemoschus_aquaticus
Tragulus_kanchil
60
50
40
30
20
10
0
Millions of years ago

Herpestidae BioGeoBEARS DEC+J
ancstates: global optim, 2 areas max. d=0; e=0; j=0.0555; LnL=−22.79

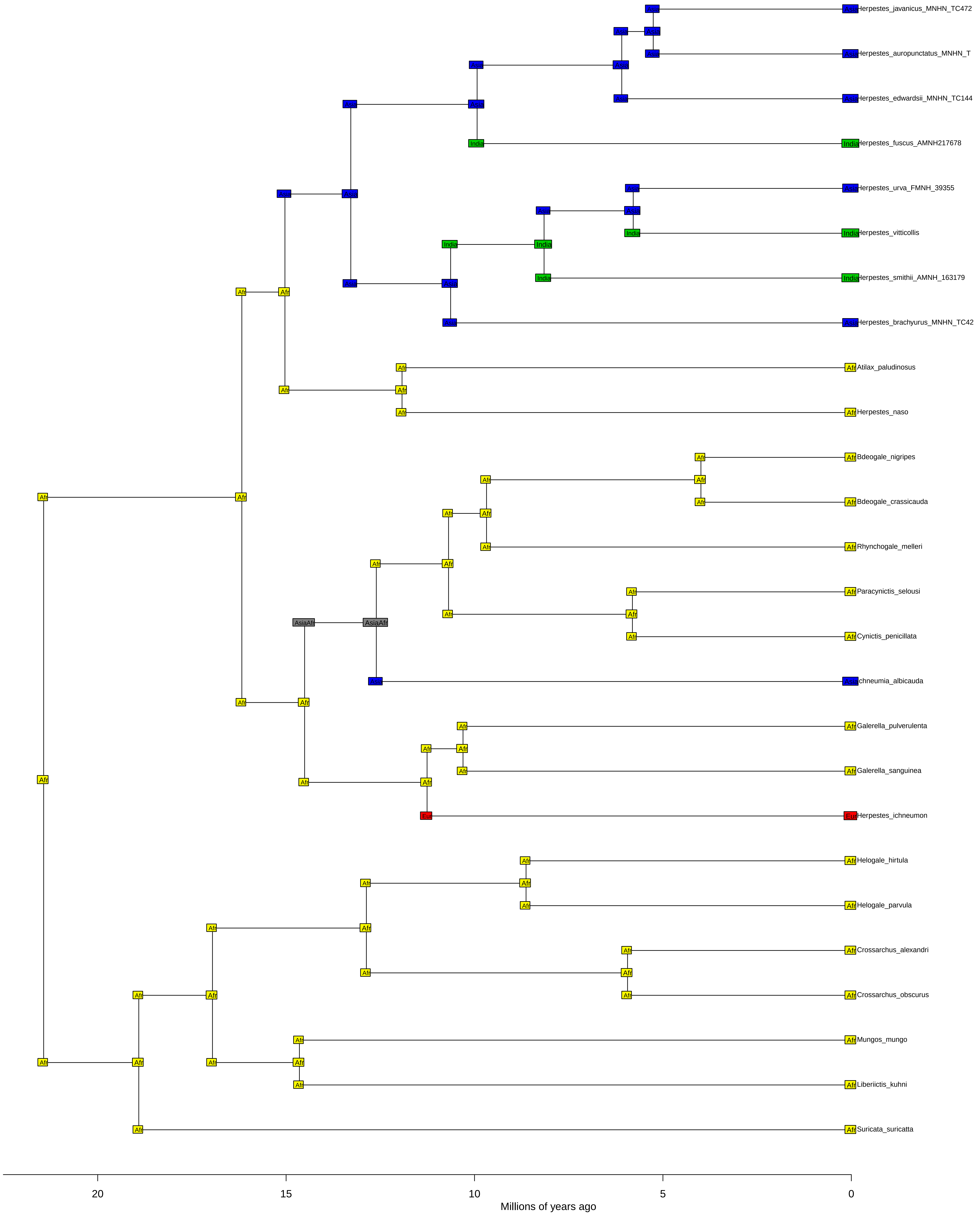

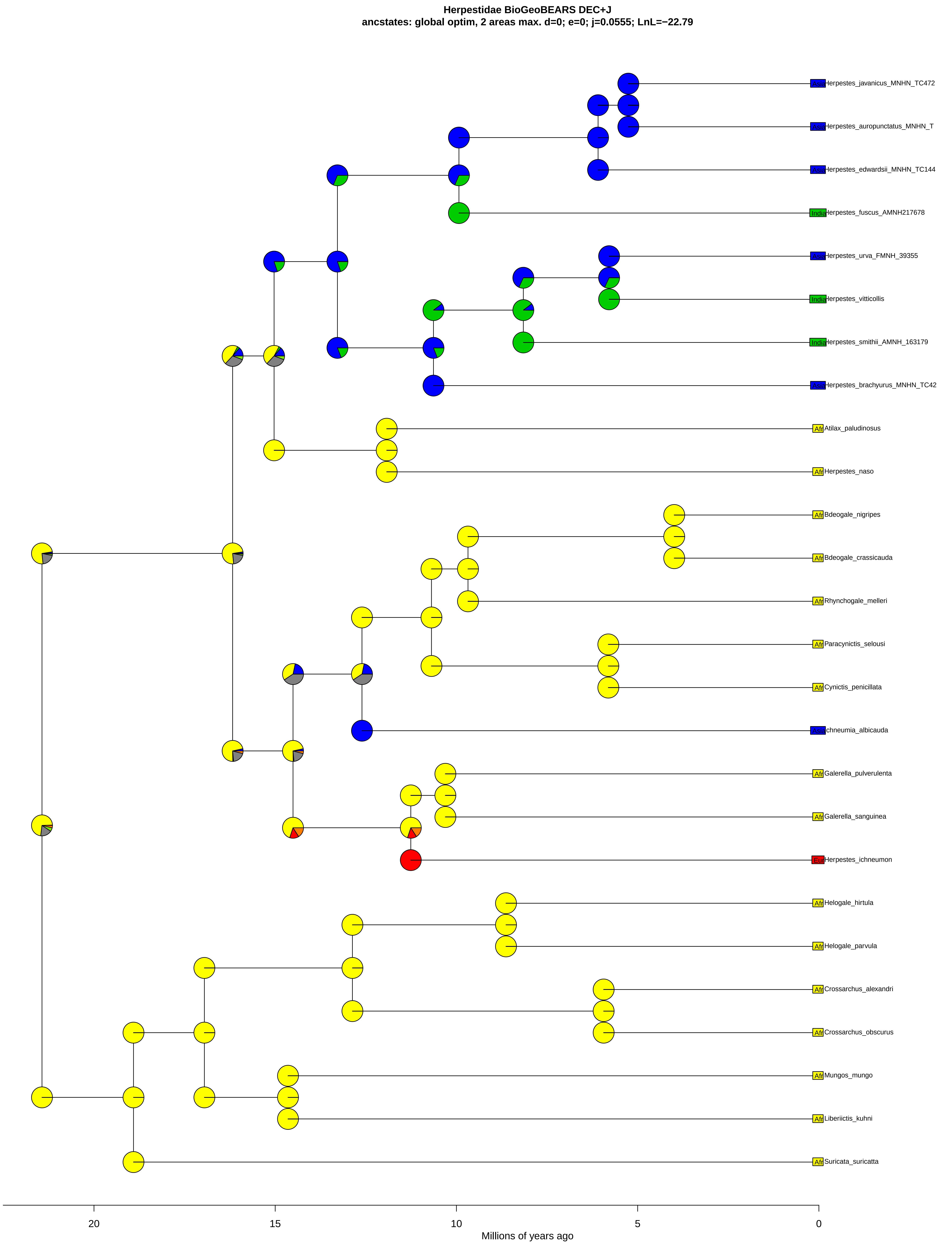
Herpestidae BioGeoBEARS DEC+J
ancstates: global optim, 2 areas max. d=0; e=0; j=0.0555; LnL=−22.79
Herpestes_javanicus_MNHN_TC472
Herpestes_auropunctatus_MNHN_T
Herpestes_edwardsii_MNHN_TC144
India
Herpestes_fuscus_AMNH217678
Herpestes_urva_FMNH_39355
India
Herpestes_vitticollis
India
Herpestes_smithii_AMNH_163179
Herpestes_brachyurus_MNHN_TC42
Afr
Atilax_paludinosus
Afr
Herpestes_naso
Afr
Bdeogale_nigripes
Afr
Bdeogale_crassicauda
Afr
Rhynchogale_melleri
Afr
Paracynictis_selousi
Afr
Cynictis_penicillata
chneumia_albicauda
Afr
Galerella_pulverulenta
Afr
Galerella_sanguinea
Eur
Herpestes_ichneumon
Afr
Helogale_hirtula
Afr
Helogale_parvula
Afr
Crossarchus_alexandri
Afr
Crossarchus_obscurus
Afr
Mungos_mungo
Afr
Liberiictis_kuhni
Afr
Suricata_suricatta
20
15
10
5
0
Millions of years ago

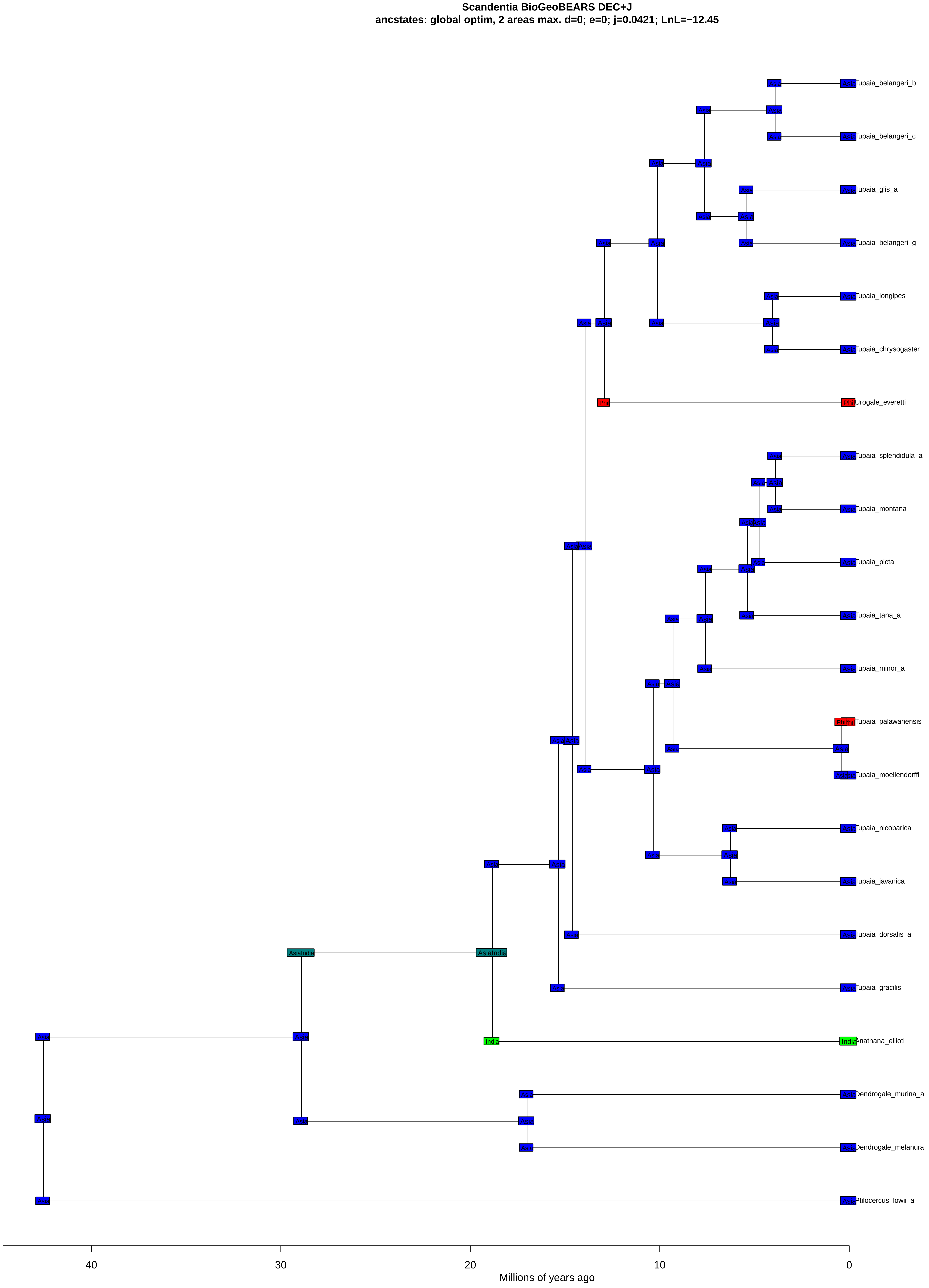
Scandentia BioGeoBEARS DEC+J
ancstates: global optim, 2 areas max. d=0; e=0; j=0.0421; LnL=−12.45
Tupaia_belangeri_b
Tupaia_belangeri_c
Tupaia_glis_a
Tupaia_belangeri_g
Tupaia_longipes
Tupaia_chrysogaster
Urogale_everetti
Tupaia_splendidula_a
Tupaia_montana
Tupaia_picta
Tupaia_tana_a
Tupaia_minor_a
Tupaia_palawanensis
Tupaia_moellendorffi
Tupaia_nicobarica
Tupaia_javanica
Tupaia_dorsalis_a
Tupaia_gracilis
Anathana_ellioti
Dendrogale_murina_a
Dendrogale_melanura
Ptilocercus_lowii_a
AsiaIndia
AsiaIndia
India
India
40
30
20
10
0
Millions of years ago

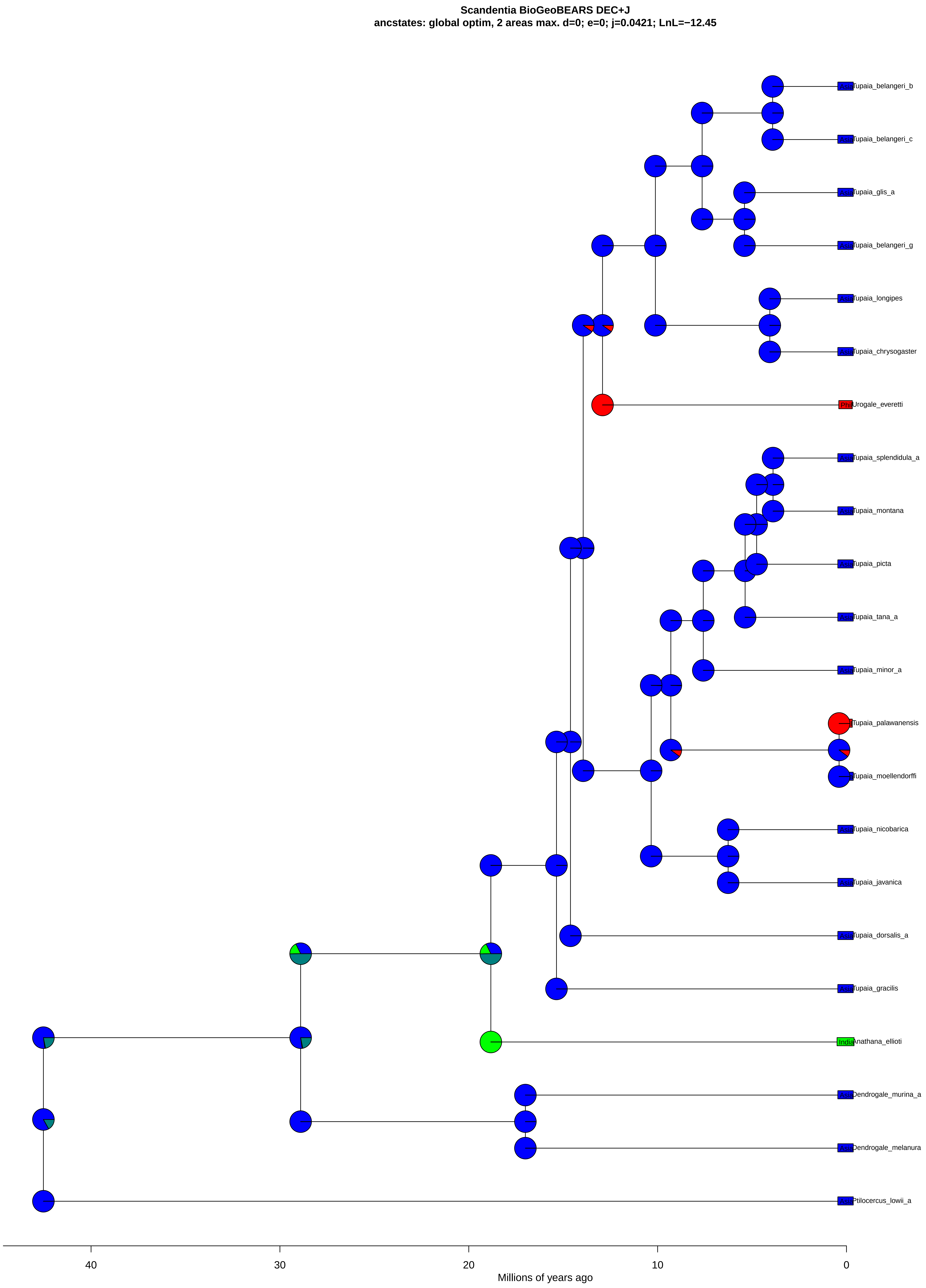
Scandentia BioGeoBEARS DEC+J
ancstates: global optim, 2 areas max. d=0; e=0; j=0.0421; LnL=−12.45
Tupaia_belangeri_b
Tupaia_belangeri_c
Tupaia_glis_a
Tupaia_belangeri_g
Tupaia_longipes
Tupaia_chrysogaster
Urogale_everetti
Tupaia_splendidula_a
Tupaia_montana
Tupaia_picta
Tupaia_tana_a
Tupaia_minor_a
Tupaia_palawanensis
Tupaia_moellendorffi
Tupaia_nicobarica
Tupaia_javanica
Tupaia_dorsalis_a
Tupaia_gracilis
India
Anathana_ellioti
Dendrogale_murina_a
Dendrogale_melanura
Ptilocercus_lowii_a
40
30
20
10
0
Millions of years ago

Cercopithecidae (Colobinae) BioGeoBEARS DEC+J
ancstates: global optim, 2 areas max. d=0; e=0; j=0.0138; LnL=−7.06

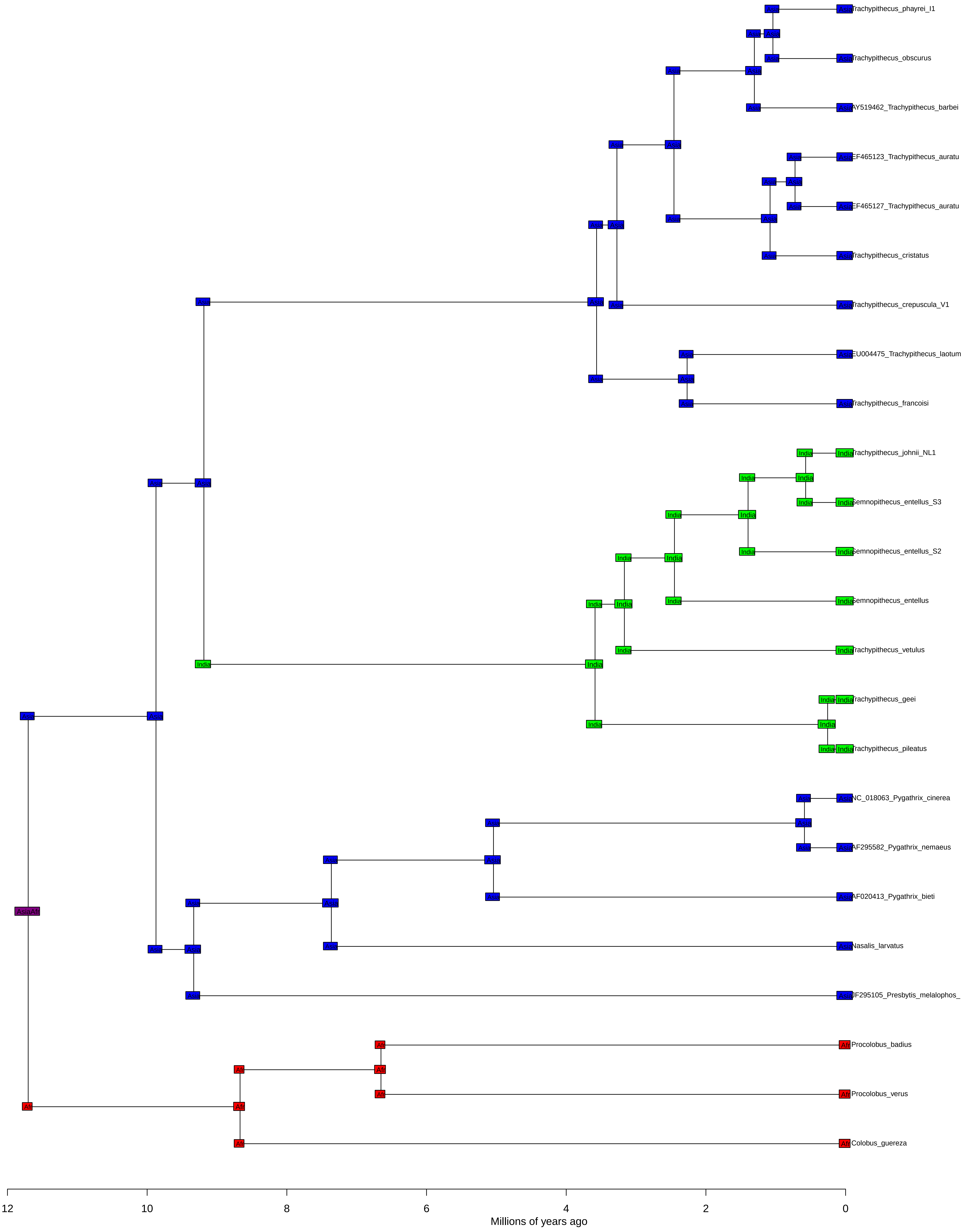

Cercopithecidae (Colobinae) BioGeoBEARS DEC+J
ancstates: global optim, 2 areas max. d=0; e=0; j=0.0138; LnL=−7.06

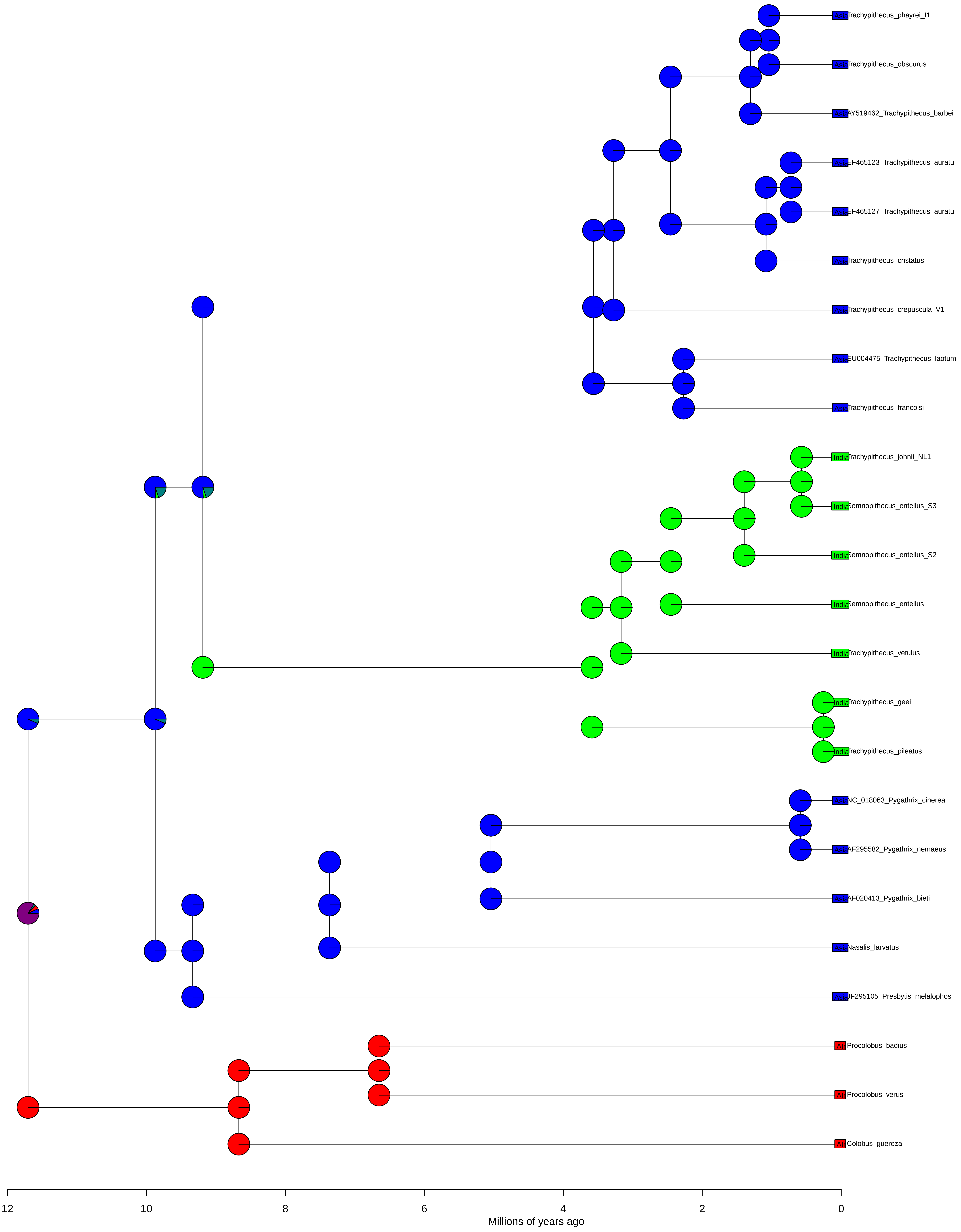

Supplementary Figure 2

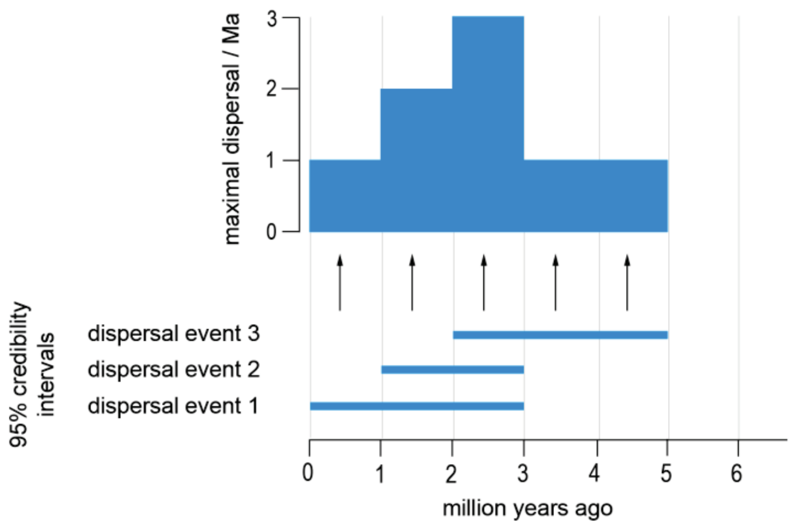

Suppl. Fig. 2 | Example for the translation of credibility intervals of dispersal time estimation into the maximal number of observed dispersal events per Ma. The credibility intervals for the time points of dispersal for three events overlap such that the MDE increases from one to two between 0 and 2 Ma, peaks between 2 and 3 Ma and drops down to one after 3 Ma.

Supplementary Figure 3

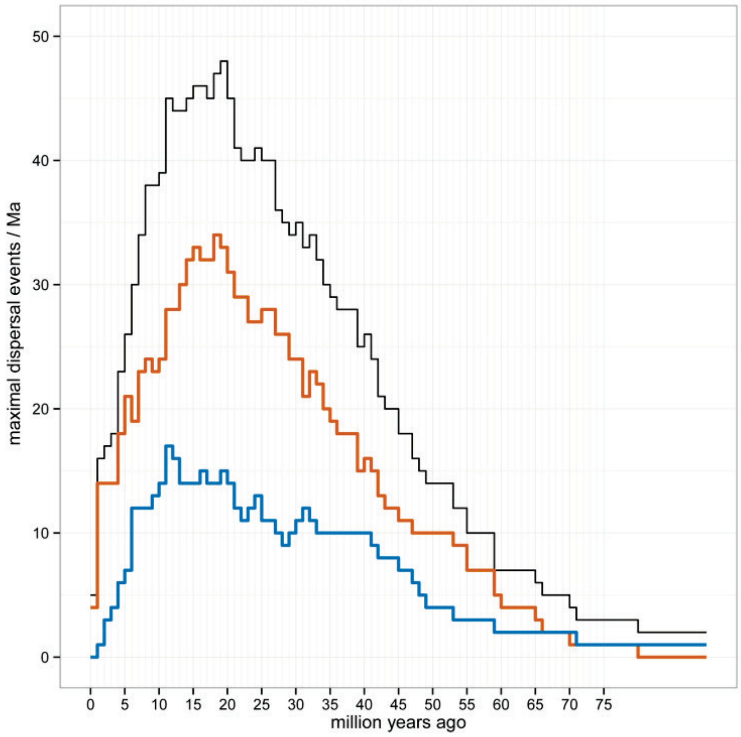

Suppl. Fig. 3 | Raw maximal dispersal events (MDE) before smoothing: Dispersal from mainland Asia to India (orange), from India to mainland Asia (blue), and in both directions (black).

Supplementary Figure 4

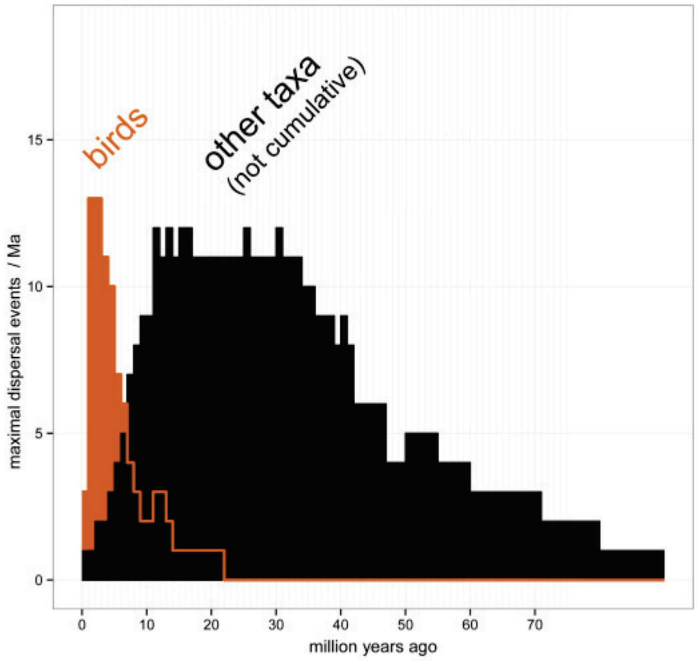

Suppl. Fig. 4 | Influence of taxon sampling on maximal dispersal rate. To account for a bias against more recent dispersal events due to increasing incomplete taxon sampling towards the present, we removed maximal dispersal events (MDE) with an age <7 Ma from the subsequent analyses based on the comparison of MDE between birds and other taxon groups (plants, arthropods, teleosts, amphibians, non-avian reptiles and mammals) with a less complete sampling (data not smoothed, dispersal rates for non-avian taxa not cumulative).

Supplementary Table 1

Taxon	95% HPD		Dispersal direction
	upper bound	lower bound	
Crypteroniaceae (Rosids, Myrtales)	5	1	A → I
Dipterocarpaceae (Rosids, Malvales)	79	36	A → I
Dipterocarpaceae (Rosids, Malvales)	59	26	A → I
Dipterocarpaceae (Rosids, Malvales)	54	18	A → I
Dipterocarpaceae (Rosids, Malvales)	28	13	A → I
Dipterocarpaceae (Rosids, Malvales)	108	50	I → A
Dipterocarpaceae (Rosids, Malvales)	70	31	I → A
Dipterocarpaceae (Rosids, Malvales)	44	18	I → A
Dipterocarpaceae (Rosids, Malvales)	29	7	I → A
Dipterocarpaceae (Rosids, Malvales)	27	5	I → A
Dipterocarpaceae (Rosids, Malvales)	24	10	I → A
Dipterocarpaceae (Rosids, Malvales)	20	6	I → A
Dipterocarpaceae (Rosids, Malvales)	16	4	I → A
Dipterocarpaceae (Rosids, Malvales)	14	2	I → A
Gentianaceae (Asterids, Gentianales)	17	8	A → I
Gentianaceae (Asterids, Gentianales)	13	6	I → A
Pachychilidae (Mollusca: Gastropoda)	65	12	A → I
Gecarcinucidae (Crustacea, Brachyura)	58	30	I → A
Gecarcinucidae (Crustacea, Brachyura)	47	24	I → A

Papilionidae (Insecta, Lepidoptera)	54	32	A → I
Papilionidae (Insecta, Lepidoptera)	30	11	A → I
Papilionidae (Insecta, Lepidoptera)	26	15	A → I
Papilionidae (Insecta, Lepidoptera)	26	15	A → I
Papilionidae (Insecta, Lepidoptera)	22	13	A → I
Papilionidae (Insecta, Lepidoptera)	22	11	A → I
Papilionidae (Insecta, Lepidoptera)	10	5	A → I
Papilionidae (Insecta, Lepidoptera)	20	12	I → A
Papilionidae (Insecta, Lepidoptera)	19	11	I → A
Papilionidae (Insecta, Lepidoptera)	16	9	I → A
Papilionidae (Insecta, Lepidoptera)	12	6	I → A
Apidae (Insecta, Hymenoptera)	27	11	A → I
Apidae (Insecta, Hymenoptera)	11	4	A → I
Apidae (Insecta, Hymenoptera)	14	4	A → I
Formicidae (Insecta, Hymenoptera, *Myrmica* spp.)	11	5	A → I
Formicidae (Insecta, Hymenoptera, *Tetraponera* spp.)	44	7	A → I
Aplocheiloidei (Teleostei, Cyprinodontiformes)	46	30	I → A
Channidae (Teleostei, Perciformes)	7	3	A → I
Channidae (Teleostei, Perciformes)	20	7	A → I
Channidae (Teleostei, Perciformes)	19	8	A → I
Channidae (Teleostei, Perciformes)	16	5	A → I
Osphronemidae (Teleostei: Perciformes)	35	23	A → I
Osphronemidae (Teleostei: Perciformes)	30	17	A → I
Osphronemidae (Teleostei: Perciformes)	33	20	A → I

Osphronemidae (Teleostei: Perciformes)	38	28	A → I
Osphronemidae (Teleostei: Perciformes)	38	22	A → I
Osphronemidae (Teleostei: Perciformes)	21	11	I → A
Cobitidae (Teleostei, Cypriniformes)	19	8	A → I
Cobitidae (Teleostei, Cypriniformes)	24	10	A → I
Cobitidae (Teleostei, Cypriniformes)	52	27	A → I
Heteropneustes fossilis (Teleostei, Siluriformes)	48	11	I → A
Dicroglossidae (Amphibia, Anura)	49	28	I → A
Dicroglossidae (Amphibia, Anura)	26	14	I → A
Dicroglossidae (Amphibia, Anura)	19	7	A → I
Dicroglossidae (Amphibia, Anura)	19	3	I → A
Dicroglossidae (Amphibia, Anura)	13	3	I → A
Microhylidae (Amphibia, Anura)	58	30	A → I
Rhacophoridae (Amphibia, Anura)	32	23	I → A
Rhacophoridae (Amphibia, Anura)	41	31	A → I
Rhacophoridae (Amphibia, Anura)	28	19	A → I
Rhacophoridae (Amphibia, Anura)	29	19	A → I
Rhacophoridae (Amphibia, Anura)	40	29	A → I
Bufonidae (Amphibia, Anura)	24	14	I → A
Bufonidae (Amphibia, Anura)	11	6	I → A
Bufonidae (Amphibia, Anura)	30	18	A → I
Bufonidae (Amphibia, Anura)	32	20	A → I
Bufonidae (Amphibia, Anura)	12	6	A → I
Bufonidae (Amphibia, Anura)	18	10	A → I

Bufonidae (Amphibia, Anura)	9	4	A → I
Geckonidae (Reptilia, Squamata, *Cyrtodactylus* spp.)	33	18	A → I
Geckonidae (Reptilia, Squamata, *Cyrtodactylus* spp.)	30	14	A → I
Geckonidae (Reptilia, Squamata, *Cyrtodactylus* spp.)	28	14	A → I
Geckonidae (Reptilia, Squamata, *Cyrtodactylus* spp.)	18	7	A → I
Geckonidae (Reptilia, Squamata, *Cyrtodactylus* spp.)	31	16	I → A
Scincidae (Reptilia: Squamata)	40	15	A → I
Scincidae (Reptilia: Squamata)	35	13	A → I
Scincidae (Reptilia: Squamata)	41	14	A → I
Scincidae (Reptilia: Squamata)	52	20	A → I
Scincidae (Reptilia: Squamata)	58	19	A → I
Scincidae (Reptilia: Squamata)	1	0	A → I
Scincidae (Reptilia: Squamata)	69	28	A → I
Agamidae 1 (Reptilia, Squamata)	46	32	A → I
Agamidae 1 (Reptilia, Squamata)	38	25	A → I
Agamidae 1 (Reptilia, Squamata)	27	17	I → A
Agamidae 1 (Reptilia, Squamata)	41	29	I → A
Agamidae 2 (Reptilia, Squamata)	10	20	A → I
Boidae (Reptilia, Squamata)	25	12	A → I
Viperidae (Reptilia, Squamata)	27	14	A → I
Viperidae (Reptilia, Squamata)	22	11	A → I
Crocodylidae (Reptilia, Crocodylia)	8	4	A → I
Crocodylidae (Reptilia, Crocodylia)	64	40	A → I
Timaliidae (Reptilia, Aves)	12	6	A → I

Timaliidae (Reptilia, Aves)	1	0	A → I
Timaliidae (Reptilia, Aves)	2	1	A → I
Timaliidae (Reptilia, Aves)	4	1	A → I
Timaliidae (Reptilia, Aves)	4	2	A → I
Timaliidae (Reptilia, Aves)	13	7	A → I
Paridae (Reptilia, Aves)	1	0	A → I
Paridae (Reptilia, Aves)	3	1	A → I
Paridae (Reptilia, Aves)	6	0	A → I
Aegithalidae (Reptilia, Aves)	1	0	A → I
Aegithalidae (Reptilia, Aves)	6	3	A → I
Aegithalidae (Reptilia, Aves)	7	4	A → I
Aegithalidae (Reptilia, Aves)	21	11	A → I
Fringillidae (Reptilia, Aves)	3	1	I → A
Fringillidae (Reptilia, Aves)	3	1	A → I
Fringillidae (Reptilia, Aves)	4	2	A → I
Certhiidae (Reptilia, Aves)	3	1	A → I
Certhiidae (Reptilia, Aves)	3	1	A → I
Certhiidae (Reptilia, Aves)	6	3	A → I
Certhiidae (Reptilia, Aves)	8	4	A → I
Psittacidae (Reptilia, Aves)	5	1	A → I
Psittacidae (Reptilia, Aves)	6	2	I → A
Psittacidae (Reptilia, Aves)	5	1	A → I
Nisaetus spp. (Reptilia, Aves, Accipitridae)	2	1	A → I
Bovidae (Mammalia, Ruminantia)	23	26	A → I

Cervidae (Mammalia, Ruminantia)	8	9	A → I
Bovidae (Mammalia, Ruminantia)	8	10	A → I
Bovidae (Mammalia, Ruminantia)	0	2	A → I
Herpestidae (Mammalia, Carnivora)	12	4	I → A
Herpestidae (Mammalia, Carnivora)	9	2	A → I
Herpestidae (Mammalia, Carnivora)	15	7	A → I
Herpestidae (Mammalia, Carnivora)	14	7	A → I
Sciuridae (Mammalia, Rodentia)	26	15	A → I
Sciuridae (Mammalia, Rodentia)	19	12	A → I
Sciuridae (Mammalia, Rodentia)	18	5	A → I
Tupaiidae (Mammalia, Scandentia)	34	25	A → I
Colobinae (Mammalia, Primates)	13	6	A → I

Suppl. Table 1 | Inferred dispersal events between mainland Asia and the Indian subcontinent. Given are the respective taxon, upper and lower bounds of the 95% highest posterior density intervals (HPD) for the age of range shifts/dispersal events in million years before present (Ma) as used for the calculation of the maximal number of dispersal events (MDE) per Ma between the Indian subcontinent (I) and mainland Asia (A).

Supplementary Note 1

Crypteroniaceae (Rosids, Myrtales): We re-analysed the data set of Rutschmann *et al.*[1], reduced to 44 taxa to estimate the divergence time between *Axinandra zeylanica* and SE-Asian *A. coriacea*. The data set included the *rbcL*, *ndhF*, *rpl16*-intron, *18S*, and *26S rRNA* genes (5,421 bp total alignment length). We initially applied the best calibration scheme as suggested by the authors of the original study[1]. However, we had severe difficulties in implementing all suggested calibration points as this dropped the initial likelihood to 'infinity'. Therefore, we could reduce the data set, omitting the families Myrtaceae *s. lat.*, Vochysiaceae, and Onagraceae. We kept a log-normal calibration density for the MRCA of *Rhexia virginica* and *Melastoma beccarianum* (minimum age = 23 Ma; 5–95% interquantile range = 23.9–46.2 Ma), and the MRCA of *Pternandra echinata* and *M. beccarianum* (minimum age = 53 Ma; 5–95% interquantile range = 53.9–76.2 Ma). Resulting mean rates were 0.1% per Ma (95% CI = 0.08–0.13% per Ma) for *ndhF*; 0.04% per Ma (95% CI = 0.03–0.05% per Ma) for *rbcL*; 0.1% per Ma (95% CI = 0.07–0.12% per Ma) for *rpl16*; 0.02% per Ma (95% CI = 0.01–0.02% per Ma) for *18S rRNA*; and 0.05% per Ma (95% CI = 0.04–0.06% per Ma) for *26S rRNA*. We defined as ranges India, mainland Asia, Africa and the New World.

Dipterocarpaceae (Rosids, Malvales): The phylogeny of the dipterocarp subfamily Dipterocarpoideae published by Gamage *et al.*[2] is based on the *trnL*-intron, t*rnL-trnF* spacer region and the *matK* gene (3,926 bp total alignment length). To calibrate the phylogeny, we applied the approach described by Gunasekara[3] and constrained the MRCA of the genus *Hopea* to a minimum of 12 Ma (log-normal calibration density, 5–95% interquantile range = 13.4–50.3),

and that of the genus *Vatica* to 3.6 Ma (lognormal calibration density, 5–95% interquantile range = 5.0–41.9 Ma) based on the fossil record. Additionally, we included (as 'empty sequences') dated fossils that are closely related to the genera *Dipterocarpus* (estimated age: 65 Ma[4]), *Dryobalanops* (estimated age: 20 Ma[5]) and *Anisoptera* (estimated age: 20 Ma[5]). We applied a broad exponential distribution as prior for the uncorrelated relaxed clock rate, with a mean of 0.2% per Ma (5–95% interquantile range = 0.01–0.60% per Ma). The resulting mean substitution rates were 0.03 % per Ma (95% CI = 0.02–0.05% per Ma) for *matK*; 0.1% per Ma (95% CI = 0.05–0.15% per Ma) for the *trnL*-intron; and 0.06 % per Ma (95% CI = 0.04–0.08% per Ma) for the *trnL-trnF* spacer region. We defined as ranges India, mainland Asia, Africa and the Seychelles. The subfamily Dipterocarpoideae is an example *par excellence* of a taxonomic group which—based on the biogeographical distribution of extant taxa and the fossil record—was formerly widespread in Africa, on the Indian Plate and associated continental fragments (such as the Seychelles). It subsequently diversified explosively in SE-Asia, but later became extinct in Africa and severely retracted its range in India. African fossils, including the genus *Dipterocarpus*[4], and pre-collision (i.e., Early Eocene) Indian macrofossils and geochemical biomarkers have been reported by Dutta *et al.*[5]. To reflect this prior knowledge, we constrained the range of the MRCA of Dipterocarpaceae to Africa for our biogeographical estimation and adjusted the dispersal multiplier (dm) for dispersal into Africa (dm = 0.0001; thus setting Africa as origin), from mainland Asia to the Seychelles (dm = 0.01), and from Africa and the Seychelles to mainland Asia (dm = 0.01), while we kept dm = 1.00 for all other dispersal directions.

Gentianaceae (Asterids, Gentianales): Yuan *et al.*[6] based their study on the genus *Exacum* on the nuclear encoded ribosomal *ITS-1* and *ITS-2* regions, the *5.8S rRNA*

gene, and the chloroplast *trnL*(UAA) intron sequence. Four independent fossil-based calibration points (minimum ages) were introduced in a phylogeny of the family Gentianaceae; resulting time estimates for the divergence between the outgroup (*Gentianothamnus–Tachiadenus* clade) and the ingroup (*Exacum–Ornichia* clade) were then used to calibrate the *Exacum* tree: tMRCA of Gentianales (60 Ma), tMRCA of *Lisianthius* (40 Ma), tMRCA of the subtribe Swertiinae (15 Ma), and tMRCA of *Gentiana* (5 Ma). We re-analysed the data set using an *ITS* rate of 0.452% per Ma (SD = 0.1)[7,8], and an exponential prior distribution for *5.8S rRNA* and *trnL* with a mean of 1% per Ma (5–95% interquantile range = 0.05–3.00% per Ma). We applied a normally distributed calibration density for the tMRCA of *Exacum* (mean = 22 Ma, SD = 8.3 Ma, 5–95% interquantile range = 8.3–35.7 Ma) following the study published by Yuan *et al.*[6]. The resulting mean rates for the unconstrained gene partitions were 0.05% per Ma (95% CI = 0.02–0.08% per Ma) for *5.8S rRNA* and 0.07% per Ma (95% CI = 0.04–0.10% per Ma) for *trnL*. We defined as ranges India, mainland Asia, the Himalayas, Africa, Madagascar and the island of Socotra.

Pachychilidae (Mollusca, Caenogastropoda): Unfortunately, fossil Pachychilidae have not been described that could be used for calibration, nor are reliable external rates for freshwater snails available. Therefore, we applied a broad external rate based on the data set presented by Köhler and Glaubrecht[9] (*16S rRNA*, and the *CO1* gene; 1,511 bp total alignment length) for dating the split between the genera *Brotia* and *Paracrostoma*. After an initial series of test runs, we used a fixed strict rate with a normal prior of 1% per Ma (SD = 1.25), truncated at 5% per Ma for both partitions (5–95% interquantile range = 0.0–20.7% per Ma). This broadly covers mitochondrial substitution rates of marine gastropods that have been calibrated using the closure of the Isthmus of Panama

as a geological calibration point[10,11]. We defined as ranges India, mainland Asia, Australia, Africa and the Americas.

Gecarcinucidae (Crustacea, Brachyura): Klaus *et al.*[12,13] based their phylogeny of Asian freshwater crabs on the nuclear-encoded histone *H3* (318 bp) gene and the mitochondrial *16S rRNA* gene (558 bp; 876 bp total alignment length. The phylogeny was calibrated using three fossil calibration points that were translated into a gamma-distributed calibration density: fossil *Potamonautes niloticus* (node *P. niloticus–Platythelphusa armata*: 6 Ma), fossil *Potamon quenstedti* (node *P. fluviatile–P. persicum*: 16.5 Ma), and fossil *Sartoriana* sp. (node *S. spinigera–S. blanfordi*: 2.5 Ma). According to a recent phylogeny of the family Potamonautidae[14], we corrected the first calibration point to comprise the MRCA of *Potamonautes niloticus* and *P. stanleyensis*. The analysis was run for 80 M iterations, and the first 30 M were discarded as burn-in. We applied a uniform distribution as prior for the substitution rates (0.1–10.0% per Ma for *16S rRNA* and 0.01–1.00% per Ma for histone *H3*). The resulting mean rates were 0.28% per Ma (95% CI = 0.18–0.37% per Ma) for *H3* and 0.41% per Ma (95% CI = 0.28–0.53% per Ma) for the *16S rRNA* gene. Especially the *16S rRNA* rate differs from previous studies (16S rRNA 0.64–1.42% per Ma; mean 1.02%; histone *H3* 0.12–0.26% per Ma, mean 0.19%)[12]. We used the same area coding as given in Klaus *et al.*[12] (India, East-/Southest Asia, Philippines and Wallacea).

Papilionidae (Insecta, Lepidoptera): Condamine *et al.*[15] based their phylogeny of papilionid butterflies on ~2.3 kb of mitochondrial *CO1* and *CO2* genes and ~1.0 kb of the nuclear *EF-1a* gene. We followed their calibration scheme and translated the following age estimates into log-normally distributed calibration densities: the MRCA of the family Papilionidae was set to a minimum age of 48 Ma (fossil *Praepapilio*; mean = 2.945 Ma, SD = 1.0, offset = 15 Ma, 5–95%

interquantile range = 51.7–146.5 Ma); 30 Ma was assumed as the minimum age for the subfamily Parnassiinae (mean = 3.0708 Ma, SD = 1.0, offset = 30 Ma, 5–95% interquantile range = 34.2–141.7 Ma); and the MRCA of the tribe Luehdorfiini was set to a minimum age of 15 Ma (mean = 3.164 Ma, SD = 1.0, offset = 15 Ma, 5–95% interquantile range = 19.6–137.6 Ma). The MRCA of family Pieridae was confined to a minimum age of 34 Ma (fossil *Stolopsyche libytheoides*), as was the MRCA of the family Nymphalidae (both mean = 3.044 Ma, SD = 1.0, offset = 34 Ma, 5–95% interquantile range = 38.1–142.7 Ma). We applied a broad exponential prior for the substitution rates of the partitions (*CO1* and *CO2*: mean = 3.0% per Ma, 5–95% interquantile range = 0.15–9.00% per Ma; *EF-1a*: mean = 1.0% per Ma, 5–95% interquantile range = 0.05–3.00% per Ma). We did not constrain the root age of the phylogeny as done in the original study (183 Ma, origin of angiosperms), as this resulted in an 'infinite negative' initial likelihood and abortion of the analysis. 8 M iterations were discarded as burn-in. The resulting mean rates were 0.71% per Ma (95% CI = 0.55–0.86% per Ma) for the combined *CO1*/*CO2* partition and 0.15% per Ma (95% CI = 0.12–0.18% per Ma) for *EF-1a*. The area coding was the following: India, SE Asia, Australia, the Palearctic, the Americas, Africa, and Madagascar

Apidae (Insecta, Hymenoptera): To retrieve credibility intervals we re-analysed a subset of data from the study by Rehan *et al.*[16] on the phylogeny of carpenter bees (subfamily Xylocopinae, tribe Ceratinini; 74 taxa) using the mitochondrially-encoded *CO1* and *Cytb*, and the nuclear *EF-1a* (2,807 bp total alignment length) that includes three dispersal events from mainland Asia to the Indian subcontinent (at the divergence nodes of *Ceratina propinqua* and *C. binghami*, and at the MRCA of *C. japonica*, *C. okinawana*, *C. bowringi*, *C. hieroglyphica* and *C. moderata*). We used the three calibration points as suggested by the authors with a

log-normal calibration density: a minimum divergence between the tribes Ceratinini and Allodapini of 45 Ma (5–95% interquantile range = 45.2–50.2 Ma); a minimum divergence between *Apis mellifera* from the genus *Liotrigona* of 65 Ma (5–95% interquantile range = 65.3–73.5 Ma); and 90 Ma (5–95% interquantile range = 91.4–128.3 Ma) for the MRCA of the Xylocopinae and Apinae. The resulting mean rates were 3.14% per Ma (95% CI = 1.32–7.07% per Ma) for *Cytb*, 0.28% per Ma (95% CI = 0.21–0.34% per Ma) for *EF-1a*, and 2.08% per Ma (95% CI = 1.66–2.54% per Ma) for *CO1*. We defined as ranges India, mainland Asia, Australia, Africa and the Americas.

Formicidae (Insecta, Hymenoptera): We combined partial sequence information of the *28S rRNA* and *LWRh* genes (1,388 bp total) obtained from the studies of Ward and Downie[17] on the subfamily Pseudomyrmecinae and Jansen *et al.*[18] on the genus *Myrmica*. Thereby, we could employ the calibration points provided by Jansen *et al.*[18], i.e., the split between the subfamilies Formicinae and Myrmicinae, calibrated with fossil *Kyromyrma* (92 Ma, 5–95% interquantile range = 92.2–124.0 Ma) and the MRCA of the genus *Myrmica*, calibrated based on baltic amber fossils (44.1 Ma; 5–95% interquantile range = 41.3–73.2 Ma). We inferred dispersal times to the Indian subcontinent for *Myrmica indica* and *T. rufonigra*, with separate biogeographical analyses for the subfamily Pseudomyrmecinae and the genus *Myrmica*. The resulting mean rates were 0.08% per Ma (95% CI = 0.07–0.10% per Ma) for *28S rRNA*; and 0.34% per Ma (95% CI = 0.26–0.42% per Ma) for *LWRh*. For the biogeographical analysis we split the resulting phylogeny and used different area coding for *Pseudomyrmex–Tetraponera* (India, mainland Asia, Africa and the Americas) and for *Myrmica* (India, East/Southeast Asia, western Eurasia/Europe and the Americas.

Aplocheiloidei (Teleostei, Cyprinodontiformes): We used the mitochondrial data set of Murphy and Collier[19] to calibrate the split between SE-Asian *Aplocheilus panchax* and Indian *A. lineatus*, consisting of partial sequences of *12S rRNA* (333 bp), *16S rRNA* (520 bp), and *Cytb* (360 bp) genes. We applied a normally distributed divergence density for the calibrations of the following splits: the split between American and African taxa (tectonic break up of western Gondwana, 5–95% interquantile range = 81.7–88.3 Ma); the split between Asian and Madagascan species (tectonic separation of Madagascar and India, 5–95% interquantile range = 85.4–88.6 Ma); and the age of the separation of Afrotropical/Neotropical and Asian/Madagascan taxa (break up of Western and Eastern Gondwana, 5–95% interquantile range = 166.7–173.3 Ma). The resulting mean rates were 0.30% per Ma (95% CI = 0.23–0.38% per Ma) for *16S rRNA*; 0.21% per Ma (95% CI = 0.17–0.26% per Ma) for *12S rRNA*; and 1.45% per Ma (95% CI = 1.07–1.89% per Ma) for *Cytb*. For the biogeographical inference, we used mainland Asia, India, Africa, Madagascar, the Seychelles and the Americas as area coding.

Channidae (Teleostei, Perciformes): We re-calculated the phylogeny published by Adamson *et al.*[20] that was based on the mitochondrially encoded *Cytb* (809 bp) and nuclear *RAG-1* (1,484 bp) genes. We followed their calibration scheme and constrained the MRCA of the genera *Channa* and *Parachanna* with the first occurrence date of *Channa* (5–95% interquantile range = 40.9–63.2 Ma); and the divergence of the family Channidae from other members of the order Perciformes (5–95% interquantile range = 48.6–103.3 Ma). We applied broad normally distributed rate priors for *Cytb* (2.0% per Ma, SD = 1.0, 5–95% interquantile range = 0.54–3.66% per Ma) and *RAG-1* (0.1% per Ma, SD = 0.1, 5–95% interquantile range = 0.02–0.27% per Ma) Resulting mean rates were 0.51% per

Ma (95% CI = 0.43–0.61% per Ma) for *Cytb* and 0.15% per Ma (95% CI = 0.12–0.20% per Ma) for the *RAG-1* gene. For the biogeographical inference, we used mainland Asia and India as area coding. The distribution range at the root node was constrained to Asia[20].

Heteropneustidae (Teleostei: Siluriformes): We re-analysed data from the phylogenetic study by Ratmuangkhwang *et al.*[21] on heteropneustid catfishes which are currently described as one species (*Heteropneustes fossilis*), based on *RAG-1* sequence information of 1,494 bp length. We followed the calibration scheme of the original study by incorporating six calibration points as normally distributed calibration densities within the outgroup taxa. These were derived as secondary calibration points from the study of Nakatani *et al.*[22] (phylogeny of the teleost group Otophysi; the calibration was based on 23 fossil and three biogeographical constraints), such that the 5–95% interquantile ranges correspond to the node age credibility intervals of the latter study. The resulting mean substitution rate was 0.96% per Ma (95% CI = 0.59–1.34% per Ma). The mutation rate under the calibration scheme of Ratmuangkhwang *et al.*[21] is thus extremely fast compared to the mutation rates of *RAG-1* inferred for the Channidae (see above) and Osphronemidae (see below), and might point to an underestimation of divergence dates. However, the resulting credibility intervals for the estimates of node ages were large (spanning >30 Ma) and thus, most likely still captured the actual divergence times. For the biogeographical inference, we used mainland Asia and India as area coding.

Osphronemidae (Teleostei: Perciformes): We re-analysed the data from the study of Rüber *et al.*[23] on the phylogeny of fishes in the family Osphronemidae. We used the complete data set comprising 60 taxa (*RAG-1* gene, complete *Cytb*, partial *12S rRNA*, *Val-tRNA* and partial *16S rRNA* genes; 4,258 bp total alignment

length). We followed the authors' calibration scheme by calibrating with fossil *Osphronemus* from Sumatra of Late Eocene/Early Oligocene age, applying the suggested crown group calibration. We translated this information into a log-normally distributed calibration density with an offset at 28 Ma (5–95% interquantile range = 29–54 Ma). Resulting rates were 0.34% per Ma for *RAG-1* (95% CI = 0.26–0.40% per Ma), 3.01% per Ma for *Cytb* (95% CI = 2.31–3.65% per Ma), and 0.47% per Ma (95% CI = 0.36–0.57% per Ma) for the combined non-protein coding mitochondrial tRNA and rRNA genes. For the biogeographical inference, we used mainland Asia, India and Africa as area coding.

Cobitidae (Teleostei, Cypriniformes): We re-analysed the data set on which the phylogenetic study by Šlechtová *et al.*[24] builds (mitochondrial *Cytb* and nuclear *RAG-1* sequences; 2,016 bp total alignment length) using an external rate for *Cytb* (normally distributed, with a mean rate of 0.68% per Ma; 5–95% interquantile range = 0.52–0.84% per Ma). A calibration for the genus *Cobitis* was available using the opening of the Strait of Gibraltar, as cobitids are primary freshwater fish and thus, incapable of dispersing through marine habitats[25]. We assumed three dispersal events from Southeast Asia to the Indian subcontinent (Bangladesh and N-Bengal) at the divergence of *Pangio pangia* and *P. doriae*; *Lepidocephalichtys guntea* and *L. hasselti*; and *Neoeucirrichthys maydelli* and *Somileptus gongota*. The resulting mean mutation rate of the *RAG-1* gene was 0.17% per Ma (95% CI = 0.12–0.23% per Ma). For the biogeographical inference, we used mainland Asia and India as area coding.

Dicroglossidae (Amphibia, Anura): In case of the anuran family Dicroglossidae we re-analysed the respective subset of data from the amphibian phylogeny published by Pyron and Wiens[26], including the genera *Indirana* (outgroup),

Occidozyga, *Nannophrys*, *Hoplobatrachus*, *Euphlyctis*, *Sphaerotheca*, *Fejervarya*, *Paa*, and *Limnonectes*), while we included three additional specimens of the genus *Fejervarya*[27]. The data consist of seven partitions, three mitochondrial (*12S* and *16S rRNA* genes, *Cytb*) and four nuclear genes (*CXCR4*, *NCX1*, *RHOD*, *TYR*). We assigned a GTR+G model of sequence evolution to all partitions except for the *12S rRNA* and *CXCR4* partitions, in which case a HKY+G model was applied. We ran the analysis for 50 M generations, sampling every 2,000th generation, discarding 10% of the samples as burn-in. We constrained the tMRCA of dicroglossids with a normally distributed calibration density with a mean of 63 Ma (SD = 9.7 Ma; 5–95% interquantile range = 47–79 Ma). This secondary calibration point is based on the study of Van Bocxlaer *et al.*[28], as the different calibration schemes employed by the authors cover a range of 47–79 Ma for this split. For the biogeographical inference, we used mainland Asia, Africa and India as area coding. Although not unequivocal in our analysis, we assumed an Indian origin of the family Dicroglossidae, and thus initial dispersal from the Indian subcontinent to East-/SE-Asia at the deepest split within the subfamily Dicroglossinae[28]. The resulting mean rates were 0.74% per Ma (95% CI = 0.56–0.95% per Ma) for *12 rRNA*; 0.87% per Ma (95% CI = 0.64–1.10% per Ma) for *16S rRNA*; 3.70% per Ma (95% CI = 2.27–5.48% per Ma) for *Cytb*; 0.07% per Ma (95% CI = 0.04–0.12% per Ma) for *CXCR4*; 0.06% per Ma (95% CI = 0.04–0.08% per Ma) for *NCX1*; 0.08% per Ma (95% CI = 0.05–0.12% per Ma) for *RHOD*; and 0.12% per Ma (95% CI = 0.09–0.16% per Ma) for *TYR*.

Microhylidae (Amphibia, Anura): For the family Microhylidae we re-analysed a part of the data set published by de Sá *et al.*[29], in which three nuclear (*TYR*, *BDNF*, and the *28S rRNA* gene) and one mitochondrial marker (*16S rRNA* gene) were employed. We ran the analysis for 100 M iterations, sampling every 10,000th

iteration, discarding 10% as burn-in. We applied a HKY+G model of sequence evolution for all partitions. According to the original study[29] we constrained (using a normally distributed calibration density) the tMRCA of the subfamily Otophryninae to 60.4 Ma (SD = 5); the tMRCA of subfamily Gastrophryninae to 79.1 Ma (SD = 5); and the tMRCA of the genus *Gastrophryne* to 1.7 Ma (SD = 0.5). The resulting mean rates were 0.06% per Ma (95% CI = 0.05–0.07% per Ma) for *BDNF*; 0.19% per Ma (95% CI = 0.15–0.25% per Ma) for *TYR*; 0.017% per Ma (95% CI = 0.01–0.02% per Ma) for *28S rRNA*; and 0.89% per Ma (95% CI = 0.69–1.11% per Ma) for *16S rRNA*. For the biogeographical inference, we used mainland Asia and India as area coding.

Rhacophoridae (Amphibia, Anura): The study on rhacophorid tree frogs[30] was based on five nuclear gene fragments (*BDNF*, 614 bp; *POMC*, 601 bp; *RAG-1*, 1,164 bp; *RHOD*, 315 bp; *TYR*, 531 bp) and 2,041 bp of mitochondrial DNA covering the *12S* and *16S rRNA* genes as well as the complete *tRNA Val* (5,266 bp total alignment length). The authors calibrated the age of the most recent common ancestor of the Rhacophoridae with fossil *Indorana prasadi* from Early Eocene sediments of India, and constrained the split between *Boophis tephraeomystax* from the Comoro island of Mayotte and its sister species *Boophis doulioti* to a maximum age of 15 Ma. We did not re-analyse this data set but used the original chronogram to calculate the biogeographic estimates. For the biogeographical inference, we used mainland Asia, India, insular SE Asia and Africa as area coding.

Bufonidae (Amphibia, Anura): We re-analysed the data set of Van Bocxlaer *et al.*[31], which included nuclear (1,970 bp; *NCX1*, *CXCR4*) and mitochondrial genes (4,339 bp; *12S rRNA*, *tRNA Val*, *16S rRNA*, *tRNA Leu*, *ND1*, *tRNA Ile*, *tRNA Gln*, *tRNA Met*, *ND2*; 6,309 bp total alignment length), to infer the phylogeny and

divergence time estimates of toads, with the focus on the Asian subfamily Adenominae. We translated the following calibration points based on minimum ages of fossils into log-normally distributed calibration densities (SD = 1.0 in all cases): 18 Ma for the MRCA of the *Bufo viridis* group (mean = 1.7 Ma, offset = 18.0 Ma, 5–95% interquantile range = 19.1–46.7 Ma), 11 Ma for the origin of the genus *Rhinellamarina* (mean = 1.88 Ma, offset = 11.0 Ma, 5–95% interquantile range = 12.3–44.8 Ma), 9.6 Ma for the MRCA of the *Bufo bufo* group (mean = 1.905 Ma, offset = 9.6 Ma, 5–95% interquantile range = 10.9–44.4 Ma), and a minimum age of 15 Ma for the MRCA of the subgenus *Eleutherodactylus* (mean = 2.17 Ma, offset = 15.0 Ma, 5–95% interquantile range = 16.7–60.3 Ma). Divergence time analysis was run as described under "Phylogenetic methods and divergence time estimations" with the exception that we discarded 2,500 samples as burn-in. The resulting mean rates were 0.09% per Ma (95% CI = 0.07–0.11% per Ma) for the nuclear and 0.88% per Ma (95% CI = 0.65–1.10% per Ma) for the mitochondrial partition. For the biogeographical inference, we used East/SE Asia, India, Africa, and western Eurasia as area coding.

Crocodylidae (Reptilia, Crocodylia): Oaks[32] presents a phylogeny of the family Crocodylidae based on two partitions: 7,282 bp of mitochondrial *Cytb*, *tRNA Glu*, *tRNA Thr*, *ND2*, *tRNA Met*, *tRNA Trp*, *ND3*, *tRNA Gly*, *tRNA Arg*), the D-loop of the control region and *tRNAPhe*, and nuclear encoded *c-mos*, *EPIC*, *ACTC* Exon 4–5, *aTROP* Exon 5–6, *ACTB* Exon 3–4, *AChR* Exon 7–8, *GAPDH* Exon 11–12, *LDH-B* Exon 6–7, *LDH-A* Exon 7–8, and *RHO* Exon 2–3. This phylogeny was calibrated with the tMRCA of the subfamilies Alligatorinae and Caimaninae (5–95% interquantile range of a lognormal calibration density = 71–64 Ma; upper bound of the tMRCA of the order Crocodylia = 90 Ma). The *BEAST[33] minimum credibility tree was kindly provided by the first author of this study and used for

our biogeographical inference. For the biogeographical inference, we used mainland Asia, India, Africa and the Americas as area coding. We assumed dispersal from SE-Asia to the Indian subcontinent at the divergence of *Crocodylus siamensis* and *C. palustris*, and – more arbitrary in light of the fossil record[34] – at the MRCA of the genera *Tomistoma* and *Crocodylus*.

Geckonidae (Reptilia, Squamata): Based on the study of Wood *et al.*[35], including additional outgroup taxa from Bansal and Karanth[36], we calculated a phylogeny of the gekkonid genus *Cyrtodactylus* based on the partial nuclear *RAG-1* and *PDC* genes (1,454 bp total alignment length). Substitution schemes were not partitioned between both genes. We calibrated the divergence between *Sphaerodactylus roosevelti* and *S. torrei* based on fossil *Sphaerodactylus* from Hispaniola, dated 15–20 Ma (exponential calibration density, mean = 3.0 Ma, offset = 15 Ma, 5–95% interquantile range = 15–24 Ma), the divergence between the genera *Oedura* and *Woodworthia* with fossil "*Hoplodactylus* sp." from New Zealand, dated to 16–19 Ma (exponential calibration density, mean = 17 Ma, offset = 16 Ma, 5–95% interquantile range = 17–67 Ma). Fossil *Primaderma nessovi* served to constrain the MRCA of the split between the families Helodermatidae and Anguidae (exponential calibration density, mean = 3.0, offset = 99 Ma, 5–95% interquantile range = 99–108 Ma), and the split between *Teratoscincus scincus* and *T. roborowskii* was constrained biogeographically to 10 Ma (mean = 10 Ma, SD = 0.5, 5–95% interquantile range = 9–11 Ma). The resulting mean substitution rates were 0.08% per Ma for both partitions (95% CI = 0.06–0.09% per Ma for *RAG-1* and 0.06–0.1% per Ma for *PDC*). For the biogeographical inference, we used mainland Asia, India and Australia as area coding.

Scincidae (Reptilia: Squamata): We re-analysed the data used for the phylogenetic analyses published by Datta-Roy *et al.*[37] based on partial

mitochondrial *12S* and *16S rRNA* genes and the nuclear *c-mos* gene (1,641 bp total alignment length). We added the species *Coprucia zebrata*, *Tiliqua adelaidensis* and *Egernia whitii* from the study of Honda *et al*[38] and calibrated the split between the latter two based on fossil *Proegernia palankarinnensis* from the Late Oligocene[39]. We used a lognormal calibration density with 25 Ma as a hard younger bound, resulting in a 5–95% interquantile range of 25.52–39.08 Ma for that split. The resulting rates were 0.33% per Ma (95% CI 0.19–0.47% per Ma) for *16S rRNA*, 0.30% per Ma (95% CI 0.18–0.43% per Ma) for *12S rRNA*, and 0.04% per Ma (95% CI 0.02–0.06% per Ma) for *c-mos*. For the biogeographical inference, we used mainland Asia, India, Africa, Madagascar, the Seychelles, teh Cape Verde silands and the Americas as area coding.

Agamidae 1 (Reptilia, Squamata): We re-analysed the data set of Macey *et al.*[40], based on the mitochondrial *ND1*, *tRNA Gln*, *tRNA Ile*, *tRNA Met*, *ND2*, *tRNA Trp*, *tRNA Ala*, *tRNA Asn*, *tRNA Cys*, *tRNA Tyr*, and *CO1* genes (1,551 bp total alignment length). We used three fossil calibration points with a lognormal distributed calibration density[41] and calibrated the MRCA of crown Acrodonta (families Chamaeleonidae and Agamidae; 5–95% interquantile range = 46.4–134.0 Ma), the MRCA of crown Agamidae (5–95% interquantile range = 32.3–124.4 Ma), and the MRCA of *Istiurus lesuseurii* and *Pogona vitticeps* (5–95% interquantile range = 20.4–56.7 Ma). The resulting rates were 2.20% per Ma (95% CI 1.20–3.20% per Ma) for *CX1*, 1.90% per Ma (95% CI 1.10–2.51 per Ma) for *ND1*, 1.32% per Ma (95% CI 1.08–1.56 per Ma) for *ND2*, 0.95% per Ma (95% CI 0.68–1.23 per Ma) for *tRNA Ala*, 0.87% per Ma (95% CI 0.49–1.24 per Ma) for *tRNA Asn*, 1.50% per Ma (95% CI 1.09–1.95 per Ma) for *tRNA Cys*, 0.61% per Ma (95% CI 0.42–0.82 per Ma) for *tRNA Gln*, 0.66% per Ma (95% CI 0.37–0.97 per Ma) for *tRNA Ile*, 1.50% per Ma (95% CI 0.94–2.15 per Ma) for *tRNA Met*,

1.53% per Ma (95% CI 1.14–1.94 per Ma) for *tRNA Trp*, and 1.46% per Ma (95% CI 1.08–1.86 per Ma) for *tRNA Tyr*. For the biogeographical inference, we used mainland Asia, India, and Africa as area coding.

Agamidae 2 (Reptilia, Squamata): The aforementioned data set on agamids included only one Asian specimen of *Draco*; however, there are also Indian representatives this genus. Therefore, we re-analysed the data set of Honda *et al.*[42] based on mitochondrial *12S* and *16S rRNA* gene fragments (780 bp total alignment length). To estimate the divergence of Indian *D. dussumieri*, we calibrated the root of the tree (tMRCA [*Draco* + *Aphaniotis fusca*] with a secondary calibration point based on the previous analysis ('Agamidae 1'; normal calibration density with 5–95% interquantile range of 32–45 Ma). The resulting rates were 1.30% per Ma (95% CI = 0.91–1.74 per Ma) for *12S*; and 0.71% per Ma (95% CI = 0.48–0.96 per Ma) for the *16S rRNA* gene. For the biogeographical inference, we used mainland Asia and India as area coding.

Boidae (Reptilia, Squamata): We reanalysed the phylogeny of the family Boidae by Noonan and Chippindale[43] based on the nuclear *RAG-1*, *c-mos*, *NT3*, *ODC*, and *BDNF* genes and the mitochondrial *Cyt-b* gene (~4.3 kb total alignment length). We followed the suggestions for calibrating snake phylogenies provided by Sanders *et al*[44].: the MRCA of *Acrochordus javanicus* and the clade including *A. granulatus and A. arafurae* (5–95% interquantile range = 18.7–25.0 Ma), the split between the genus *Acrochordus* and the superfamily Colubroidea (5–95% interquantile range = 51.0–69.9 Ma), the MRCA of Boidae (5–95% interquantile range = 57.3–83.7 Ma), the split between the families Colubridae and Elapidae (5–95% interquantile range = 21.7–35.1 Ma), and the most basal split in extant snakes (uniform calibration density = 85.0–140.0 Ma). We assumed dispersal from SE Asia to India to have occurred between the MRCA of *Eryx colubrinus*

and the clade comprising *E. johni* and *E. conicus*, and the MRCA of *E. johni* and *E. conicus*, resulting in a time range of the combined 95% credibility intervals of 25–5 Ma. The resulting rates were 0.07% per Ma (95% CI = 0.05–0.09% per Ma) for *ODC*, 0.03% per Ma (95% CI = 0.02–0.04% per Ma) for *BDNF*, 0.05% per Ma (95% CI = 0.03–0.06% per Ma) for *RAG-1*, 0.05% per Ma (95% CI = 0.04–0.06% per Ma) for *c-mos*, and 0.07% per Ma (95% CI = 0.05–0.1% per Ma) for *NT3*. For the biogeographical inference, we used mainland Asia, India, Australia, Africa and the Americas as area coding.

Viperidae (Reptilia, Squamata): We re-analysed the mitochondrial data set used in the study of Wüster *et al.*[45] on the phylogeny of viperid snakes (based on *Cytb*, *ND4*, *12S* and *16S rRNA* genes; 2,962 bp total alignment length) that—following biogeographical inference—includes two dispersal events from SE-Asia to India, namely, at the split nodes between *Hypnale hypnale* and *Calloselasma rhodostoma*, and between *Trimeresurus borneensis* and *T. trigonocephalus*. We incorporated five calibrations points: the divergence of South American populations of the Neotropical pitviper genus *Porthidium* with the uplift of the Isthmus of Panama (mean = 3.5 Ma, SD = 0.51, 5–95% interquantile range = 2.7–4.4 Ma), minimum divergence of the Eurasian viper clade (excluding *Pseudocerastes* and *Eristicophis*) at 20 Ma (lognormal calibration density, mean = 1.0 Ma, SD = 1.0, offset = 20.0 Ma, 5–95% interquantile range = 20.5–34.1 Ma), a minimum age of 16 Ma for the divergence between the Asian clade of the genus *Naja* and its African sister clade (lognormal calibration density, mean = 1.0 Ma, SD = 1.0, offset = 16.0 Ma, 5–95% interquantile range = 16.5–30.1 Ma), the divergence between *Crotalus* and *Sistrurus* before 9 Ma (lognormal calibration density, mean = 0.0 Ma, SD = 1.0, offset = 9.0 Ma, 5–95% interquantile range = 9.2–14.2 Ma), and the tMRCA of the genus *Hemorrhois* (normal calibration

density, mean = 18.0 Ma, SD = 2.04, offset = 9.0 Ma, 5–95% interquantile range = 14.6–21.4 Ma). The resulting mean substitution rates were 1.64% per Ma (95% CI = 1.41–1.87% per Ma) for *ND4*, 2.12% per Ma (95% CI = 1.82–2.42% per Ma) for *Cytb*, 0.75% per Ma (95% CI = 0.60–0.92% per Ma) for *16S*, and 1.39% per Ma (95% CI = 0.95–1.89% per Ma) for *12S rRNA*. For the biogeographical inference, we used mainland Asia, India, Australia, Africa and the Americas as area coding.

Passeriform birds (Reptilia, Aves): Päckert *et al.*[46] presented a study on the biogeography of Himalayan and SE Asian songbirds (order Passeriformes) that included time-calibrated phylogenies (Bayesian approach in BEAST 1.4.8) and biogeographical reconstructions (parsimony approach) of several genera belonging to the families Phylloscopidae (*Cytb*, *12S rRNA* gene, *myoglobin* intron 2; 1,899 bp total alignment length), Timaliidae (*Cytb*; 861 bp; external rate 2.1% per Ma), Paridae (*Cytb*, *16S rRNA* gene, control region, and *fib7*; 2,527 bp total alignment length), Aegithalidae (*Cytb*, *16S rRNA* gene, *ND2*, *fib7*, *GAPDH11*, *ODC6*, and *TGFB2*; 3,995 bp total alignment length), Fringillidae (genus *Pyrrhula*; *Cytb*, *16S rRNA* gene, *fib7*, and *GAPDH11*; 2,357 bp total alignment length) and Certhiidae (*Cytb*, *16S rRNA* gene, *myo2*, and *GAPDH11*; 2,019 bp total alignment length) that we re-analysed for the present study. They calibrated these trees with various biogeographic events, i.e., the opening of the Bering Strait (uniform calibration density = 10.0–4.8 Ma and 14.0–4.8 Ma, respectively), the Messinian salinity Crisis (5.96 Ma), Pleistocene glaciations (2.4–0.18 Ma), the age of lava flows to calibrate splits between Africa and the Canary Islands (5.96–1.22 Ma), within the Canary Islands (1.77–0.00 Ma, 1.22–0.00 Ma), between Africa and the Azores (0.88–0.00 Ma), and within the Azores (0.2–0.0 Ma). However, we were not able to run the analyses under these calibration schemes, as

these priors resulted in extremely low initial model likelihoods. Therefore, we applied an external substitution rate for the *Cytb* partition of 1.35 % per Ma (SD = 0.4; 5–95% interquantile range = 0.7–2.3% per Ma). This broadly covers previously suggested avian *Cytb* substitution rates[47,48]. The resulting divergence times are considerably younger than in the original study (except for the Timaliidae), indicating that Päckert *et al.*[46] used pairwise divergence instead of substitution rates. We included those splits that separated west-Himalayan endemics (the authors' 'bioregion F04a') from their eastern relatives.

Psittacidae (Reptilia, Aves): We re-analysed the phylogeny by Groombridge *et al.*[49] of the genus *Psittacula* (*Cytb*, 800 bp), applying the above-mentioned external rate of 1.35 % per Ma (SD = 0.4; 5–95% interquantile range = 0.7–2.3% per Ma). We inferred a shift from SE-Asia to India at the divergence of *P. cyanocephala* and *P. longicauda*, and for the other dispersal direction at the divergence of *P. columboides*. For the biogeographical inference, we used mainland Asia, India, and Africa as area coding.

Accipitridae (Reptilia, Aves): We re-analysed the phylogeny of Asian hawk-eagles of the genus *Nisaetus* published by Gamauf *et al.*[50] that used mitochondrial *Cytb* and control region sequences (502 bp total alignment length). We applied an external rate of 1.35 % per Ma (SD = 0.4; 5–95% interquantile range = 0.7–2.3% per Ma) under an uncorrelated relaxed lognormal clock prior. For the biogeographical inference, we used mainland Asia and India as area coding. We inferred dispersal from Asia to India along the branch leading to the MRCA of the subspecies of *Nissaetus cirrhatus*.

Bovidae (Mammalia, Ruminantia): We re-analysed parts of the data set from the study by Hassanin *et al.*[51], who presented a phylogeny of the Cetartiodactyla

based on partial mitochondrial genome sequences (14,904 bp total alignment length). We followed their approach and calibrated (with normally distributed prior densities) the tMRCA of whales (Cetaceae) to 35 Ma (SD = 1), the tMRCA of the Cetaceae and *Hippopotamus* to 55 Ma (SD = 5), the tMRCA of Hippopotamus to 8 Ma (SD = 1), the divergence between the tribes Muntiacini and Cervini to 9 Ma (SD = 1), the tMRCA of the tribe Odocoileini to 5 Ma (SD = 1), and the tMRCA of the family Bovidae to 20 Ma (SD = 2). We removed 1,300 samples of the Bayesian analysis as burn-in. The resulting rates were 0.38% per Ma (95% CI = 0.35–0.42% per Ma) for the tRNAs, 0.35% per Ma (95% CI = 0.32–0.31% per Ma) for *12S rRNA*, 0.4% per Ma (95% CI = 0.37–0.44% per Ma) for *16S rRNA*, 0.80% per Ma (95% CI = 0.74–0.85% per Ma) for *ND1*, 0.96% per Ma (95% CI = 0.90–1.02% per Ma) for *ND2*, 0.75% per Ma (95% CI = 0.70–0.80% per Ma) for *CO1*, 0.69% per Ma (95% CI = 0.64–0.73% per Ma) for *CO2*, 0.75% per Ma (95% CI = 0.70–0.80% per Ma) for A8A6, 0.82% per Ma (95% CI = 0.76–0.88% per Ma) for *CO3*, 0.81% per Ma (95% CI = 0.75–0.89% per Ma) for *ND3*, 0.85% per Ma (95% CI = 0.79–0.90% per Ma) for *ND4*, 0.85% per Ma (95% CI = 0.79–0.90% per Ma) for *ND5*, 1.10% per Ma (95% CI = 1.00–1.20% per Ma) for *ND6*, and 0.90% per Ma (95% CI = 0.84–0.96% per Ma) for *CBP*. For the biogeographical inference, we used mainland Asia, India, Australia, Africa and the Americas as area coding.

Herpestidae (Mammalia, Carnivora): We re-analysed the data from the phylogenetic study by Patou *et al.*[52] on mongooses (family Herpestidae) based on the mitochondrial *Cytb* and *ND2* genes, and nuclear *FGBi7* sequences (2,770 bp total alignment length). We followed the authors' approach and calibrated the minimum age of the MRCA of the Herpestidae with fossil *Leptoplesictis* (uniform calibration density = 18–66 Ma), and of the MRCA of the genus *Galerella* based

on fossil evidence (7–66 Ma). The resulting mean substitution rates were 0.69% per Ma (95% CI = 0.50–0.87% per Ma) for *Cytb*, 0.56% per Ma (95% CI = 0.41–0.70% per Ma) for *ND2*, and 0.12% per Ma (95% CI = 0.08–0.16% per Ma) for *FGBi7*. For the biogeographical inference, we used SE Asia, India, Africa, Europe and the Americas as area coding.

Sciuridae (Mammalia, Rodentia): We re-analysed the data set of Lu *et al.*[53] on flying squirrels (subfamily Pteromyinae), including the *IRBP* gene, and partial *12S* and *16S rRNA* gene sequences; 2,584 bp total alignment length). We calibrated the MRCA of the tribe Pteromyini with the first appearance date of the pteromyine fossil *Oligopetes* spp. from southern Germany in the Early Oligocene (MP21, 33.9–32.6 Ma)[54]. We translated this date into a lognormal calibration density with an offset of 32.6 Ma and a 5–95% interquantile range of 33.1–46.7 Ma. The resulting rates were 0.45% per Ma (95% CI = 0.34–0.56% per Ma) for the *12S rRNA*, 0.39% per Ma (95% CI = 0.29–0.50% per Ma) for the *16S rRNA*, and 0.08% per Ma (95% CI = 0.06–0.09% per Ma) for the *IRBP* gene. For the biogeographical inference, we used East/SE Asia, the Palearctic, India and the Americas as area coding.

Tupaiidae (Mammalia, Scandentia): We re-analysed the data set of Roberts *et al.*[55] using the alignment provided by the authors and also applied the fossil calibration points as suggested by the authors. However, we were unable to run the analysis using all suggested calibration points. Therefore, we reduced the number of calibration points to the two ingroup calibrations (lognormal calibration densities with mean and SD = 1.0; tMRCA [*Tupaia*, *Urogale* and *Anathana*] = 18.0 Ma, and tMRCA of the order Scandentia = 38.2 Ma as offset points), and also used a re-aligned data set without excluding the more variable parts of the sequences (*12S rRNA*, *16S rRNA*, *tRNA Leu*, *tRNA Val* and *tRNA Phe*

genes; 2,849 bp total alignment length). Credibility intervals of divergence events overlap with those given by Roberts *et al.*[55], but the retrieved mean values were considerably younger. The resulting rates were 0.86% per Ma (95% CI = 0.70–1.02% per Ma) for the *12S rRNA* gene, 1.18% per Ma (95% CI = 0.99–1.38% per Ma) for the *16S rRNA* gene, 0.85% per Ma (95% CI = 0.07–3.0% per Ma) for the *tRNA Leu* gene, 1.28% per Ma (95% CI = 0.49–2.09% per Ma) for the *tRNA Phe* gene, and 1.62% per Ma (95% CI = 0.96–2.36% per Ma) for the *tRNA Val* gene. For the biogeographical inference, we used mainland Asia, India, and the Philippines as area coding. Dispersal to the Indian subcontinent was inferred at the MRCA of the genera *Dendrogale* and *Tupaia*.

Colobinae (Mammalia, Primates): We re-analysed the phylogeny by He *et al.*[56] that used 1,140 bp sequence information of *Cytb* and 376 bp of the nuclear *PRM1* gene to infer the phylogeny and divergence times for the genus *Trachypithecus* in the subfamily Colubinae. To calibrate the phylogeny, we applied a secondary calibration point based on the study of He *et al.*[56] for the MRCA of Colobinae using a normal calibration density (mean = 12.28 Ma, SD = 1.74, 5–95% interquantile range = 9.4–15.1 Ma). Resulting mean substitution rates were 1.74% per Ma (95% CI = 1.17–2.36% per Ma) for *Cytb*, and 0.16% per Ma (95% CI = 0.05–0.35% per Ma) for *PRM1*. For the biogeographical inference, we used mainland Asia, India, and Africa as area coding.

Supplementary References

1. Rutschmann, F., Eriksson, T., Salim, K. A. & Conti, E. Assessing calibration uncertainty in molecular dating: the assignment of fossils to alternative calibration points. *Syst. Biol.* **56,** 591–608 (2007).
2. Gamage, D. T., Silva, M. P. de, Inomata, N., Yamazaki, T. & Szmidt, A. E. Comprehensive molecular phylogeny of the sub-family Dipterocarpoideae (Dipterocarpaceae) based on chloroplast DNA sequences. *Genes. Genet. Syst.* **81,** 1–12 (2006).
3. Gunasekara, N. Masters-thesis. Concordia University, 01.01.2004.
4. Bancroft, H. The taxonomic history and geographical distribution of the Monotoideae. *Am. J. Bot.,* 505–519 (1935).
5. Dutta, S. *et al.* Eocene out-of-India dispersal of Asian dipterocarps. *Rev. Palaeobot. Palyno.* **166,** 63–68 (2011).
6. Yuan, Y.-M. *et al.* Phylogeny and biogeography of *Exacum* (Gentianaceae): a disjunctive distribution in the Indian Ocean Basin resulted from long distance dispersal and extensive radiation. *Syst. Biol.* **54,** 21–34 (2005).
7. Hagen, K. B. von & Kadereit, J. W. The phylogeny of *Gentianella* (Gentianaceae) and its colonization of the southern hemisphere as revealed by nuclear and chloroplast DNA sequence variation. *Org. Divers. Evol.* **1,** 61–79 (2001).
8. Hagen, K. B. von & Kadereit, J. W. Phylogeny and flower evolution of the Swertiinae (Gentianaceae-Gentianeae): Homoplasy and the principle of variable proportions. *Syst. Bot.* **27,** 548–572 (2002).
9. Koehler, F. & Glaubrecht, M. Out of Asia and into India: on the molecular phylogeny and biogeography of the endemic freshwater gastropod *Paracrostoma* Cossmann, 1900 (Caenogastropoda: Pachychilidae). *Biol. J. Linn. Soc.* **91,** 627–651 (2007).
10. Hellberg, M. E. & Vacquier, V. D. Rapid evolution of fertilization selectivity and lysin cDNA sequences in teguline gastropods. *Mol. Biol. Evol.* **16,** 839–848 (1999).

11. Marko, P. B. Fossil calibration of molecular clocks and the divergence times of geminate species pairs separated by the Isthmus of Panama. *Mol. Biol. Evol.* **19,** 2005–2021 (2002).

12. Klaus, S., Schubart, C. D., Streit, B. & Pfenninger, M. When Indian crabs were not yet Asian-biogeographic evidence for Eocene proximity of India and Southeast Asia. *BMC Evol. Biol.* **10,** 287 (2010).

13. Klaus, S., Fernandez, K. & Yeo, D. C. J. Phylogeny of the freshwater crabs of the Western Ghats (Brachyura, Gecarcinucidae). *Zool. Scripta* **43,** 651–660 (2014).

14. Daniels, S. R., Phiri, E. E., Klaus, S., Albrecht, C. & Cumberlidge, N. Multilocus phylogeny of the Afrotropical freshwater crab fauna reveals historical drainage connectivity and transoceanic dispersal since the Eocene. *Syst. Biol.,* syv011 (2015).

15. Condamine, F. L., Sperling, F. A. H. & Kergoat, G. J. Global biogeographical pattern of swallowtail diversification demonstrates alternative colonization routes in the Northern and Southern hemispheres. *J. Biogeogr.* **40,** 9–23 (2013).

16. Rehan, S. M. *et al.* Molecular phylogeny of the small carpenter bees (Hymenoptera: Apidae: Ceratinini) indicates early and rapid global dispersal. *Mol. Phylogenet. Evol.* **55,** 1042–1054 (2010).

17. Ward, P. S. & Downie, D. A. The ant subfamily Pseudomyrmecinae (Hymenoptera: Formicidae): phylogeny and evolution of big-eyed arboreal ants. *Syst. Entomol.* **30,** 310–335 (2005).

18. Jansen, G., Savolainen, R. & Vepsäläinen, K. Phylogeny, divergence-time estimation, biogeography and social parasite-host relationships of the Holarctic ant genus *Myrmica* (Hymenoptera: Formicidae). *Mol. Phylogenet. Evol.* **56,** 294–304 (2010).

19. Murphy, W. J. & Collier, G. E. A molecular phylogeny for aplocheiloid fishes (Atherinomorpha, Cyprinodontiformes): the role of vicariance and the origins of annualism. *Mol. Biol. Evol.* **14,** 790–799 (1997).

20. Adamson, E. A. S., Hurwood, D. A. & Mather, P. B. A reappraisal of the evolution of Asian snakehead fishes (Pisces, Channidae) using molecular data

from multiple genes and fossil calibration. *Mol. Phylogenet. Evol.* **56,** 707–717 (2010).

21. Ratmuangkhwang, S., Musikasinthorn, P. & Kumazawa, Y. Molecular phylogeny and biogeography of air sac catfishes of the *Heteropneustes fossilis* species complex (Siluriformes: Heteropneustidae). *Mol. Phylogenet. Evol.* **79,** 82–91 (2014).
22. Nakatani, M., Miya, M., Mabuchi, K., Saitoh, K. & Nishida, M. Evolutionary history of Otophysi (Teleostei), a major clade of the modern freshwater fishes: Pangaean origin and Mesozoic radiation. *BMC Evol. Biol.* **11,** 177 (2011).
23. Rüber, L., Britz, R. & Zardoya, R. Molecular phylogenetics and evolutionary diversification of labyrinth fishes (Perciformes: Anabantoidei). *Syst. Biol.* **55,** 374–397 (2006).
24. Šlechtová, V., Bohlen, J. & Perdices, A. Molecular phylogeny of the freshwater fish family Cobitidae (Cypriniformes: Teleostei): delimitation of genera, mitochondrial introgression and evolution of sexual dimorphism. *Mol. Phylogenet. Evol.* **47,** 812–831 (2008).
25. Doadrio, I. & Perdices, A. Phylogenetic relationships among the Ibero-African cobitids (*Cobitis*, Cobitidae) based on cytochrome b sequence data. *Mol. Phylogenet. Evol.* **37,** 484–493 (2005).
26. Pyron, R. A. & Wiens, J. J. A large-scale phylogeny of Amphibia including over 2800 species, and a revised classification of extant frogs, salamanders, and caecilians. *Mol. Phylogenet. Evol.* **61,** 543–583 (2011).
27. Kotaki, M. *et al.* Molecular phylogeny of the diversified frogs of genus *Fejervarya* (Anura: Dicroglossidae). *Zool. Sci.* **27,** 386–395 (2010).
28. van Bocxlaer, I., Roelants, K., Biju, S. D., Nagaraju, J. & Bossuyt, F. Late Cretaceous vicariance in Gondwanan amphibians. *PLoS ONE* **1,** e74 (2006).
29. Sá, R. O. de *et al.* Molecular phylogeny of microhylid frogs (Anura: Microhylidae) with emphasis on relationships among New World genera. *BMC Evol. Biol.* **12,** 241 (2012).

30. Li, J.-T. *et al.* Diversification of rhacophorid frogs provides evidence for accelerated faunal exchange between India and Eurasia during the Oligocene. *Proc. Natl. Acad. Sci. U.S.A.* **110,** 3441–3446 (2013).

31. van Bocxlaer, I., Biju, S. D., Loader, S. P. & Bossuyt, F. Toad radiation reveals into-India dispersal as a source of endemism in the Western Ghats-Sri Lanka biodiversity hotspot. *BMC Evol. Biol.* **9,** 131 (2009).

32. Oaks, J. R. A time-calibrated species tree of *Crocodylia* reveals a recent radiation of the true crocodiles. *Evolution* **65,** 3285–3297 (2011).

33. Heled, J. & Drummond, A. J. Bayesian inference of species trees from multilocus data. *Mol. Biol. Evol.* **27,** 570–580 (2010).

34. Brochu, C. A. Morphology, fossils, divergence timing, and the phylogenetic relationships of Gavialis. *Syst. Biol.* **46,** 479–522 (1997).

35. Wood, P. L., Heinicke, M. P., Jackman, T. R. & Bauer, A. M. Phylogeny of bent-toed geckos (*Cyrtodactylus*) reveals a west to east pattern of diversification. *Mol. Phylogenet. Evol.* **65,** 992–1003 (2012).

36. Bansal, R. & Karanth, K. P. Phylogenetic analysis and molecular dating suggest that *Hemidactylus anamallensis* is not a member of the *Hemidactylus* radiation and has an ancient Late Cretaceous origin. *PLoS ONE* **8,** e60615 (2013).

37. Datta-Roy, A., Singh, M., Srinivasulu, C. & Karanth, K. P. Phylogeny of the Asian *Eutropis* (Squamata: Scincidae) reveals an 'into India' endemic Indian radiation. *Mol. Phylogenet. Evol.* **63,** 817–824 (2012).

38. Honda, M. *et al.* Phylogenetic relationships, character evolution, and biogeography of the subfamily Lygosominae (Reptilia: Scincidae) inferred from mitochondrial DNA sequences. *Mol. Phylogenet. Evol.* **15,** 452–461 (2000).

39. Martin, J. E., Hutchinson, M. N., Meredith, R., Case, J. A. & Pledge, N. S. The oldest genus of scincid lizard (Squamata) from the tertiary Etadunna Formation of South Australia. *J. Herpetol.* **38,** 180–187 (2004).

40. Macey, J. R. *et al.* Evaluating trans-tethys migration: an example using acrodont lizard phylogenetics. *Syst. Biol.* **49,** 233–256 (2000).

41. Townsend, T. M. *et al.* Phylogeny of iguanian lizards inferred from 29 nuclear loci, and a comparison of concatenated and species-tree approaches for an ancient, rapid radiation. *Mol. Phylogenet. Evol.* **61,** 363–380 (2011).

42. Honda, M. *et al.* Phylogenetic Relationships of the Flying Lizards, Genus *Draco* (Reptilia, Agamidae). *Zool. Sci.* **16,** 535–549 (1999).

43. Noonan, B. P. & Chippindale, P. T. Dispersal and vicariance: the complex evolutionary history of boid snakes. *Mol. Phylogenet. Evol.* **40,** 347–358 (2006).

44. Sanders, K. L., Mumpuni, Hamidy, A., Head, J. J. & Gower, D. J. Phylogeny and divergence times of filesnakes (*Acrochordus*): inferences from morphology, fossils and three molecular loci. *Mol. Phylogenet. Evol.* **56,** 857–867 (2010).

45. Wüster, W., Peppin, L., Pook, C. E. & Walker, D. E. A nesting of vipers: Phylogeny and historical biogeography of the Viperidae (Squamata: Serpentes). *Mol. Phylogenet. Evol.* **49,** 445–459 (2008).

46. Päckert, M. *et al.* Horizontal and elevational phylogeographic patterns of Himalayan and Southeast Asian forest passerines (Aves: Passeriformes). *J. Biogeogr.* **39,** 556–573 (2012).

47. Päckert, M. *et al.* Calibration of a molecular clock in tits (Paridae)--do nucleotide substitution rates of mitochondrial genes deviate from the 2% rule? *Mol. Phylogenet. Evol.* **44,** 1–14 (2007).

48. Weir, J. T. & Schluter, D. Calibrating the avian molecular clock. *Mol. Ecol.* **17,** 2321–2328 (2008).

49. Groombridge, J. J., Jones, C. G., Nichols, R. A., Carlton, M. & Bruford, M. W. Molecular phylogeny and morphological change in the *Psittacula* parakeets. *Mol. Phylogenet. Evol.* **31,** 96–108 (2004).

50. Gamauf, A., Gjershaug, J.-O., Røev, N., Kvaløy, K. & Haring, E. Species or subspecies? The dilemma of taxonomic ranking of some South-East Asian hawk-eagles (genus *Spizaetus*). *Bird Conserv. Int.* **15,** 99–117 (2005).

51. Hassanin, A. *et al.* Pattern and timing of diversification of Cetartiodactyla (Mammalia, Laurasiatheria), as revealed by a comprehensive analysis of mitochondrial genomes. *C. R. Biol.* **335,** 32–50 (2012).

52. Patou, M.-L. *et al.* Molecular phylogeny of the Herpestidae (Mammalia, Carnivora) with a special emphasis on the Asian *Herpestes*. *Mol. Phylogenet. Evol.* **53,** 69–80 (2009).

53. Lu, X. *et al.* The Evolution and paleobiogeography of flying squirrels (Sciuridae, Pteromyini) in response to global environmental change. *Evol. Biol.* **40,** 117–132 (2013).

54. Heissig, K. Die frühesten Flughörnchen und primitive Ailuravinae (Rodentia, Mamm.) aus dem süddeutschen Oligozän. *Mitt. Bayer. Staatssamml. Palaeontol. Hist. Geol.,* 139–169 (1979).

55. Roberts, T. E., Lanier, H. C., Sargis, E. J. & Olson, L. E. Molecular phylogeny of treeshrews (Mammalia: Scandentia) and the timescale of diversification in Southeast Asia. *Mol. Phylogenet. Evol.* **60,** 358–372 (2011).

56. He, K. *et al.* Molecular phylogeny and divergence time of *Trachypithecus*: with implications for the taxonomy of *T. phayrei*. *Zool. Res.* **33,** E104-10 (2012).
